# Supplementary material for: Cdc14 phosphatase counteracts Cdk-dependent Dna2 phosphorylation to inhibit resection during recombinational DNA repair
Source: Nat Commun. 2023 May 12;14:2738. doi: 10.1038/s41467-023-38417-5 (PMC10182099; doi:10.1038/s41467-023-38417-5)
Supplement: Supplementary file 1 — Supplementary Information [file 41467_2023_38417_MOESM1_ESM.pdf]

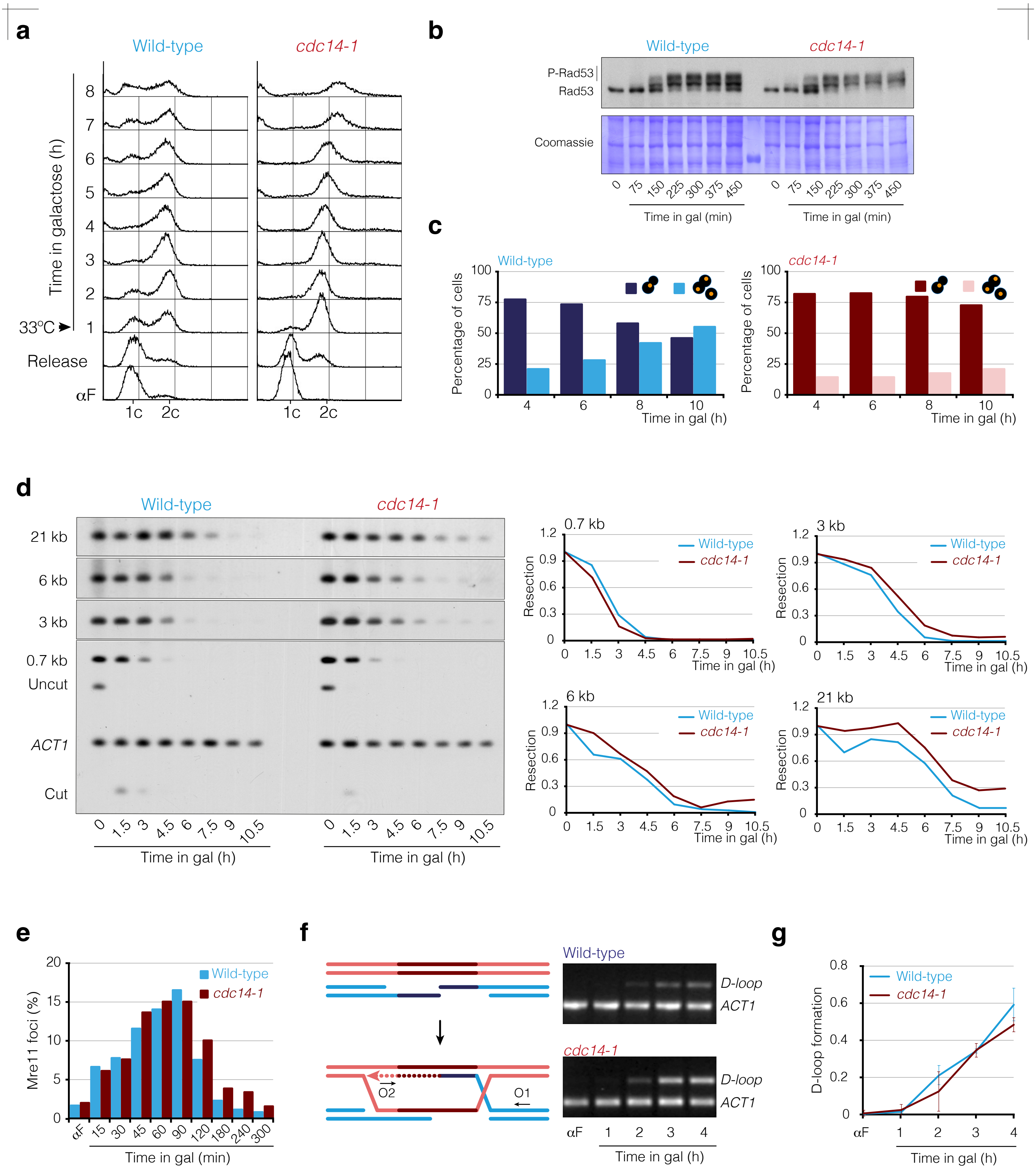
a
Wild-type
cdc14-1
Time in galactose (h)
8
7
6
5
4
3
2
33ºC 1
Release
αF
1c 2c
1c 2c
b
Wild-type
cdc14-1
P-Rad53
Rad53
Coomassie
0 75 150 225 300 375 450
0 75 150 225 300 375 450
Time in gal (min)
Time in gal (min)
c
Wild-type
cdc14-1
Percentage of cells
100 75 50 25 0
4 6 8 10
Time in gal (h)
d
Wild-type
cdc14-1
21 kb
6 kb
3 kb
0.7 kb
Uncut
ACT1
Cut
0 1.5 3 4.5 6 7.5 9 10.5
Time in gal (h)
0.7 kb
3 kb
6 kb
21 kb
Resection
1.2 0.9 0.6 0.3 0
Wild-type
cdc14-1
e
Mre11 foci (%)
20 15 10 5 0
Wild-type
cdc14-1
αF 15 30 45 60 90 120 180 240 300
Time in gal (min)
f
O2
O1
Wild-type
D-loop
ACT1
cdc14-1
D-loop
ACT1
αF 1 2 3 4
Time in gal (h)
g
D-loop formation
0.8 0.6 0.4 0.2 0
Wild-type
cdc14-1
αF 1 2 3 4
Time in gal (h)

Supp. Fig. 1. Cdc14 is not required either for end resection or D-loop extension but is required for DNA repair

a FACS profile of the DNA content of wild-type and *cdc14-1* samples collected from the experiment shown in Fig. **1c**. **b** Samples collected under the same experimental conditions shown in Fig. **1c** were extracted with TCA and subjected to western blotting. Coomassie staining is shown as a loading control. **c** Cells collected under the same experimental conditions shown in Fig. **1c** were fixed, stained with DAPI, and analyzed by microscopy. The percentages of cells in metaphase (dark blue/red) and in anaphase/G1 (light blue/red) are shown. **d** Analysis of resection in wild-type and *cdc14-1* JKM179 cells containing a non-repairable HO break. Cells were induced with galactose, and samples were taken at the indicated time points to analyze resection proficiency by Southern blot as in Fig. **2a**. The graphs on the right represent the quantification of the Southern blot band signals after normalization against the *ACT1* and the uncut T0 sample. **e** Mre11-GFP foci formation and dissolution in wild-type and *cdc14-1* strains were determined by measuring the number of foci in alpha factor pre-synchronized G1 cells released in the presence of galactose to induce the HO break. Samples were taken at the indicated time points and analyzed by fluorescence microscopy. **f** PCR approach to detect new DNA synthesis primed by the 3′ invading end after D-loop formation. Dark blue and red represent homologous *MAT*a and *MAT*a′ sequences, respectively. O1 and O2 denote the pair of oligonucleotides used in the analysis. A pair of oligonucleotides in the *ACT1* locus was used as a loading control. **g** Real time PCR from the same samples analyzed in **f**. The graph shows the mean ± SD from two biologically independent experiments.

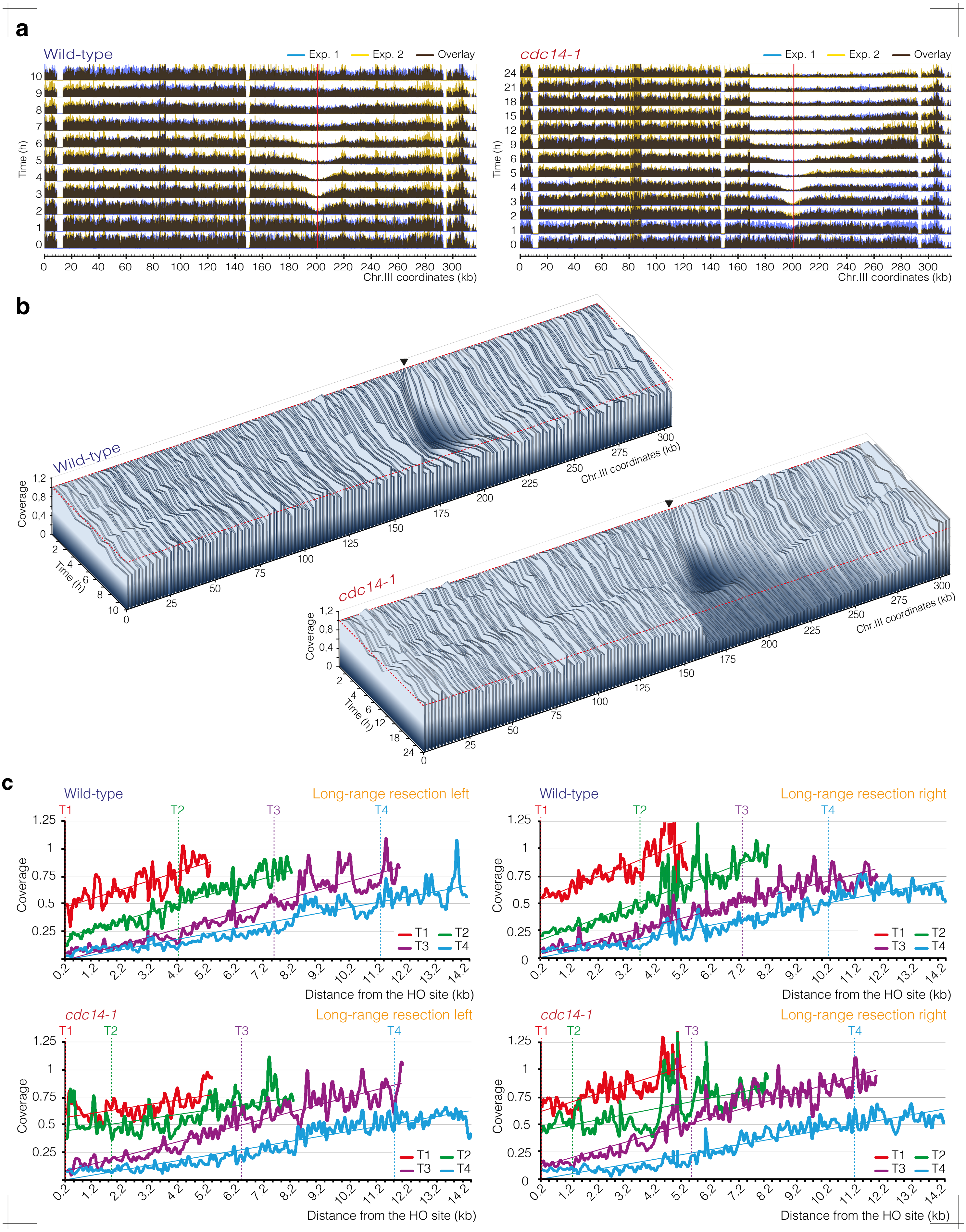
a
Wild-type
Exp. 1
Exp. 2
Overlay
Time (h)
Chr.III coordinates (kb)
cdc14-1
b
Wild-type
Coverage
Time (h)
Chr.III coordinates (kb)
cdc14-1
c
Wild-type
Long-range resection left
Long-range resection right
cdc14-1
T1
T2
T3
T4
Coverage
Distance from the HO site (kb)

Supp. Fig. 2. Genome-wide sequencing analysis of DNA repair in the absence of Cdc14 activity

a Read coverage profiles of chromosome III in wild-type (left panel) and *cdc14-1* (right panel) PMV cells after HO induction. DNA extracted from the experiment shown in Fig. **1c** was used to generate genomic libraries for sequencing. Red lines indicate the position of the HO cleavage site in *MAT*a at position 200753. Blue and yellow profiles depict read coverage from two biologically independent experiments. Black indicates overlapping between experiments. **b** Graphs from Fig. **3a** were compiled to generate normalized 3D coverage profiles of wild-type and *cdc14-1* strains, simultaneously representing coverage levels of each sample time normalized to T0 (y-axis), the coordinates along the entire chromosome III (x-axis), and the time after HO induction (z-axis). The read coverage values were averaged in 2 kb sections and normalized against the same section of the 0 h sample and to a 120 kb region between coordinates 210000 and 330000 of chromosome V. A coverage level of 1 is denoted with a red dotted line. Light and dark blue areas represent high- and low-coverage levels, respectively. The black triangles mark the position of the HO-induced break. The graphs show the mean from two biologically independent experiments. **c** Normalized read coverage profiles of wild-type (top panels) and *cdc14-1* (bottom panels) PMV cells of a 15 kb region to the left (left panels) and right (right panels) from the HO cleavage site between 1 and 4 h after the endonuclease induction. The values were normalized against the T0 sample, averaged in 100 nt bins, and plotted. A trend line for each time sample is shown. The trend line linear equations ($y = mx + b$) for each time point sample were used with a fixed y-value of 0.5 (50% coverage) to determine the x-value (kb from the HO cleavage site). Vertical dotted lines mark the distance from the HO cleavage site where read coverage drops to 50%, as a result of resection progression. The graphs show the mean from two biologically independent experiments.

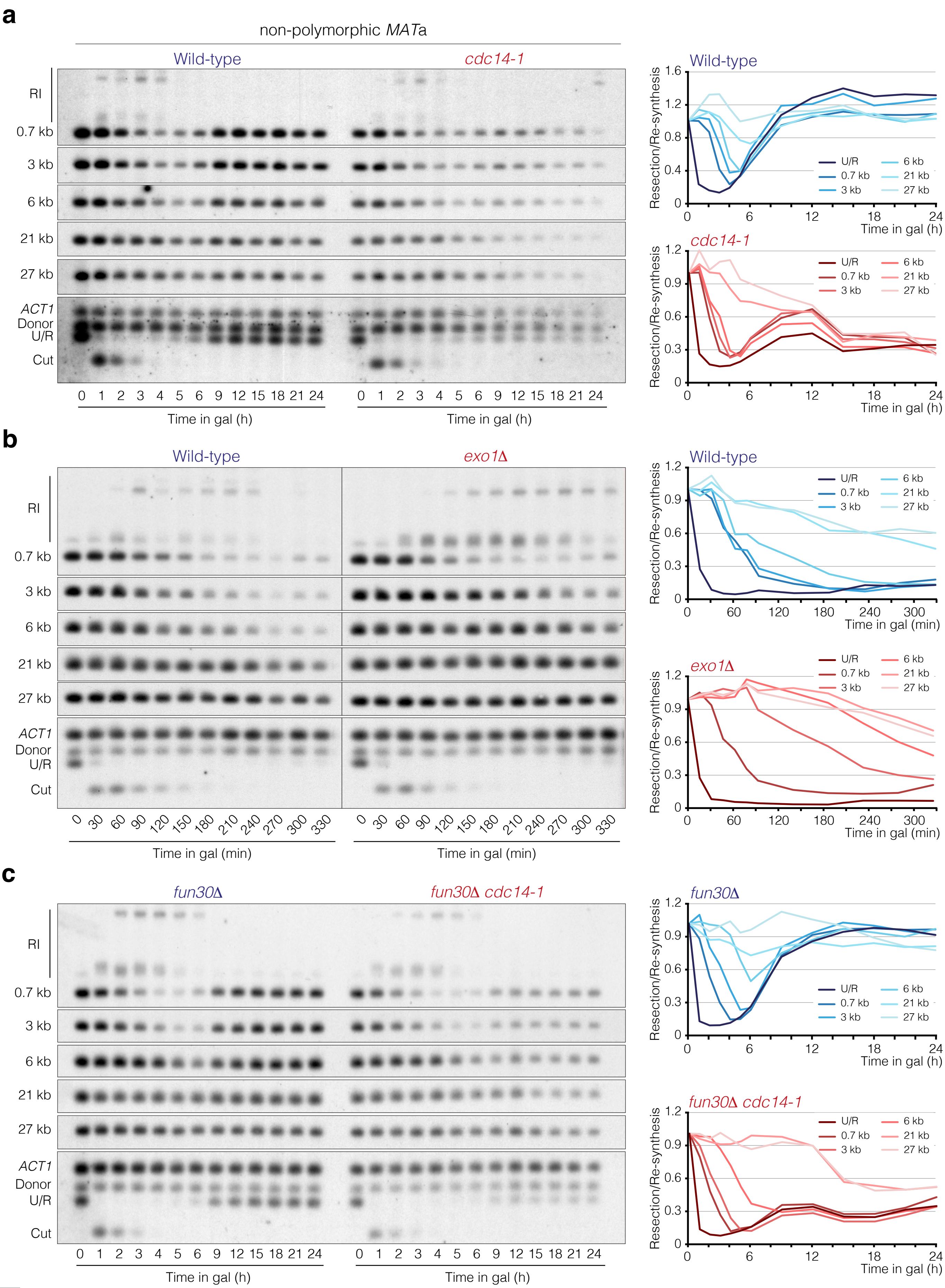
a
non-polymorphic MATa
Wild-type
cdc14-1
RI
0.7 kb
3 kb
6 kb
21 kb
27 kb
ACT1
Donor
U/R
Cut
0 1 2 3 4 5 6 9 12 15 18 21 24
Time in gal (h)
Resection/Re-synthesis
U/R
0.7 kb
3 kb
6 kb
21 kb
27 kb
b
Wild-type
exo1Δ
0 30 60 90 120 150 180 210 240 270 300 330
Time in gal (min)
c
fun30Δ
fun30Δ cdc14-1

Supp. Fig. 3. Over-resection in the absence of Cdc14 is not due to the presence of a polymorphic *MAT*a′ donor sequence

a Southern blot analysis of wild-type and *cdc14-1* strains carrying a non-polymorphic *MAT*a HO-*inc* donor sequence in the *ARG5,6 locus* in chromosome V. Cells were grown in YP-Raffinose at 25ºC before inducing HO expression and transferring the cultures to 33ºC. Samples were taken at different intervals, and genomic DNA was extracted, digested with *Sty*I, and analyzed by Southern blot using probes located at increasing distances from the HO cleavage site as depicted in Fig. **2a**. An *ACT1* probe was used as a loading control. The graphs on the right represent the quantification of the averaged band signals from two Southern blots after normalization against their respective *ACT1* signal and uncut T0 sample. RI: resection intermediates. U/R: Uncut/Repair. **b** Southern blot analysis of resection and DNA repair in wild-type and *exo1Δ* PMV cells. Cultures were grown in YP-Raffinose at 28ºC before inducing HO expression. Samples were taken at different intervals, and genomic DNA was extracted, digested with *Sty*I, and analyzed by Southern blot using probes located at increasing distances from the HO cleavage site as depicted in Fig. **2a**. An *ACT1* probe was used as a loading control. The graphs on the right represent the quantification of the Southern blot band signals after normalization against the *ACT1* and the uncut T0 sample. RI: resection intermediates. U/R: Uncut/Repair. **c** Southern blot using the same experimental conditions as in **a** in *fun30Δ* and *cdc14-1 fun30Δ* PMV cells. The graphs on the right represent the quantification of the averaged band signals from two Southern blots after normalization against their respective *ACT1* signal and uncut T0 sample. RI: resection intermediates. U/R: Uncut/Repair.

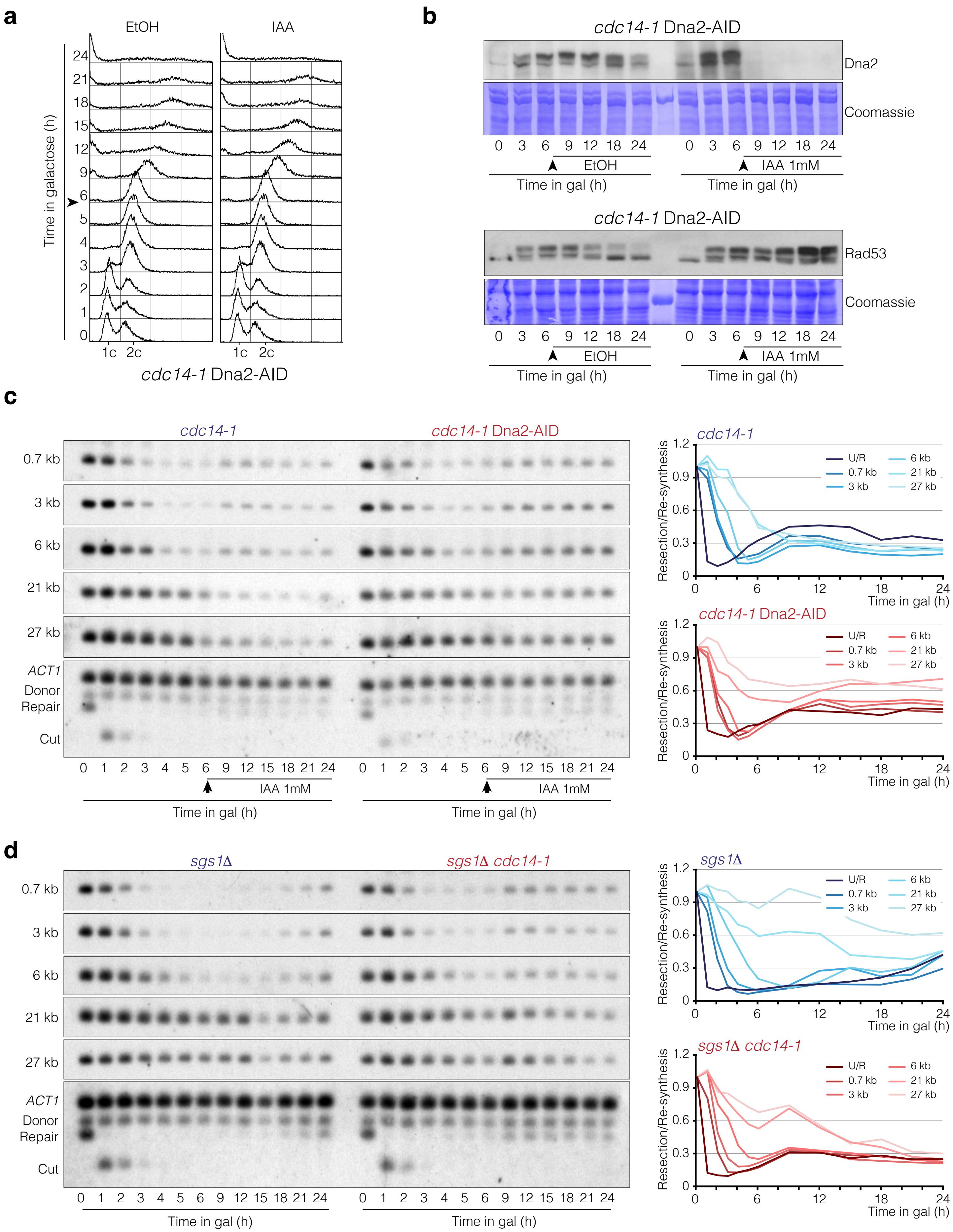
a
EtOH
IAA
Time in galactose (h)
24
21
18
15
12
9
6
5
4
3
2
1
0
1c 2c
1c 2c
cdc14-1 Dna2-AID
b
cdc14-1 Dna2-AID
Dna2
Coomassie
0 3 6 9 12 18 24
EtOH
Time in gal (h)
0 3 6 9 12 18 24
IAA 1mM
Time in gal (h)
cdc14-1 Dna2-AID
Rad53
Coomassie
0 3 6 9 12 18 24
EtOH
Time in gal (h)
0 3 6 9 12 18 24
IAA 1mM
Time in gal (h)
c
cdc14-1
cdc14-1 Dna2-AID
0.7 kb
3 kb
6 kb
21 kb
27 kb
ACT1
Donor
Repair
Cut
0 1 2 3 4 5 6 9 12 15 18 21 24
IAA 1mM
Time in gal (h)
0 1 2 3 4 5 6 9 12 15 18 21 24
IAA 1mM
Time in gal (h)
cdc14-1
Resection/Re-synthesis
U/R
0.7 kb
3 kb
6 kb
21 kb
27 kb
Time in gal (h)
cdc14-1 Dna2-AID
Resection/Re-synthesis
U/R
0.7 kb
3 kb
6 kb
21 kb
27 kb
Time in gal (h)
d
sgs1Δ
sgs1Δ cdc14-1
0.7 kb
3 kb
6 kb
21 kb
27 kb
ACT1
Donor
Repair
Cut
0 1 2 3 4 5 6 9 12 15 18 21 24
Time in gal (h)
0 1 2 3 4 5 6 9 12 15 18 21 24
Time in gal (h)
sgs1Δ
Resection/Re-synthesis
U/R
0.7 kb
3 kb
6 kb
21 kb
27 kb
Time in gal (h)
sgs1Δ cdc14-1
Resection/Re-synthesis
U/R
0.7 kb
3 kb
6 kb
21 kb
27 kb
Time in gal (h)

Supp. Fig. 4. Inactivation of Dna2 alleviates over-resection in the absence of Cdc14 activity

a FACS profile of the DNA content of *cdc14-1* Dna2-AID cells from samples taken in the experiment shown in Fig. **4c**. The addition of mock ethanol (left) or auxin (right) to the culture is marked with a black arrow. **b** Western blot of samples collected at the indicated time points from the experiment shown in Fig. **4c** to assess Dna2 degradation after auxin addition (top panel) and Rad53 phosphorylation (bottom panel). Black arrows mark the addition of mock ethanol or auxin to the culture. Coomassie blue staining is shown as a loading control. **c** Comparison of the resection profile of *cdc14-1* and *cdc14-1* Dna2-AID PMV strains by Southern blot. After overnight culture in YP-Raffinose at 25ºC, HO was induced with galactose and the cultures were transferred to 33ºC. Six hours after the HO induction auxin was added to the media, and samples were taken at different intervals, genomic DNA was extracted, digested with *Sty*I, and analyzed by Southern blot using probes located at increasing distances from the HO cleavage site as depicted in Fig. **2a**. An *ACT1* probe was used as a loading control. The graphs on the right represent the quantification of the averaged band signals from two Southern blots after normalization against their respective *ACT1* signal and uncut T0 sample. U/R: Uncut/Repair. **d** Southern blot analysis of resection and DNA repair in *sgs1Δ* and *sgs1Δ cdc14-1* PMV cells. HO was expressed with galactose in cells growing in YP-Raffinose at 25ºC and transferred to 33ºC. Samples were taken at different intervals, and genomic DNA was extracted, digested with *Sty*I, and analyzed by Southern blot as in **c**. The graphs on the right represent the quantification of the averaged band signals from two Southern blots after normalization against their respective *ACT1* signal and uncut T0 sample. U/R: Uncut/Repair.

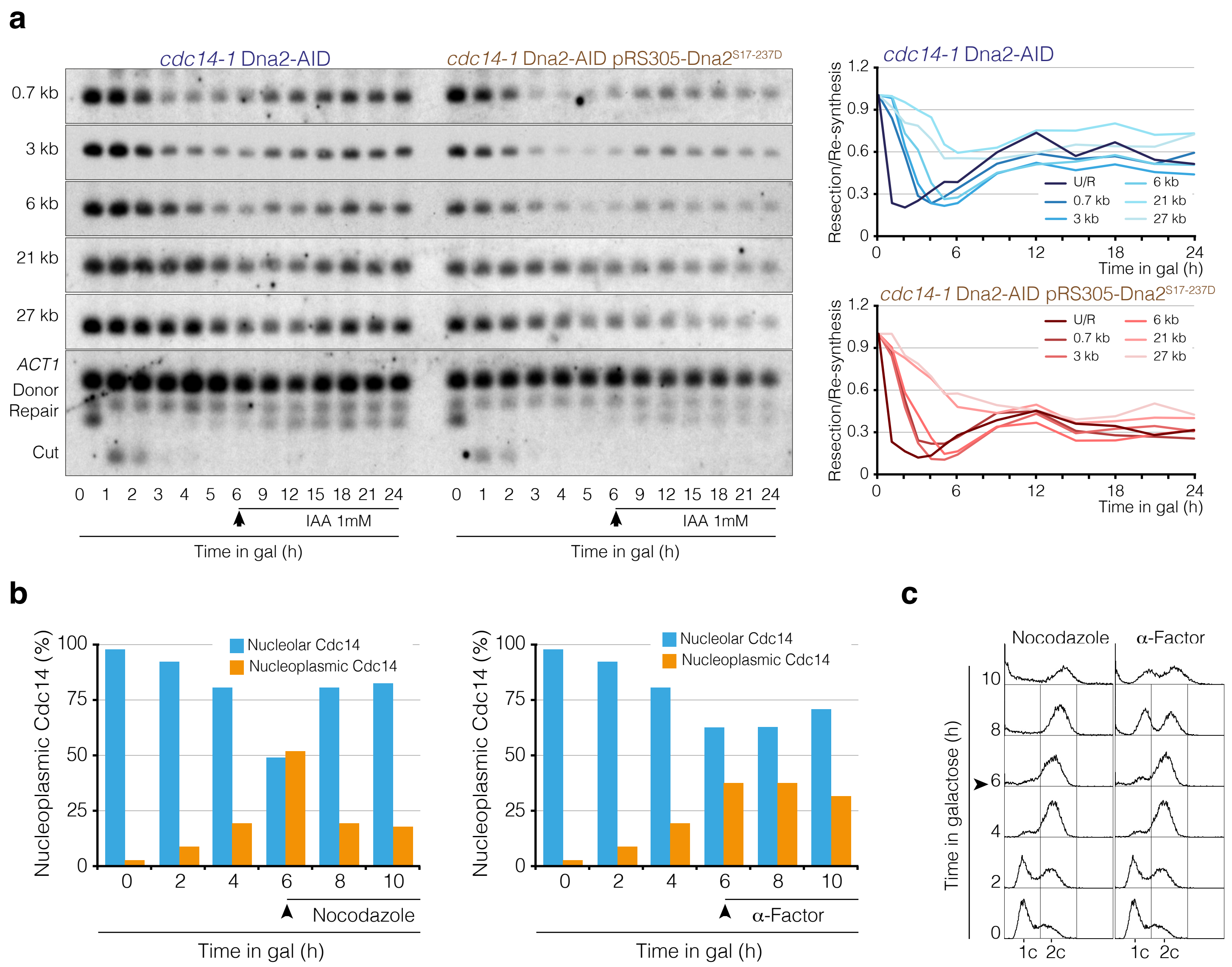
a
cdc14-1 Dna2-AID
cdc14-1 Dna2-AID pRS305-Dna2S17-237D
0.7 kb
3 kb
6 kb
21 kb
27 kb
ACT1
Donor
Repair
Cut
0 1 2 3 4 5 6 9 12 15 18 21 24
IAA 1mM
Time in gal (h)
Resection/Re-synthesis
U/R
0.7 kb
3 kb
6 kb
21 kb
27 kb
Time in gal (h)
b
Nucleoplasmic Cdc14 (%)
Nucleolar Cdc14
Nucleoplasmic Cdc14
Nocodazole
α-Factor
Time in gal (h)
c
Nocodazole
α-Factor
Time in galactose (h)
1c 2c

Supp. Fig. 5. Cdc14 is implicated in DNA re-synthesis

a Comparison of the resection profile of *cdc14-1* Dna2-AID and *cdc14-1* Dna2-AID pRS305-Dna2$^{S17-237D}$ strains by Southern blot. After overnight culture in YP-Raffinose at 25ºC, HO was induced with galactose and the cultures were transferred to 33ºC. Six hours after HO induction, auxin was added to inactivate the endogenous Dna2-AID. Samples were taken before and after the addition of auxin, and genomic DNA was extracted, digested with *Sty*I, and analyzed by Southern blot using probes located at increasing distances from the HO cleavage site as depicted in Fig. **2a**. An *ACT1* probe was used as a loading control. The graphs on the right represent the quantification of the averaged band signals from two Southern blots after normalization against their respective *ACT1* signal and uncut T0 sample. U/R: Uncut/Repair. **b** PMV cells expressing a Cdc14-GFP version under its own promoter were grown under the same experimental conditions as in Fig. **6b**, and samples were collected at the indicated time points to follow Cdc14 localization after adding nocodazole (left) or alpha factor (right). Blue represents cells with an exclusive nucleolar signal of Cdc14. Orange represents cells with nucleoplasmic accumulation of Cdc14. **c** FACS profile of the experiment shown in **b**. The arrow indicates the addition of nocodazole (left) or alpha factor (right) to the culture.

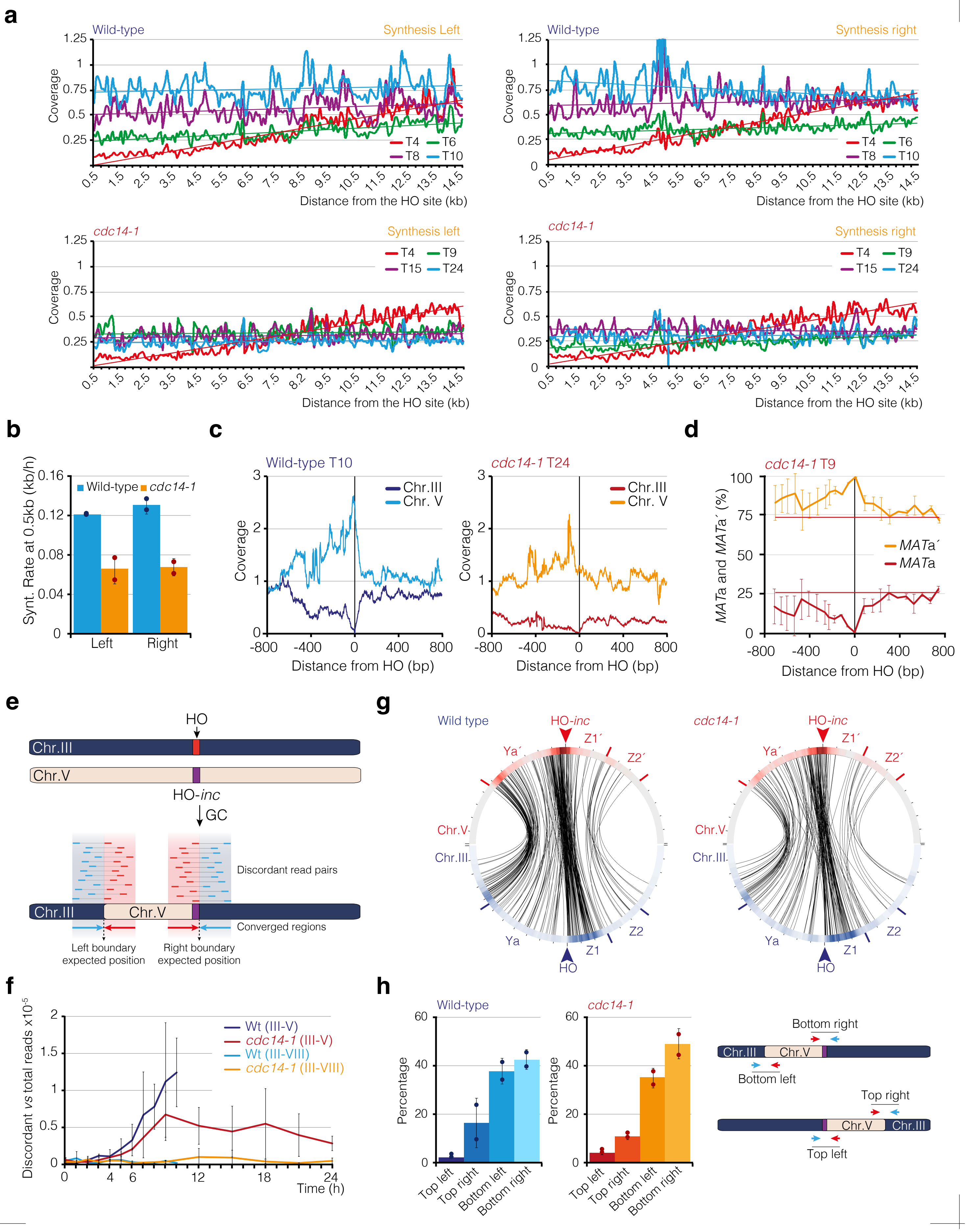

a
Wild-type
Synthesis Left
Synthesis right
Coverage
T4 T6 T8 T10
Distance from the HO site (kb)
cdc14-1
Synthesis left
Synthesis right
T4 T9 T15 T24
b
Synt. Rate at 0.5kb (kb/h)
Wild-type cdc14-1
Left
Right
c
Wild-type T10
cdc14-1 T24
Chr.III
Chr. V
Coverage
Distance from HO (bp)
d
cdc14-1 T9
MATa and MATa´ (%)
MATa´
MATa
Distance from HO (bp)
e
HO
Chr.III
Chr.V
HO-inc
GC
Discordant read pairs
Converged regions
Left boundary expected position
Right boundary expected position
f
Discordant vs total reads x10^-5
Wt (III-V)
cdc14-1 (III-V)
Wt (III-VIII)
cdc14-1 (III-VIII)
Time (h)
g
Wild type
cdc14-1
HO-inc
Ya´
Z1´
Z2´
Chr.V
Chr.III
Ya
Z1
Z2
HO
h
Wild-type
cdc14-1
Percentage
Top left
Top right
Bottom left
Bottom right

Supp. Fig. 6. Cdc14 cooperates in the extension of GC events

a Normalized read coverage profiles of wild-type (top panels) and *cdc14-1* (bottom panels) PMV cells of a 15 kb region to the left (left panels) and right (right panels) of the HO cleavage site between 4h and 10h/24h after HO induction. A trend line for each time sample is shown (coverage profiles for T5, T7, and T9 samples in the wild-type, and T5, T6, T12, T18, and T21 in the *cdc14-1* mutant are not shown for simplicity). The graphs show the mean from two biologically independent experiments. **b** DNA re-synthesis initiation rate in wild-type and *cdc14-1* PMV cells measured at 0.5 kb from the HO-induced break. The graph shows the mean ± SD from two biologically independent experiments. **c** Normalized read coverage profiles of ± 800 nt across the HO (*MAT*a *locus*) and HO-*inc* (*MAT*a′ *locus*) sites after 10 h in the wild-type (left panel) and 24 h in the *cdc14-1* mutant (right panel) from the HO induction. **d** Relative proportion of reads containing polymorphisms in the *MAT*a and *MAT*a′ regions of chromosomes III and V after 9 h from the HO induction in *cdc14-1* cells. Horizontal red lines mark the expected profile of polymorphisms in *MAT*a and *MAT*a′ considering the total DNA repair levels obtained by 9 h. The graph shows the mean ± SD from two biologically independent experiments. **e** Schematic representation of the strategy used to determine the extent and directionality of GC by analyzing the distribution of the discordant read pairs along the *MAT*a *locus* after the repair of the HO-induced break. Blue and red lines represent the two discordant reads obtained from each paired end sequencing event. Grey lines connecting discordant reads represent unsequenced DNA regions. Note that the positions of the left and right boundaries are inferred from the intersection of the regions obtained during the alignment of the discordant read pairs (blue and red areas). **f** Quantification of the proportion of discordant *MAT*a-*MAT*a′ reads pairs in relation to the total number of read pairs obtained in wild-type (dark blue) and *cdc14-1* cells (dark red) after HO induction. Analysis of the proportion of discordant read pairs between chromosomes III and VIII in the wild-type (light blue) and *cdc14-1* (orange) strains is shown as a negative control. Graph shows the mean ± SD from two biologically independent experiments. **g** Circus diagram showing the distribution of the discordant *MAT*a-*MAT*a′ read pairs in the wild-type (left panel) and *cdc14-1* mutant (right panel) obtained from the entire T0 to T10/T24 set of samples. A densitometry graph to facilitate visualization of the gathering of *MAT*a (blue) and *MAT*a′ (red) discordant reads pairs at the GC boundaries is shown. **h** Quantification of the number of discordant read pairs detected in each HO/HO-*inc* quadrant shown in Fig. **8c** in a wild-type (left panel) and a *cdc14-1* mutant (right panel). A diagram depicting the most probable localization of the discordant read pairs falling in each quadrant around the GC boundaries is shown. The graphs show the mean ± SD from two biologically independent experiments.

Supplemental Tables

Supplemental Table 1. Genotypes of strains used in this study

Strain	Genotype	Reference
AC406	*MAT*a HOΔ *ade1-100 leu2,3-112 lys5 ura3-52 trp1*::hisG *hml*::ADE1 *hmr*::ADE1 *ade3*::GAL-HO	Lee *et al.*, 1998
AC1595	*MAT*a HOΔ *ade1-100 leu2,3-112 lys5 ura3-52 trp1*::hisG *hml*::ADE1 *hmr*::ADE1 *ade3*::GAL-HO *arg5,6*::Ya'-HO-inc-Z1-Z2'	This Study
AC1638	*MAT*a HOΔ *ade1-100 leu2,3-112 lys5 ura3-52 trp1::*hisG *hml*::ADE1 *hmr*::ADE1 *ade3*::GAL-HO *arg5,6*::Ya'-HO-inc-Z1-Z2' Cdc14-GFP::KanMX	This Study
AC1640	*MAT*a HOΔ *ade1-100 leu2,3-112 lys5 ura3-52 trp1::*hisG *hml*::ADE1 *hmr*::ADE1 *ade3*::GAL-HO *arg5,6*::Ya'-HO-inc-Z1-Z2' Cdc14-9Myc::HPH	This Study
AC1642	*MAT*a HOΔ *ade1-100 leu2,3-112 lys5 ura3-52 trp1::*hisG *hml*::ADE1 *hmr*::ADE1 *ade3*::GAL-HO *arg5,6*::Ya'-HO-inc-Z1-Z2' *cdc14-1*-9Myc::HPH	This Study
AC1726	*MAT*a HOΔ *ade1-100 leu2,3-112 lys5 ura3-52 trp1*::hisG *hml*::ADE1 *hmr*::ADE1 *ade3*::GAL-HO *arg5,6*::Ya'-HO-inc-Z1-Z2' *sgs1Δ::NAT*	Ramos *et al.*, 2022
AC1796	*MAT*a HOΔ *ade1-100 leu2,3-112 lys5 ura3-52 trp1::*hisG *hml*::ADE1 *hmr*::ADE1 *ade3*::GAL-HO *arg5,6*::Ya'-HO-inc-Z1-Z2' Cdc14-9Myc::HPH Dna2-6HA::Nat	This Study
AC1799	*MAT*a HOΔ *ade1-100 leu2,3-112 lys5 ura3-52 trp1::*hisG *hml*::ADE1 *hmr*::ADE1 *ade3*::GAL-HO *arg5,6*::Ya'-HO-inc-Z1-Z2' *cdc14-1*-9Myc::HPH Dna2-6HA::Nat	This Study
AC1805	*MAT*a HOΔ *ade1-100 leu2,3-112 lys5 ura3-52 trp1*::hisG *hml*::ADE1 *hmr*::ADE1 *ade3*::GAL-HO *arg5,6*::Ya'-HO-inc-Z1-Z2' *cdc14-1*-9Myc::HPH Dna2-GFP::KanMX	This Study
AC1813	*MAT*a HOΔ *ade1-100 leu2,3-112 lys5 ura3-52 trp1*::hisG *hml*::ADE1 *hmr*::ADE1 *ade3*::GAL-HO *arg5,6*::Ya'-HO-inc-Z1-Z2'*, CDC14-9Myc::HPH, Mre11-GFP::KanMX*	This Study
AC1815	*MAT*a HOΔ *ade1-100 leu2,3-112 lys5 ura3-52 trp1*::hisG *hml*::ADE1 *hmr*::ADE1 *ade3*::GAL-HO *arg5,6*::Ya'-HO-inc-Z1-Z2'*, cdc14-1-9Myc::HPH, Mre11-GFP::KanMX*	This Study
AC1822	*HOΔ hml::ADE1 mata::hisG hmr::ADE1 his4::NAT-leu-(Xho- to Asp718) leu2::HOcs ade3::GAL::HO ade1 lys5 ura3-52 trp1::hisG*	Vaze *et al.*, 2002
AC1374	*HOΔ hml::ADE1 mata::hisG hmr::ADE1 his4::NAT-leu-(Xho- to Asp718) leu2::HOcs ade3::GAL::HO ade1 lys5 ura3-52 trp1::hisG cdc14-1*-9Myc::HPH	This Study
AC1917	*MAT*a HOΔ *ade1-100 leu2,3-112 lys5 ura3-52 trp1::*hisG *hml*::ADE1 *hmr*::ADE1 *ade3*::GAL-HO *arg5,6*::Ya'-HO-inc-Z1-Z2' Cdc14-9Myc::HPH *exo1Δ::URA3*	This Study
AC1919	*MAT*a HOΔ *ade1-100 leu2,3-112 lys5 ura3-52 trp1::*hisG *hml*::ADE1 *hmr*::ADE1 *ade3*::GAL-HO *arg5,6*::Ya'-HO-inc-Z1-Z2' *cdc14-1*-9Myc::HPH *exo1Δ::URA3*	This Study
AC1968	*MAT*a HOΔ *ade1-100 leu2,3-112 lys5 ura3-52 trp1*::hisG *hml*::ADE1 *hmr*::ADE1 *ade3*::GAL-HO arg5,6::Ya_HOinc_Z1-Z2	This Study
AC1972	*MAT*a HOΔ *ade1-100 leu2,3-112 lys5 ura3-52 trp1*::hisG *hml*::ADE1 *hmr*::ADE1 *ade3*::GAL-HO arg5,6::Ya_HOinc_Z1-Z2 *cdc14-1*-9Myc::HPH	This Study
AC1982	*MAT*a HOΔ *ade1-100 leu2,3-112 lys5 ura3-52 trp1::*hisG *hml*::ADE1 *hmr*::ADE1 *ade3*::GAL-HO *arg5,6*::Ya'-HO-inc-Z1-Z2' *cdc14-1*-9Myc::HPH Dna2-AID-9Myc::Nat PADH1-TIR::URA3	This Study
AC1998	*MAT*a HOΔ *ade1-100 leu2,3-112 lys5 ura3-52 trp1::*hisG *hml*::ADE1 *hmr*::ADE1 *ade3*::GAL-HO *arg5,6*::Ya'-HO-inc-Z1-Z2' *cdc14-1*-9Myc::HPH Gal-GFP-Cdc14::TRP1	This Study
AC2162	*MAT*a HOΔ *ade1-100 leu2,3-112 lys5 ura3-52 trp1::*hisG *hml*::ADE1 *hmr*::ADE1 *ade3*::GAL-HO *arg5,6*::Ya'-HO-inc-Z1-Z2' *cdc14-1*-9Myc::HPH Dna2-AID-9Myc::Nat PADH1-TIR::URA3 pRS305-pDNA2t::LEU2	This Study
AC2185	*MAT*a HOΔ *ade1-100 leu2,3-112 lys5 ura3-52 trp1::*hisG *hml*::ADE1 *hmr*::ADE1 *ade3*::GAL-HO *arg5,6*::Ya'-HO-inc-Z1-Z2' *cdc14-1*-9Myc::HPH Dna2-AID-9Myc::Nat PADH1-TIR::URA3 pRS305-pDNA2(S17-S237A)t::LEU2	This Study

AC2207	*MAT*a HOΔ *ade1-100 leu2,3-112 lys5 ura3-52 trp1::*hisG *hml*::ADE1 *hmr*::ADE1 *ade3*::GAL-HO *arg5,6*::Ya'-HO-inc-Z1-Z2' *cdc14-1*-9Myc::HPH Dna2-AID-9Myc::Nat PADH1-TIR::URA3 pRS305-pDNA2(S17-S237D)t::LEU2	This Study
AC2385	*MAT*a HOΔ *ade1-100 leu2,3-112 lys5 ura3-52 trp1::*hisG *hml*::ADE1 *hmr*::ADE1 *ade3*::GAL-HO *arg5,6*::Ya'-HO-inc-Z1-Z2' *cdc14-1*-9Myc::HPH Gal-GFP-Cdc14-9MYC::TRP1 Pol12-6HA::Nat	This Study
AC2418	*MAT*a HOΔ *ade1-100 leu2,3-112 lys5 ura3-52 trp1::*hisG *hml*::ADE1 *hmr*::ADE1 *ade3*::GAL-HO *arg5,6*::Ya'-HO-inc-Z1-Z2' *cdc14-1*-9Myc::HPH Dna2-6HA::Nat Gal-GFP-Cdc14-9MYC::TRP1	This Study
AC2498	*MAT*a HOΔ *ade1-100 leu2,3-112 lys5 ura3-52 trp1::*hisG *hml*::ADE1 *hmr*::ADE1 *ade3*::GAL-HO *arg5,6*::Ya'-HO-inc-Z1-Z2' *cdc14-1*-9Myc::HPH 3HA-N-Dna2$_{(1-286)}$::LEU2	This Study
AC2514	*MAT*a HOΔ *ade1-100 leu2,3-112 lys5 ura3-52 trp1*::hisG *hml*::ADE1 *hmr*::ADE1 *ade3*::GAL-HO *arg5,6*::Ya'-HO-inc-Z1-Z2' Dna2-GFP::KanMx	This Study
AC2554	*MAT*a HOΔ *ade1-100 leu2,3-112 lys5 ura3-52 trp1*::hisG *hml*::ADE1 *hmr*::ADE1 *ade3*::GAL-HO *arg5,6*::Ya'-HO-inc-Z1-Z2' 3HA-N-Dna2$_{(1-286)}$::LEU2	This Study
AC2564	*MAT*a HOΔ *ade1-100 leu2,3-112 lys5 ura3-52 trp1*::hisG *hml*::ADE1 *hmr*::ADE1 *ade3*::GAL-HO *arg5,6*::Ya'-HO-inc-Z1-Z2' 3HA-N-Dna2$_{(1-286)}$::LEU2 *cdc5-as1*::Nat	This Study
AC2611	*MAT*a HOΔ *ade1-100 leu2,3-112 lys5 ura3-52 trp1::*hisG *hml*::ADE1 *hmr*::ADE1 *ade3*::GAL-HO *arg5,6*::Ya'-HO-inc-Z1-Z2' *cdc14-1*-9Myc::HPH 3HA-N-Dna2$_{(1-286)}$::LEU2 *cdc5-as1*::Nat	This Study
AC2689	*MAT*a HOΔ *ade1-100 leu2,3-112 lys5 ura3-52 trp1*::hisG *hml*::ADE1 *hmr*::ADE1 *ade3*::GAL-HO *arg5,6*::Ya'-HO-inc-Z1-Z2' *fun30Δ::URA3*	This Study
AC2692	*MAT*a HOΔ *ade1-100 leu2,3-112 lys5 ura3-52 trp1*::hisG *hml*::ADE1 *hmr*::ADE1 *ade3*::GAL-HO *arg5,6*::Ya'-HO-inc-Z1-Z2' *cdc14-1*-9Myc::HPH *fun30Δ::URA3*	This Study
AC2695	*MAT*a HOΔ *ade1-100 leu2,3-112 lys5 ura3-52 trp1*::hisG *hml*::ADE1 *hmr*::ADE1 *ade3*::GAL-HO *arg5,6*::Ya'-HO-inc-Z1-Z2' *sgs1Δ::NAT cdc14-1*-9Myc::HPH	This Study

Supplemental Table 2. Oligonucleotides used for probes synthesis

Name	Sequence	Used in probe for	Background
Act1 probe 1	CGAACAAGAAATGCAAACCGC	*ACT1* (loading control)	PMV
Act1 probe 2	CTTGTGGTGAACGATAGATGG	*ACT1* (loading control)	PMV
Met5 probe Fwd	CCGTTTTACAGGGTGTCTCTA	*MET5* (loading control)	PMV
Met5 probe Rev	ACTTGGCAAAGAGACTAGCAG	*MET5* (loading control)	PMV
Mata Distal 1	CATGCGGTTCACATGACTTTTGAC	*MAT*a-distal (HO repair)	PMV
Mata Distal 2	AGGATGCCCTTGTTTTGTTTACTG	*MAT*a-distal (HO repair)	PMV
-1,5kb HO Fwd	GTCTGTGATGTTGGAGATATG	HO-left probe (HO repair)	PMV
HO -1kb Rev	GGATTCTTATCTAGGGCCAAC	HO-left probe (HO repair)	PMV
+1,5kb HO Fwd	CTTTCCCTGGAAATATCTCCG	HO-right probe (HO repair)	PMV
+2,5kb HO Rev	ACGAATGGGTATATGGGTCTG	HO-right probe (HO repair)	PMV
-5kb HO Fwd	ACACCCAACAAAACACCTGTG	3kb (resection)	PMV
-3,5kb HO Rev	CCTTTTGCTTCTTGTACGCTC	3kb (resection)	PMV
-7,5kb HO Fwd	TGGCCAGAACAATCATGAAGC	6kb (resection)	PMV
-6,25kb HO Rev	TCTTGCTATGGGTGGTATAGC	6kb (resection)	PMV
14kb HO Fwd	TTATGTTGCCAACGGGAGTTC	10kb (resection)	PMV
-12,5kb HO Rev	TCTTCAGGACTTCTTAAGCCG	10kb (resection)	PMV
21kb HO Fwd	TATCTGGGTATATTACCCGGC	21kb (resection)	PMV
21kb HO Rev	TTGTATCCATCGTTTCGGCTG	21kb (resection)	PMV
27kb probe Fwd	GGGGACCATACCTACAATGTA	27kb (resection)	PMV
27kb probe Rev	CATAATTTATGGAGGTGGCGC	27kb (resection)	PMV
U2-1 probe	CCGGTAGTGTTAGACCTGAACAAG	U2 (HO repair)	YMV80
U2-2 probe	TACGTCGTTAAGGCCGTTTCTGAC	U2 (HO repair)	YMV80

27Kb Right HO Fwd	GGACTAGGCTCATCTTTCATC	27kb (resection)	YMV80
27Kb Right HO Rev	AAGACCACTACTTCTGATGCG	27kb (resection)	YMV80
42Kb Right HO Fwd	AATCGTGTGACAACAACAGCC	42kb (resection)	YMV80
42Kb Right HO Rev	AAGGTGACATCCTTACCCAAC	42kb (resection)	YMV80
30Kb Left HO Fwd	AGGAAAATAAGCGGCATGGAC	30kb (resection)	YMV80
30Kb Left HO Rev	TGAATGACATGAAGGAAGGCG	30kb (resection)	YMV80
34Kb Left HO Fwd	TACCCCGGTTTCAGATCTATG	34kb (resection)	YMV80
34Kb Left HO Rev	CAAAACAACCAACGGAAGAGG	34kb (resection)	YMV80
IMA5 Fwd	TTCTCCGTCTAGGCATTTACG	IMA5 (loading control)	YMV80
IMA5 Rev	ATCGGTTCTTCTGGCAAGTTG	IMA5 (loading control)	YMV80

Supplemental Table 3. Other oligonucleotides used in this study

**Name**	**Sequence**
P1-CORE-Arg5,6	ATGTTAGGAATGCAATATTCCAAAATGCTGTCTGTGTCAG TGAGAATAACGAGCTCGTTTTCGACACTGG
P2-CORE-Arg5,6	GCCCTTATCTGGCGTGGTTATTACCATAGAAAAGTTTATTC AATGTTTTTTCCTTACCATTAAGTTGAT
Arg_Ya'HO_Arg check Fwd	TACCAGCCAACATCAGTGTAG
Arg_Ya'HO_Arg check Rev	TTTTCCCTTTGGGCTCTTCTC
P1 Arg5,6 HO inc	ATGTTAGGAATGCAATATTCCAAAATGCTGTCTGTGTCAG TGAGAATAACTTGATTTTTGTTCTTTCGGGG
P2 Arg5,6 HO inc	GCCCTTATCTGGCGTGGTTATTACCATAGAAAAGTTTATTC AATGTTTTTCGTGAAAGTATCCTAAAAGTACA
Arg_Ya'HO_Z1-Z2'_Arg check Fwd1	GGAGTCACACTCCCGTTAAAA
Arg_Ya'HO_Z1-Z2'_Arg check Rev2	CATTGGGAACAAGAGCAAGAG
CDC14 S3	CTACAAGCGCCGCCGGTGGTATAAGAAAAATAAGTGGCT CCATCAAGAAACGTACGCTGCAGGTCGAC
CDC14 S2	TAAGTTTTTTTATTATATGATATATATATATATAAAAATGA AATAAATTAATCGATGAATTCGAGCTCG
CDC14 (-215)	CATTATCCCAAAGTTTCGCGC
CDC14 (+233)	TATTCCTAGGTACCAGGTAGC
KanB	CTGCAGCGAGGAGCCGTAAT
MRE11-S3	GGACGCCAAAGACGGATATTCTTGGAAGTCTCCTTGCTAA GAAAAGAAAACGTACGCTGCAGGTCGAC
MRE11-S2	CCCTTGGTTATAAATAGGATATAATATAATATAGGGATCA AGTACAACTAATCGATGAATTCGAGCTCG
Mre11-265bp	CTCAACTGTCATATCCGATGC
Exo1 KO Fwd	ACCACATTAAAATAAAAGGAGCTCGAAAAAACTGAAAGG CGTAGAAAGGACAGCTGAAGCTTCGTACGC
Exo1 KO Rev	TTTTCATTTGAAAAATATACCTCCGATATGAAACGTGCAG TACTTAACTTGCATAGGCCACTAGTGGATCTG
Exo1 -113	GTCCTTGCTCCTTCAGGTATA
Exo1 +109	CTGTCCTACTTTACTGGGCAT
Dna2 (-1009)	ACTACTCGGTTGGATCCATAC

Dna2 (+522)	AAATTGCTGCAGAGTACGAGC
Dna2-S3	TCGTAAGCGATAAACCTATCATAAAGGAAATTCTACAAGA GTATGAAAGTCGTACGCTGCAGGTCGAC
Dna2-S2	GCTGTGATAGCTTTCCTGTTATGGAGAAGCTCTTCTTATTC CCCCTGTCAATCGATGAATTCGAGCTCG
Dna2 S17 to A Fwd	ATATCTGTAGCGCCTGCGAAGAAGACAGAGGAAAAAG
Dna2 S17 to A Rev	ACTCGCAGACCTCTTGTTCTTC
Dna2 S237 to A Fwd	TTGCCATCAGCGCCCATCAAGGCACCCAACGTTGAAAAAA AAGC
Dna2 S237 to A Rev	ATCGCTGAATTTCGTTATCGTCGG
Dna2 +1356	AGAACTTTGTTCGCCTTTGCC
S17D Dna2 Fwd	ATATCTGTCGACCCTGCGAAGAAGACAGAGGAAAAAG
S237D Dna2 Fwd	TTGCCATCGGATCCCATCAAGGCACCCAACGTTGAAAAAA AAGC
Dna2 Fwd (+3)	CCCGGAACGCCACAGAAGAAC
Dna2 Rev (-1013)	GGATCCACTAGTTCTAGAGCG
Pol12 S3	TTTATCTACACAACGTGTGGAAGCGCGCTAGAGTTGACTT GATTGCTAGTCGTACGCTGCAGGTCGAC
Pol12 S2	TGGTGCTACCTTGAGCTATTCCATTAGTTAAGTTTGAATTA AATATATCAATCGATGAATTCGAGCTCG
Pol12 (-242) fwd	GGTTCTCGCCTGACATAATGA
DNA2 (+858) Rev	CTCACTCTTGCGTTTCTCAAC
DNA2 terminator Fwd	TGACAGGGGGAATAAGAAGAG
Mata Distal 2 (CCG46)	AGGATGCCCTTGTTTTGTTTACTG
Arg_Ya'HO_Arg check Fwd	TACCAGCCAACATCAGTGTAG
Act1 for KpnI cut Fwd	CAGGTATTGCCGAAAGAATGC
Act1 for KpnI cut Rev	GTCCCTGAGATGAGTAAGATC
Act1 Probe 2	CTTGTGGTGAACGATAGATGG
-1.5kb HO Fwd	GTCTGTGATGTTGGAGATATG
-1.5kb HO Rev	AGAATTAGCGGACCTCTTGAG
FUN30 KO Fwd	GTAAGGAACGTAAACAAGAAAAAGAGAGAAAATACGCTA TAGTTGAAAACCAGCTGAAGCTTCGTACGC
FUN30 KO Rev	TGGTTTATTTTCTGCTTATCTATTTACTTTTTTACTATATTT TTATTTATGCATAGGCCACTAGTGGATCTG
Fun30 Fwd (-238)	TTATCAAGGCTCAAACGGCTG
Fun30 Rev (+241)	AAGTTCACCATTTCTCTCCCC
SGS1 KO Rev	TTGGCGAATGGTGTCGTAGTTATAAGTAACACTATTTATTT TTCTACTCTGCATAGGCCACTAGTGGATCTG
SGS1 KO Fwd	ATTATTGTTGTATATATTTAAAAAATCATACACGTACACA CAAGGCGGTACAGCTGAAGCTTCGTACGC

Sgs1-154	CTGGGTGATCATTGGTGATAC
Sgs1+177	TGCACACCACAATATGTCGTG
