# Supplementary material for: Cdc14 phosphatase counteracts Cdk-dependent Dna2 phosphorylation to inhibit resection during recombinational DNA repair
Source: Nat Commun. 2023 May 12;14:2738. doi: 10.1038/s41467-023-38417-5 (PMC10182099; doi:10.1038/s41467-023-38417-5)
Supplement: Supplementary file 3 — Description of Additional Supplementary Files [file 41467_2023_38417_MOESM3_ESM.pdf]

Description of Additional Supplementary Files

File Name: Supplementary Data 1
Description: A fasta file containing the PMV reference genome used for the genome wide sequencing alignments.
