# Supplementary material for: Cdc14 phosphatase counteracts Cdk-dependent Dna2 phosphorylation to inhibit resection during recombinational DNA repair
Source: Nat Commun. 2023 May 12;14:2738. doi: 10.1038/s41467-023-38417-5 (PMC10182099; doi:10.1038/s41467-023-38417-5)
Supplement: Supplementary file 5 — Reporting Summary [file 41467_2023_38417_MOESM5_ESM.pdf]

nature portfolio

Corresponding author(s): Andrés Clemente Blanco

Last updated by author(s): Apr 13, 2023

Reporting Summary

Nature Portfolio wishes to improve the reproducibility of the work that we publish. This form provides structure for consistency and transparency in reporting. For further information on Nature Portfolio policies, see our Editorial Policies and the Editorial Policy Checklist.

Statistics

For all statistical analyses, confirm that the following items are present in the figure legend, table legend, main text, or Methods section.

n/a	Confirmed	
☐	☒	The exact sample size (*n*) for each experimental group/condition, given as a discrete number and unit of measurement
☐	☒	A statement on whether measurements were taken from distinct samples or whether the same sample was measured repeatedly
☐	☒	The statistical test(s) used AND whether they are one- or two-sided<br>*Only common tests should be described solely by name; describe more complex techniques in the Methods section.*
☒	☐	A description of all covariates tested
☐	☒	A description of any assumptions or corrections, such as tests of normality and adjustment for multiple comparisons
☐	☒	A full description of the statistical parameters including central tendency (e.g. means) or other basic estimates (e.g. regression coefficient) AND variation (e.g. standard deviation) or associated estimates of uncertainty (e.g. confidence intervals)
☐	☒	For null hypothesis testing, the test statistic (e.g. *F*, *t*, *r*) with confidence intervals, effect sizes, degrees of freedom and *P* value noted<br>*Give P values as exact values whenever suitable.*
☒	☐	For Bayesian analysis, information on the choice of priors and Markov chain Monte Carlo settings
☒	☐	For hierarchical and complex designs, identification of the appropriate level for tests and full reporting of outcomes
☒	☐	Estimates of effect sizes (e.g. Cohen's *d*, Pearson's *r*), indicating how they were calculated

Our web collection on statistics for biologists contains articles on many of the points above.

Software and code

Policy information about availability of computer code

Data collection	No data collection software was used
Data analysis	Sequence reads were aligned to the S. cerevisiae reference genome by using Bowtie v1.0.0. Discordant read analysis was performed by aligning reads with Bowtie2 v2.4.4. Sam files were converted to bam files by using Samtools v1.12. Bedgraphs files were used to calculate the coverage along the chromosomes by using Bedtools, v2.30.0. The Deeptools, v3.5.1 utility utility was used for the alignment and normalization of the bam data. For visualization of the BedGraph files we used the IGB v9.0.2 utility. FIJI, v1.0 was used for the processing and analysis of the images. FACS samples were proccessed by using the CellQuest Pro v6.0 software.

For manuscripts utilizing custom algorithms or software that are central to the research but not yet described in published literature, software must be made available to editors and reviewers. We strongly encourage code deposition in a community repository (e.g. GitHub). See the Nature Portfolio guidelines for submitting code & software for further information.

Data

Policy information about availability of data

All manuscripts must include a data availability statement. This statement should provide the following information, where applicable:

- Accession codes, unique identifiers, or web links for publicly available datasets
- A description of any restrictions on data availability
- For clinical datasets or third party data, please ensure that the statement adheres to our policy

Genomic datasets for the wild-type strain are available at the Sequence Read Archive (SRA) repository (https://www.ncbi.nlm.nih.gov/sra/PRJNA785778). Genomic datasets for the cdc14-1 mutant are available at the SRA repository at the link: https://www.ncbi.nlm.nih.gov/sra/PRJNA877059, with the accession number: PRJNA877059.

Human research participants

Policy information about studies involving human research participants and Sex and Gender in Research.

Reporting on sex and gender	No human participants were used in this study.
Population characteristics	No human participants were used in this study.
Recruitment	No human participants were used in this study.
Ethics oversight	No human participants were used in this study.

Note that full information on the approval of the study protocol must also be provided in the manuscript.

Field-specific reporting

Please select the one below that is the best fit for your research. If you are not sure, read the appropriate sections before making your selection.

☒ Life sciences ☐ Behavioural & social sciences ☐ Ecological, evolutionary & environmental sciences

For a reference copy of the document with all sections, see nature.com/documents/nr-reporting-summary-flat.pdf

Life sciences study design

All studies must disclose on these points even when the disclosure is negative.

Sample size	For DNA extactions, 10 ml of an OD600=0.4 were taken. For protein extractions, 5ml of an OD600=0.4 were taken.
Data exclusions	No data were excluded from the analysis.
Replication	Three bilogical replications were performed for the experiments designed to identify DNA repair problems in the absence of Cdc14 activity and for the analysis of DNA repair in the presence of alpha factor or nocodazole. For the rest of the experimetns, two biological replication were performed for each experiments. All attemps at replication were successful.
Randomization	This is not relevant to our study, since no human partipants were involved.
Blinding	This is not relevant to our study, since no human partipants were involved.

Reporting for specific materials, systems and methods

We require information from authors about some types of materials, experimental systems and methods used in many studies. Here, indicate whether each material, system or method listed is relevant to your study. If you are not sure if a list item applies to your research, read the appropriate section before selecting a response.

Materials & experimental systems

n/a	Involved in the study	
☐	☒	Antibodies
☒	☐	Eukaryotic cell lines
☒	☐	Palaeontology and archaeology
☒	☐	Animals and other organisms
☒	☐	Clinical data
☒	☐	Dual use research of concern

Methods

n/a	Involved in the study	
☒	☐	ChIP-seq
☐	☒	Flow cytometry
☒	☐	MRI-based neuroimaging

Antibodies

Antibodies used

Antifluoresceine-AP Fab fragments, Merk, 11426338910 (1:250000 dilution)
Anti-Rad53, AbCam, ab104232 (1:1000 dilution)
Anti-HA, Merk, 11666606001 (1:2500 dilution)
Anti-MYC, Merk, C3956 (1:2500 dilution)
Secondary anti-rabbit antibody, GE Healthcare, NA934 (1:5000 dilution)
Secondary anti-mouse antibody, GE Healthcare, NA931 (1:25000 dilution)

Validation

All antibodies have been validated following the information provided by the manufacturer:
Antifluoresceine-AP Fab fragments: https://www.sigmaaldrich.com/ES/es/product/roche/11426338910
Anti-Rad53: https://www.abcam.com/rad53-antibody-ab104232.html
Anti-HA: https://www.sigmaaldrich.com/ES/es/product/roche/roaha
Anti-MYC: https://www.sigmaaldrich.com/ES/es/product/sigma/c3956

Flow Cytometry

Plots

Confirm that:

☒ The axis labels state the marker and fluorochrome used (e.g. CD4-FITC).

☒ The axis scales are clearly visible. Include numbers along axes only for bottom left plot of group (a 'group' is an analysis of identical markers).

☒ All plots are contour plots with outliers or pseudocolor plots.

☒ A numerical value for number of cells or percentage (with statistics) is provided.

Methodology

Sample preparation

Cells were fixed with 70% ethanol, treated with proteinase K, sonicated and stained with propidium iodide.

Instrument

BD FACScalibur flow cytometer

Software

CellQuest Pro v6.0

Cell population abundance

10.000 cells

Gating strategy

Samples stained with propidium iodide were measured in the FL2-H channel. No gating strategy was used. All collected events were included in the analysis.

☐ Tick this box to confirm that a figure exemplifying the gating strategy is provided in the Supplementary Information.
