# Supplementary figures and images for: Cdc14 phosphatase counteracts Cdk-dependent Dna2 phosphorylation to inhibit resection during recombinational DNA repair
Source: Nat Commun. 2023 May 12;14:2738. doi: 10.1038/s41467-023-38417-5 (PMC10182099; doi:10.1038/s41467-023-38417-5)

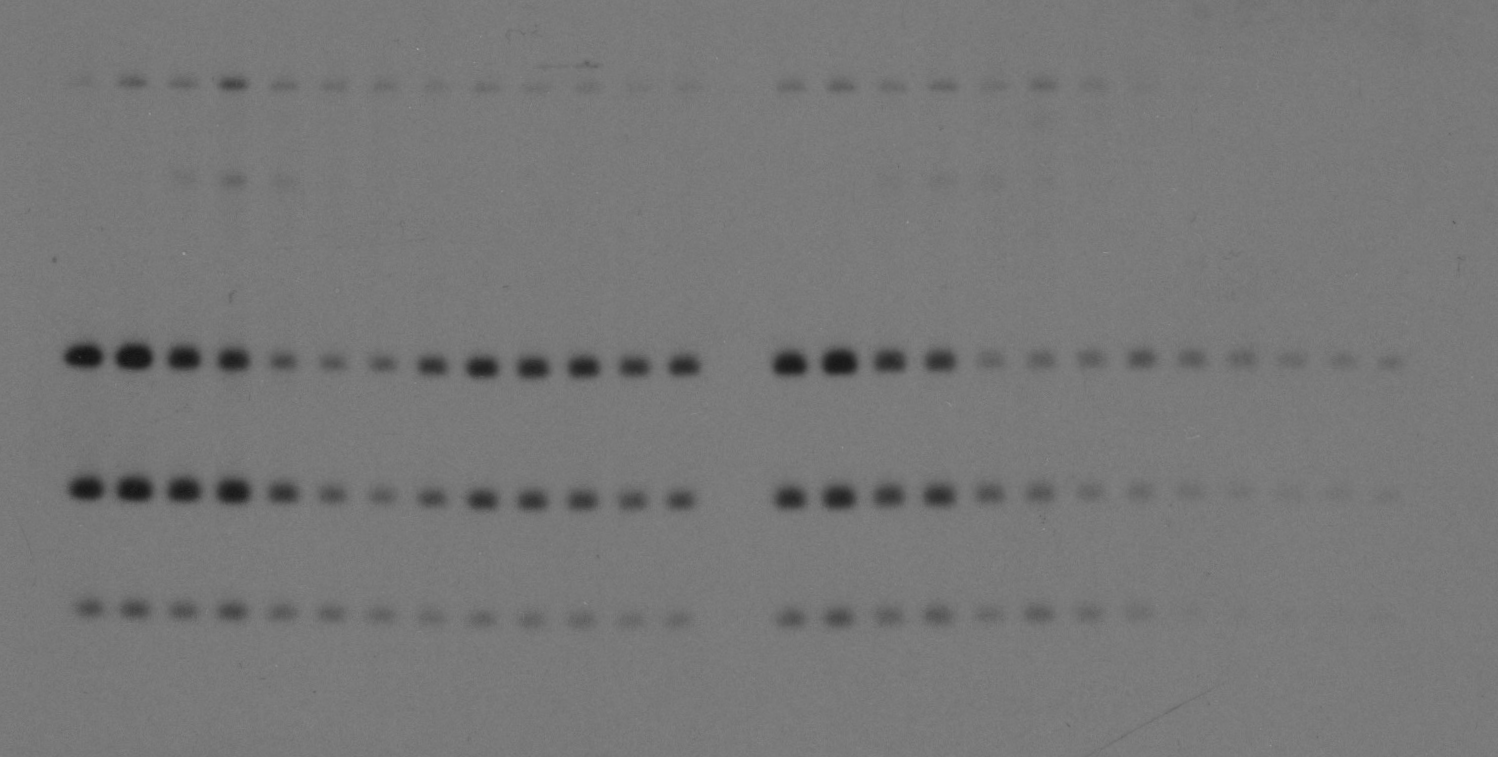

Supplement: Supplementary file 6 — Source Data [file 41467_2023_38417_MOESM6_ESM.zip › Source Data/Uncropped Blots/Figure 4B/3 kb, 6 kb, 21 kb.tif]

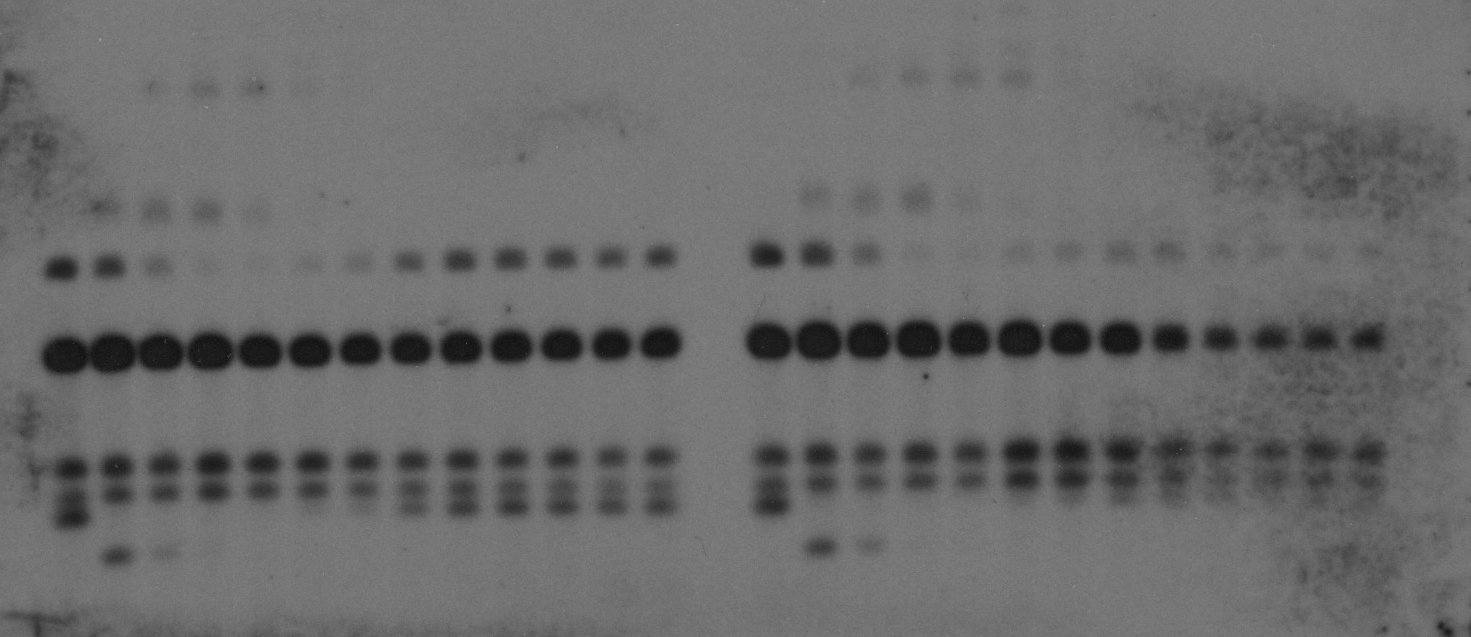

Supplement: Supplementary file 6 — Source Data [file 41467_2023_38417_MOESM6_ESM.zip › Source Data/Uncropped Blots/Figure 4B/0.7 kb, 27 kb, Act1, Mat.tif]

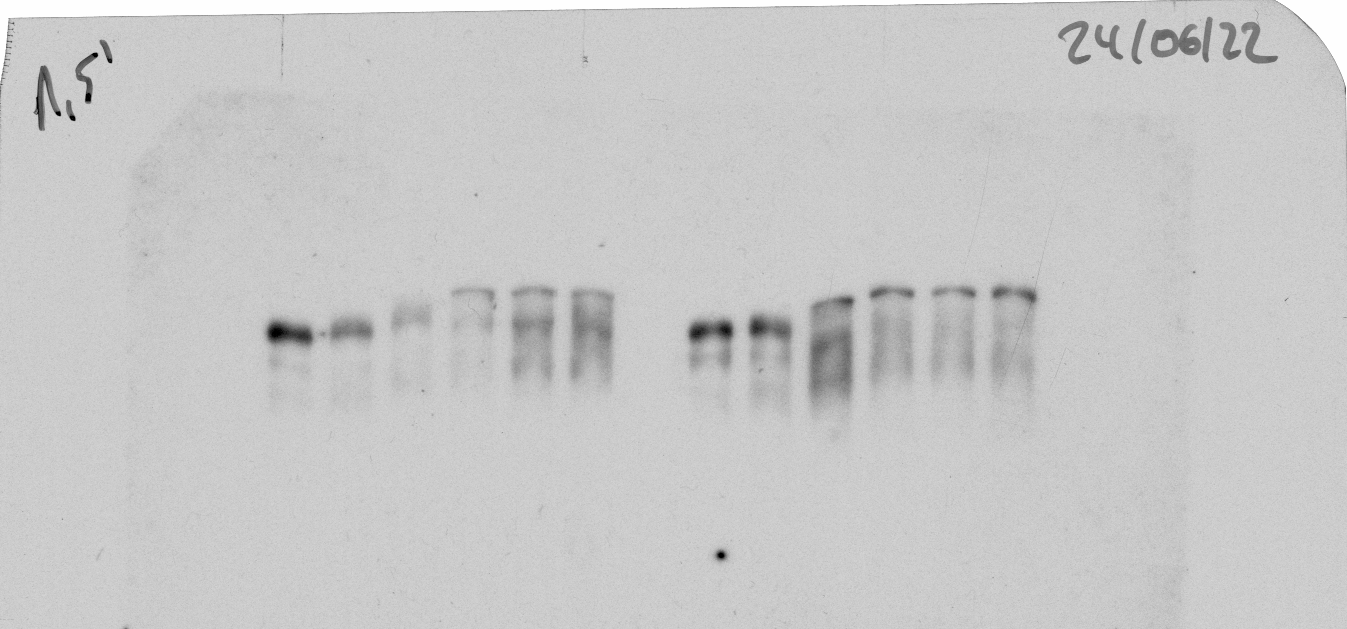

Supplement: Supplementary file 6 — Source Data [file 41467_2023_38417_MOESM6_ESM.zip › Source Data/Uncropped Blots/Figure 7D/Dna2-HA aF-aF 1.tiff]

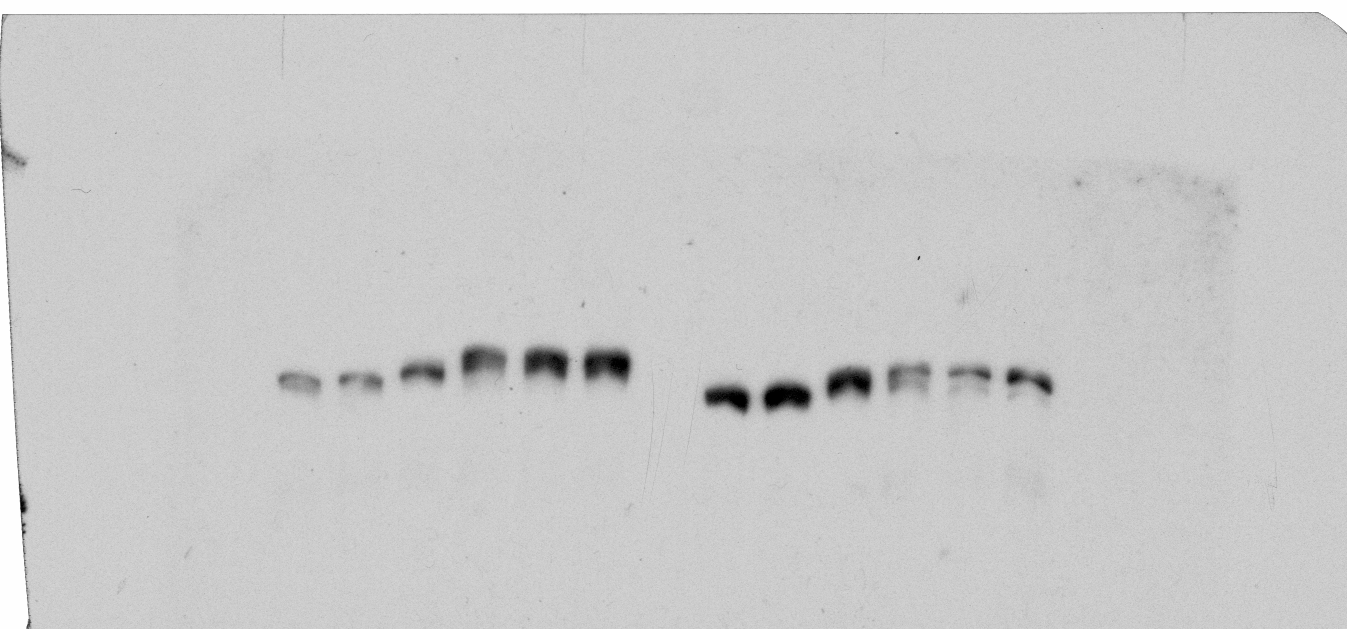

Supplement: Supplementary file 6 — Source Data [file 41467_2023_38417_MOESM6_ESM.zip › Source Data/Uncropped Blots/Figure 7D/Dna2-HA aF-Nz.tiff]

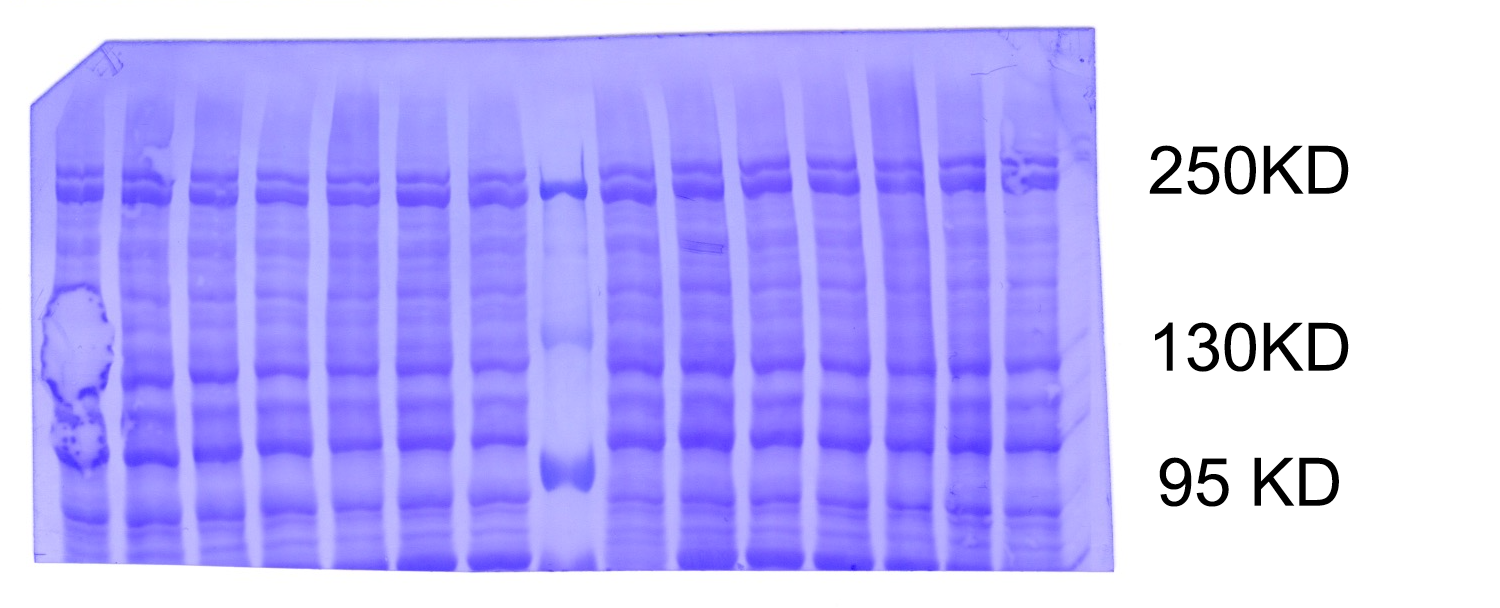

Supplement: Supplementary file 6 — Source Data [file 41467_2023_38417_MOESM6_ESM.zip › Source Data/Uncropped Blots/Figure 7C/Coomassie membrane_Rad53.tif]

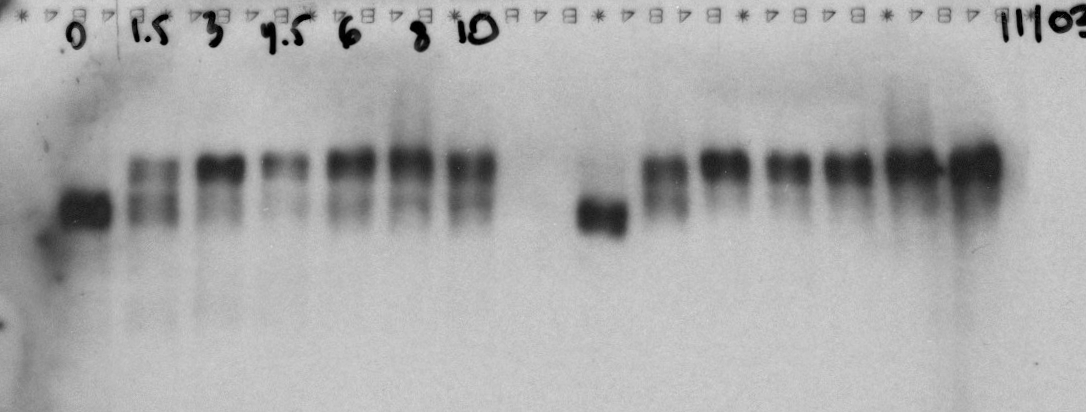

Supplement: Supplementary file 6 — Source Data [file 41467_2023_38417_MOESM6_ESM.zip › Source Data/Uncropped Blots/Figure 7C/Dna2-HA.tif]

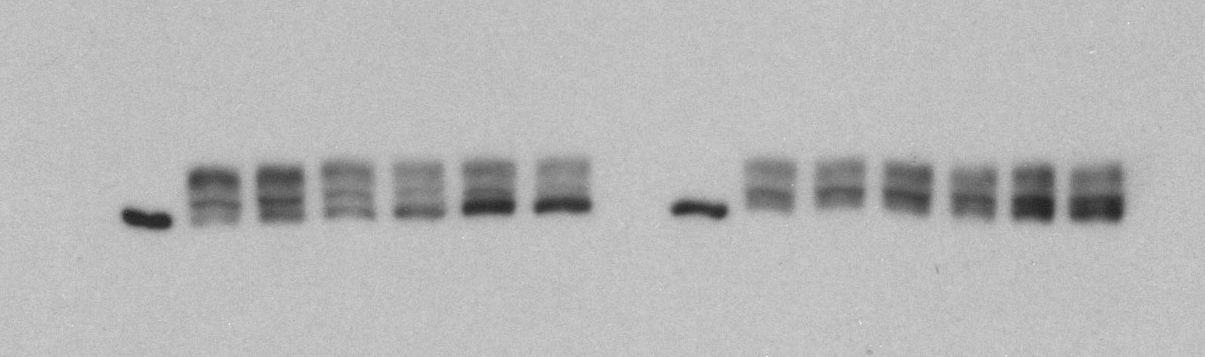

Supplement: Supplementary file 6 — Source Data [file 41467_2023_38417_MOESM6_ESM.zip › Source Data/Uncropped Blots/Figure 7C/Rad53.tif]

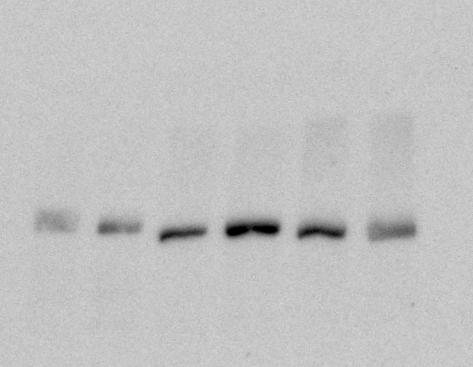

Supplement: Supplementary file 6 — Source Data [file 41467_2023_38417_MOESM6_ESM.zip › Source Data/Uncropped Blots/Figure 7B/Dna2-HA.tif]

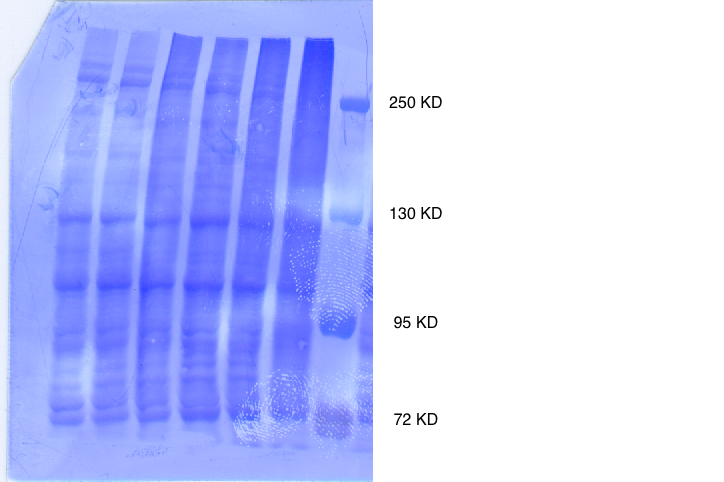

Supplement: Supplementary file 6 — Source Data [file 41467_2023_38417_MOESM6_ESM.zip › Source Data/Uncropped Blots/Figure 7B/Dna2 commassie 2.tiff]

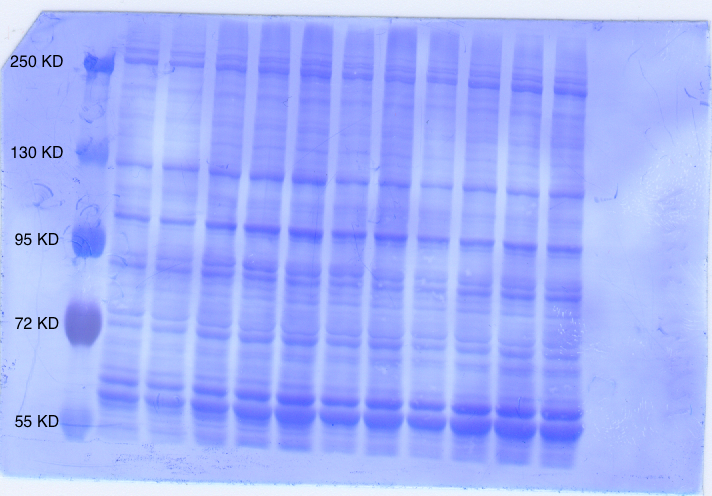

Supplement: Supplementary file 6 — Source Data [file 41467_2023_38417_MOESM6_ESM.zip › Source Data/Uncropped Blots/Figure 7B/Pol12 commassie.tiff]

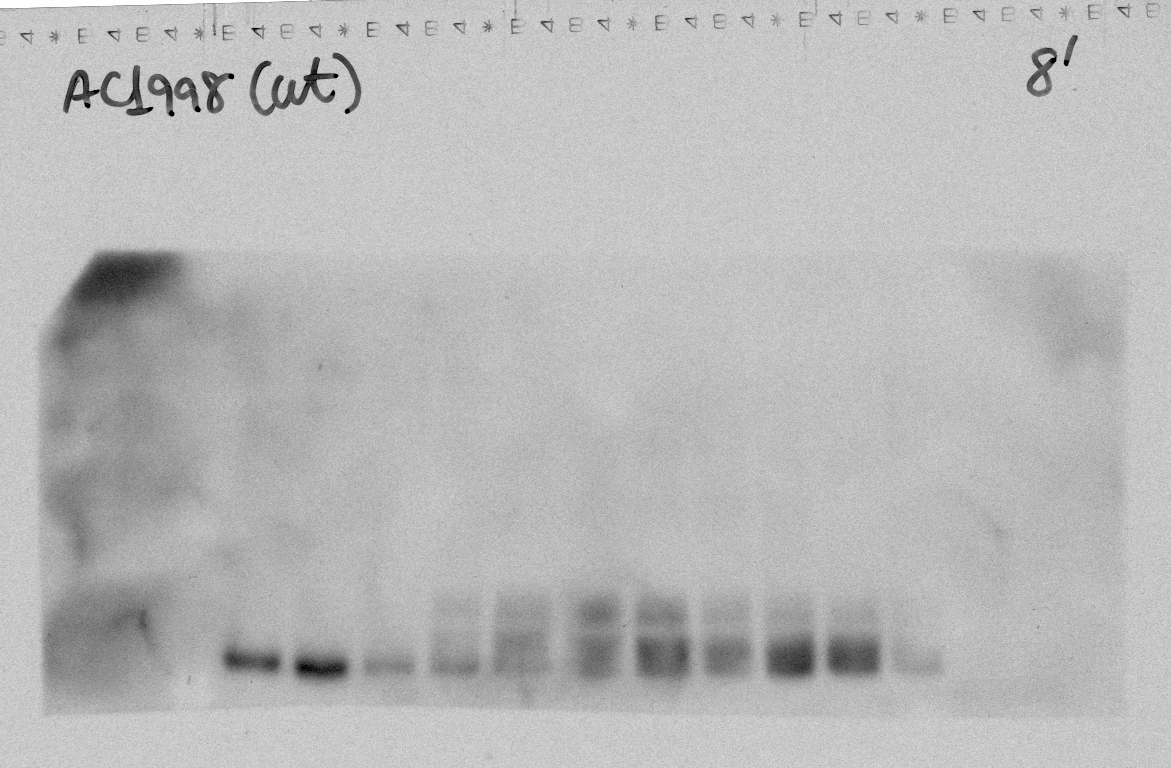

Supplement: Supplementary file 6 — Source Data [file 41467_2023_38417_MOESM6_ESM.zip › Source Data/Uncropped Blots/Figure 7B/Rad53.tiff]

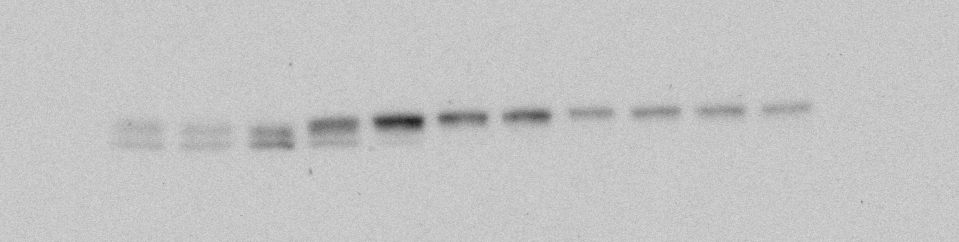

Supplement: Supplementary file 6 — Source Data [file 41467_2023_38417_MOESM6_ESM.zip › Source Data/Uncropped Blots/Figure 7B/Pol12-HA.tiff]

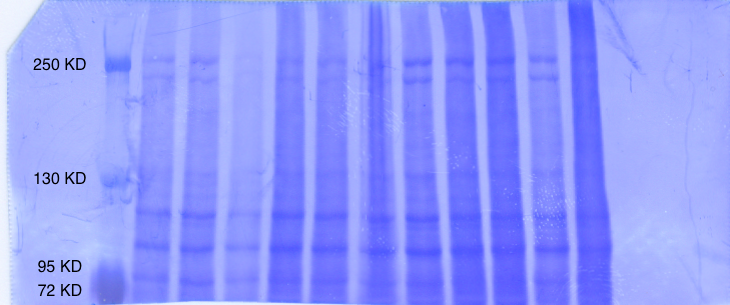

Supplement: Supplementary file 6 — Source Data [file 41467_2023_38417_MOESM6_ESM.zip › Source Data/Uncropped Blots/Figure 7B/Rad53 commassie.tiff]

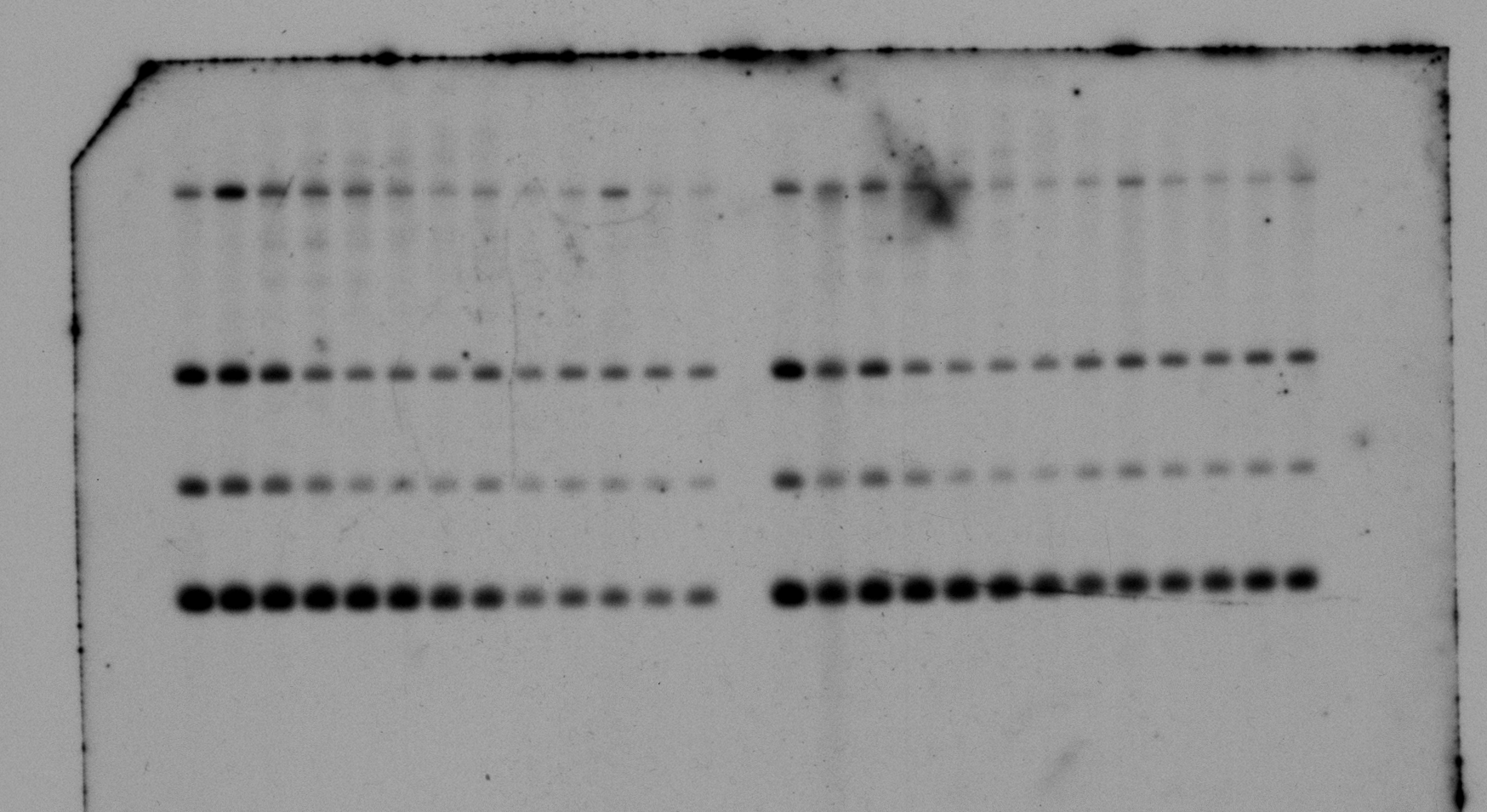

Supplement: Supplementary file 6 — Source Data [file 41467_2023_38417_MOESM6_ESM.zip › Source Data/Uncropped Blots/Figure 4C/3 kb, 6 kb, 21 kb.tif]

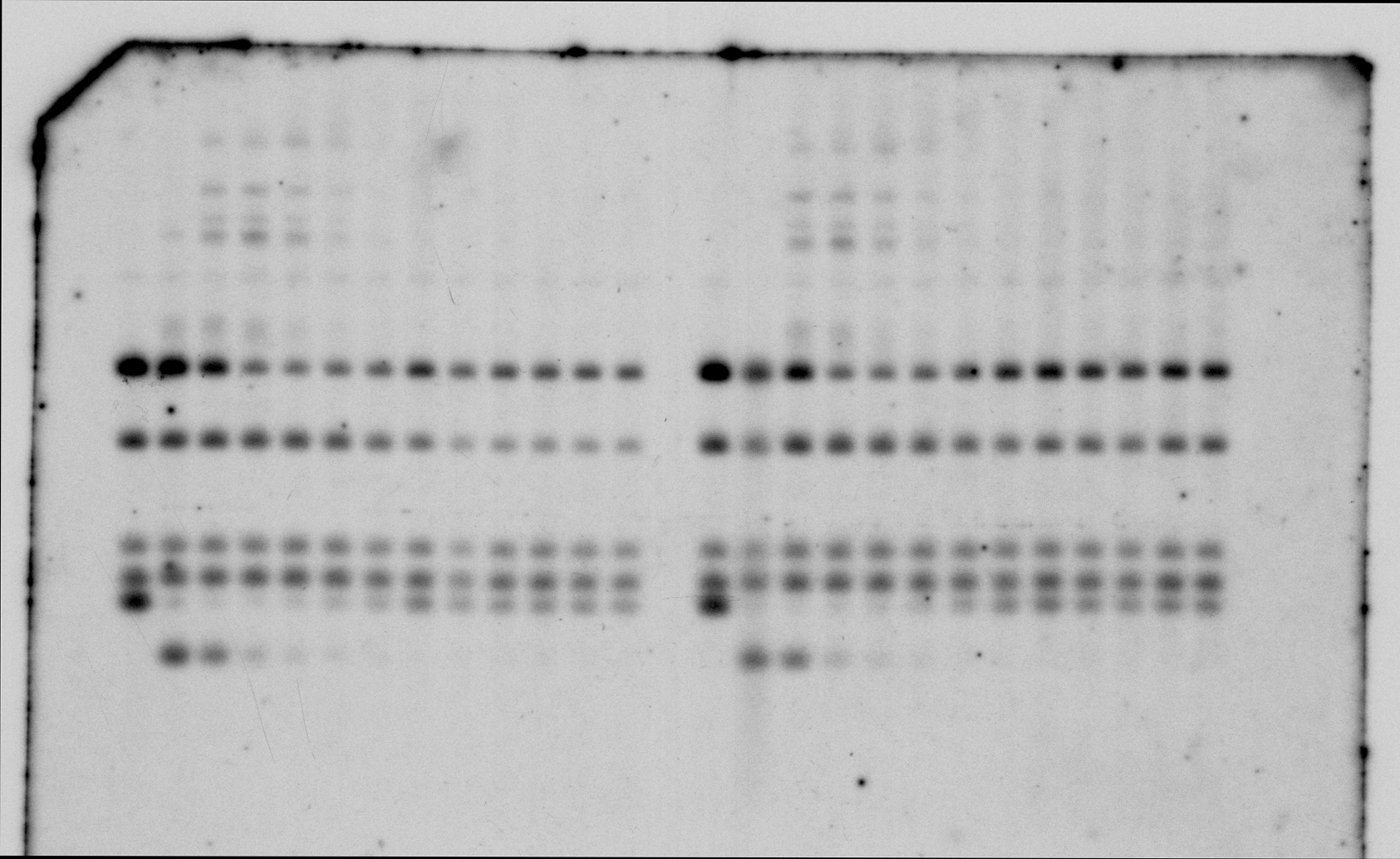

Supplement: Supplementary file 6 — Source Data [file 41467_2023_38417_MOESM6_ESM.zip › Source Data/Uncropped Blots/Figure 4C/0.7 kb, 27 kb, Act1, Mat.tiff]

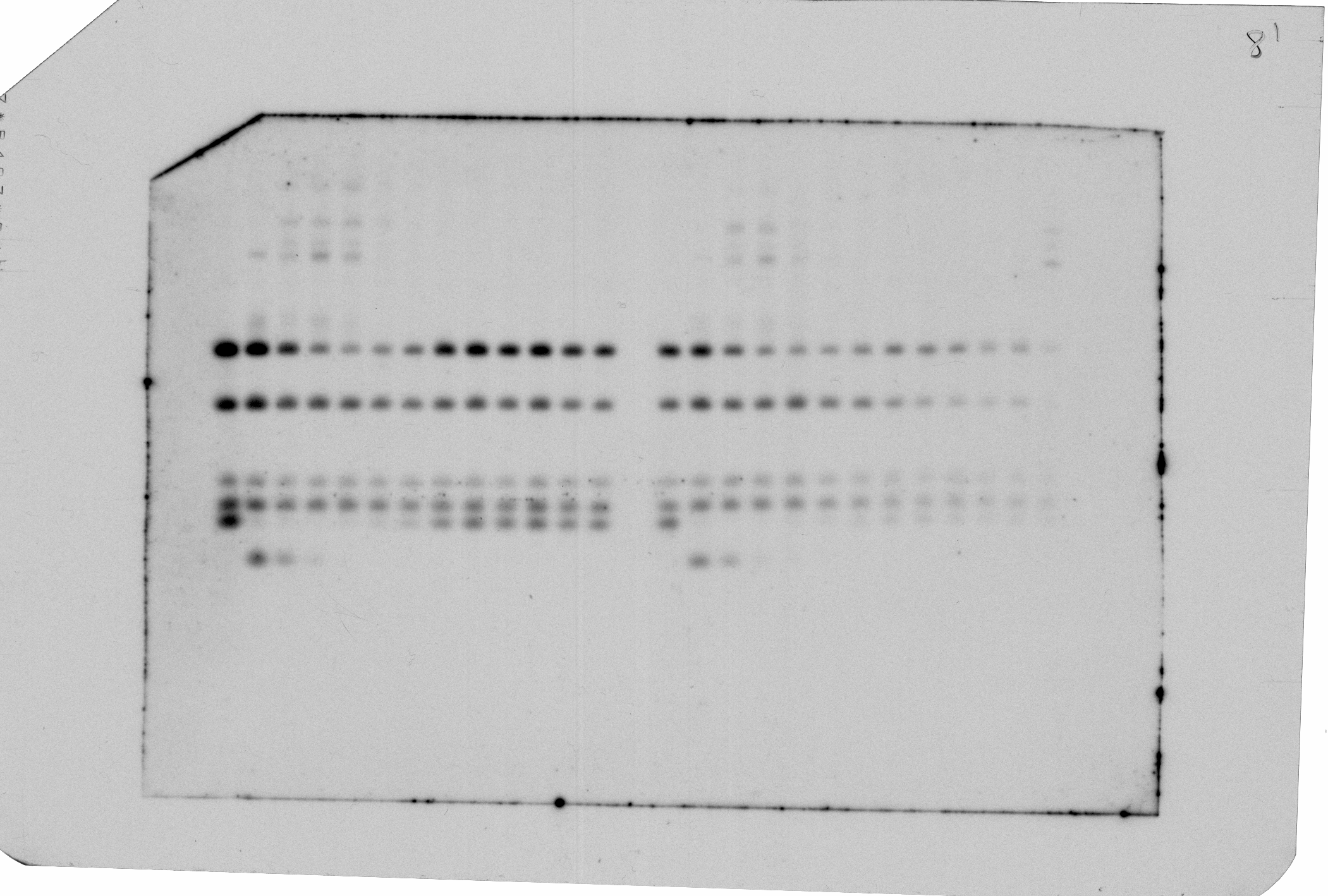

Supplement: Supplementary file 6 — Source Data [file 41467_2023_38417_MOESM6_ESM.zip › Source Data/Uncropped Blots/Supplemental Figure 3A/0.7 kb, 27 kb, Act1, Mat.tiff]

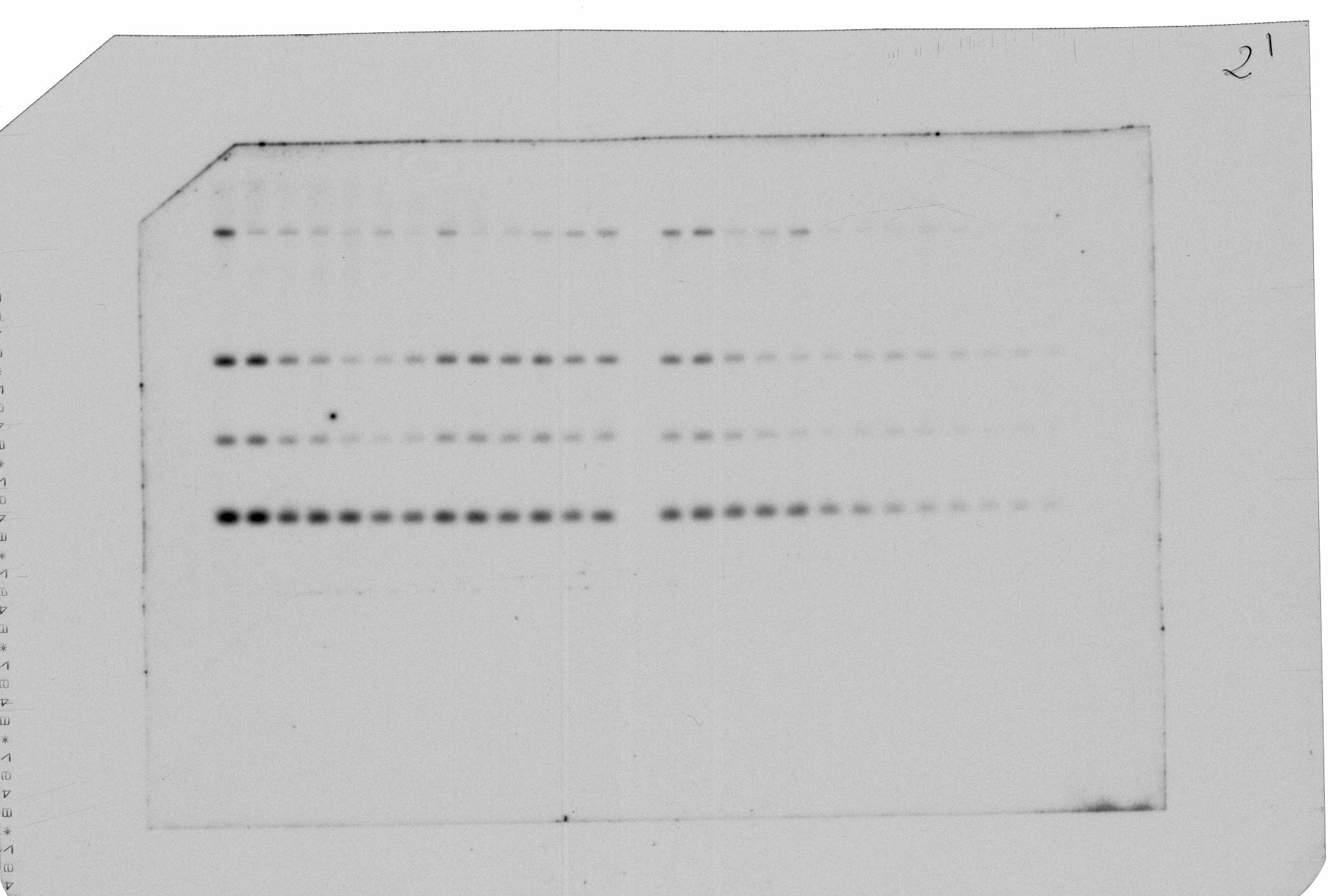

Supplement: Supplementary file 6 — Source Data [file 41467_2023_38417_MOESM6_ESM.zip › Source Data/Uncropped Blots/Supplemental Figure 3A/3 kb, 6 kb, 21 kb.tiff]

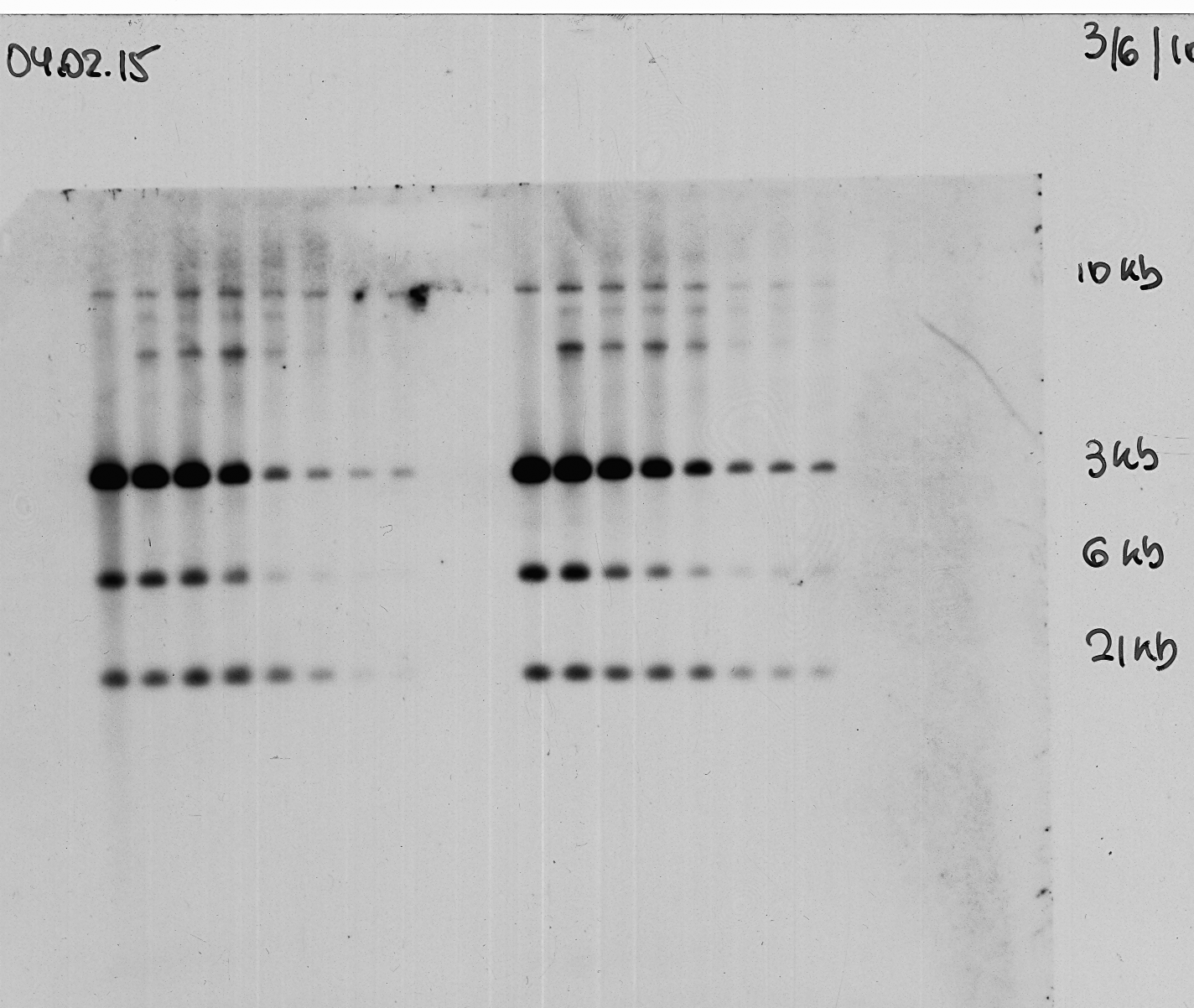

Supplement: Supplementary file 6 — Source Data [file 41467_2023_38417_MOESM6_ESM.zip › Source Data/Uncropped Blots/Supplemental Figure 1D/3 kb, 6 kb, 21 kb.tif]

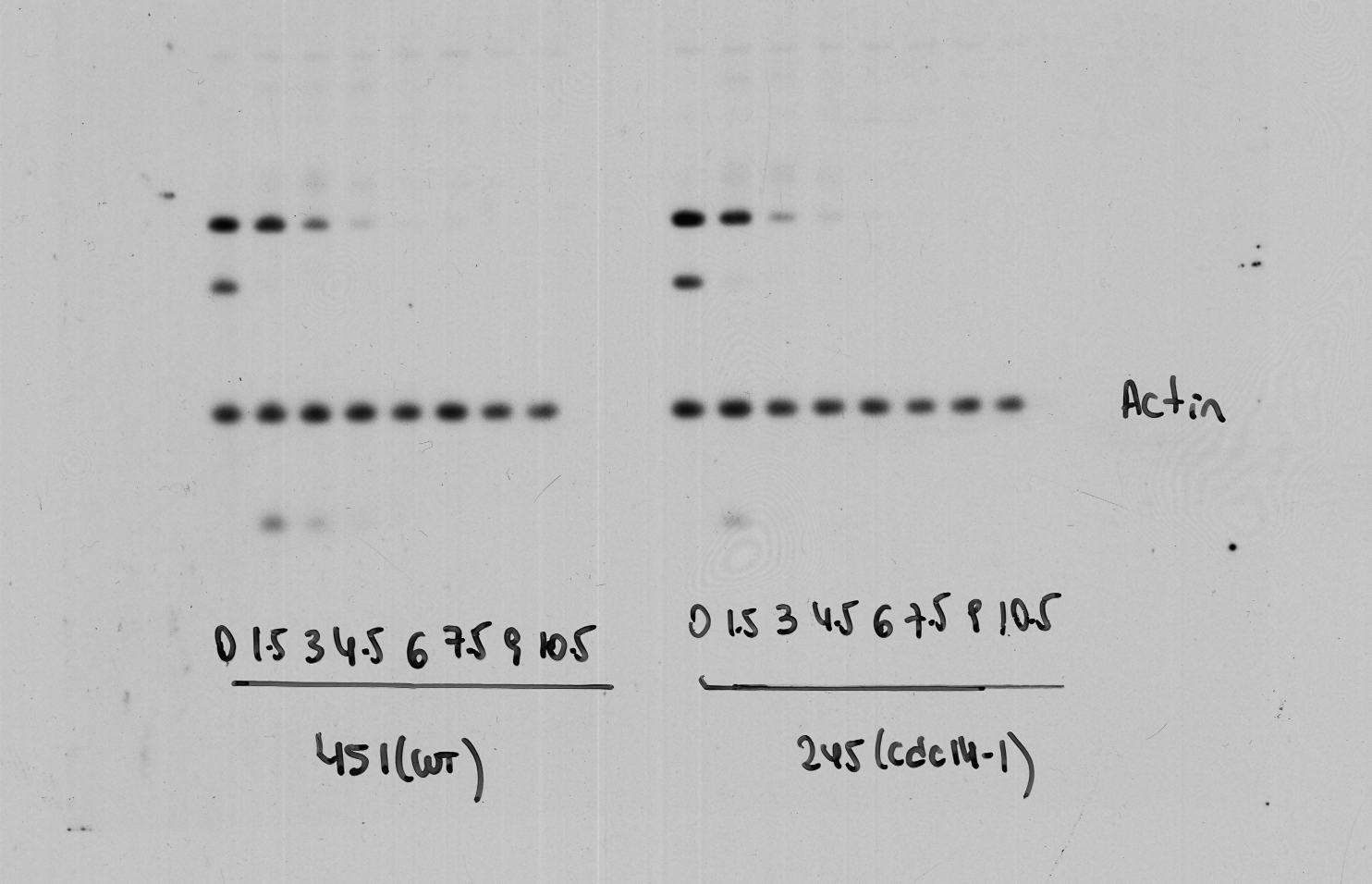

Supplement: Supplementary file 6 — Source Data [file 41467_2023_38417_MOESM6_ESM.zip › Source Data/Uncropped Blots/Supplemental Figure 1D/0,7 kb, actin and mat .tif]

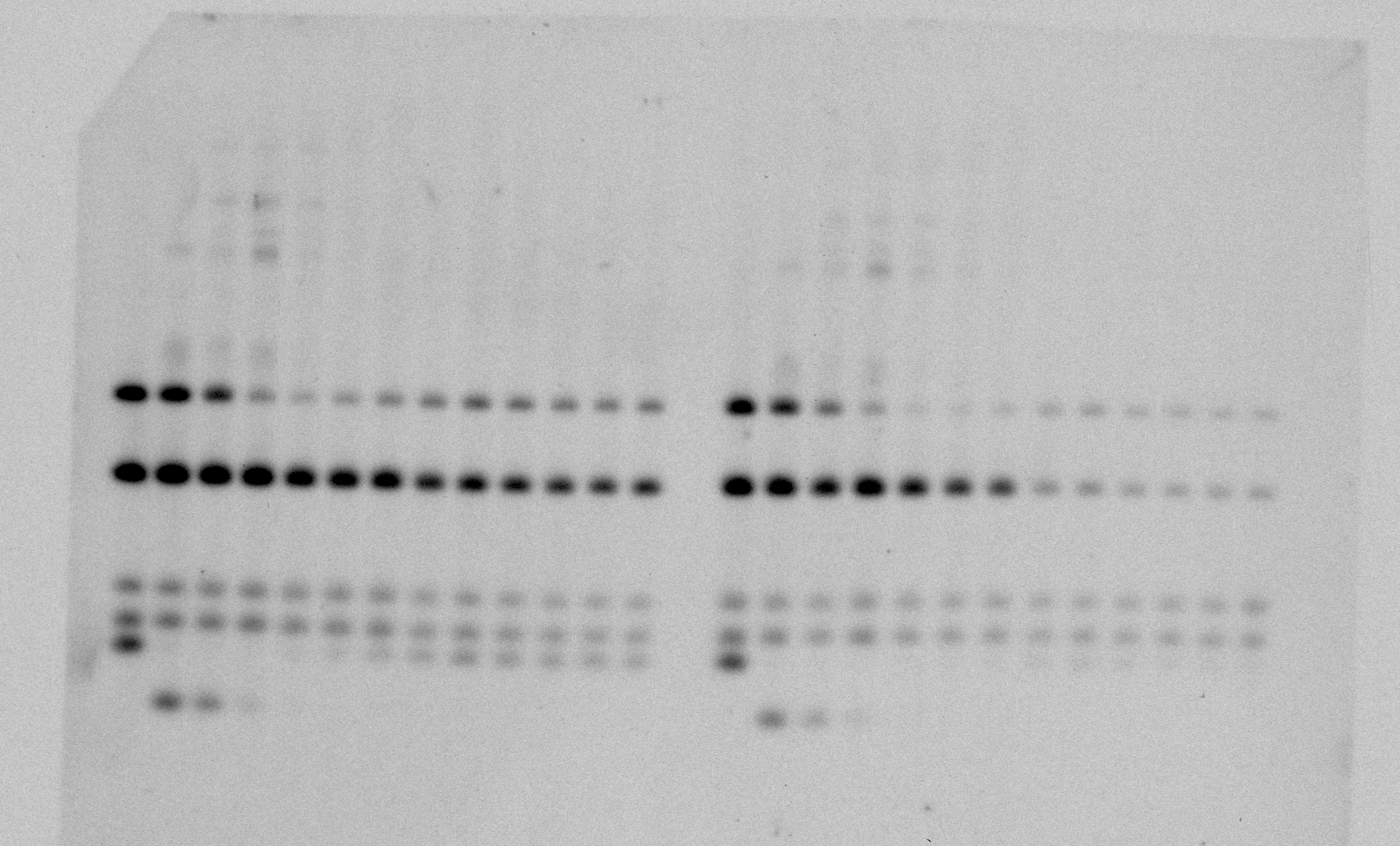

Supplement: Supplementary file 6 — Source Data [file 41467_2023_38417_MOESM6_ESM.zip › Source Data/Uncropped Blots/Figure 5A/0.7 kb, 27 kb, Act1, Mat.tiff]

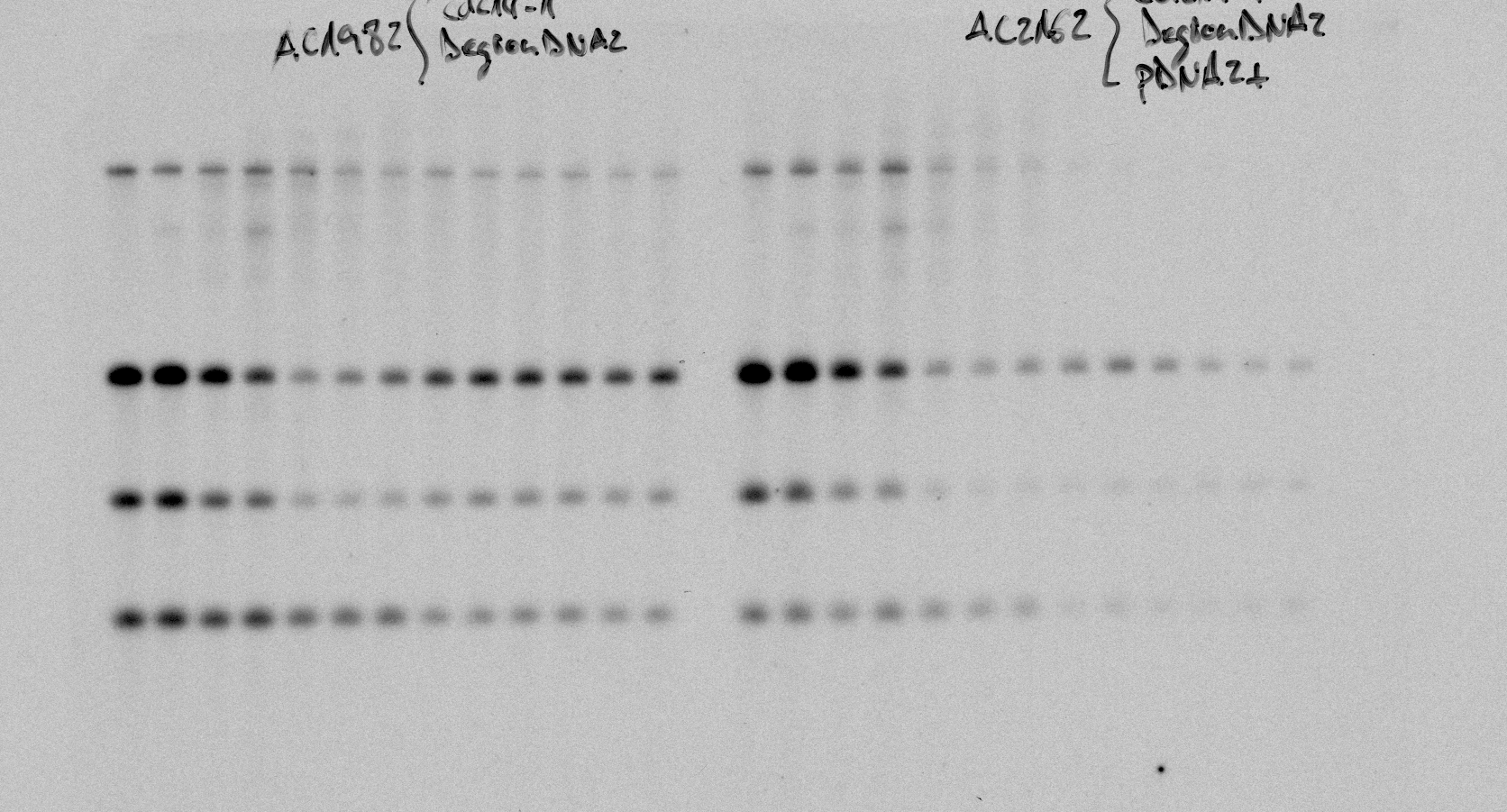

Supplement: Supplementary file 6 — Source Data [file 41467_2023_38417_MOESM6_ESM.zip › Source Data/Uncropped Blots/Figure 5A/3 kb, 6 kb, 21 kb.tiff]

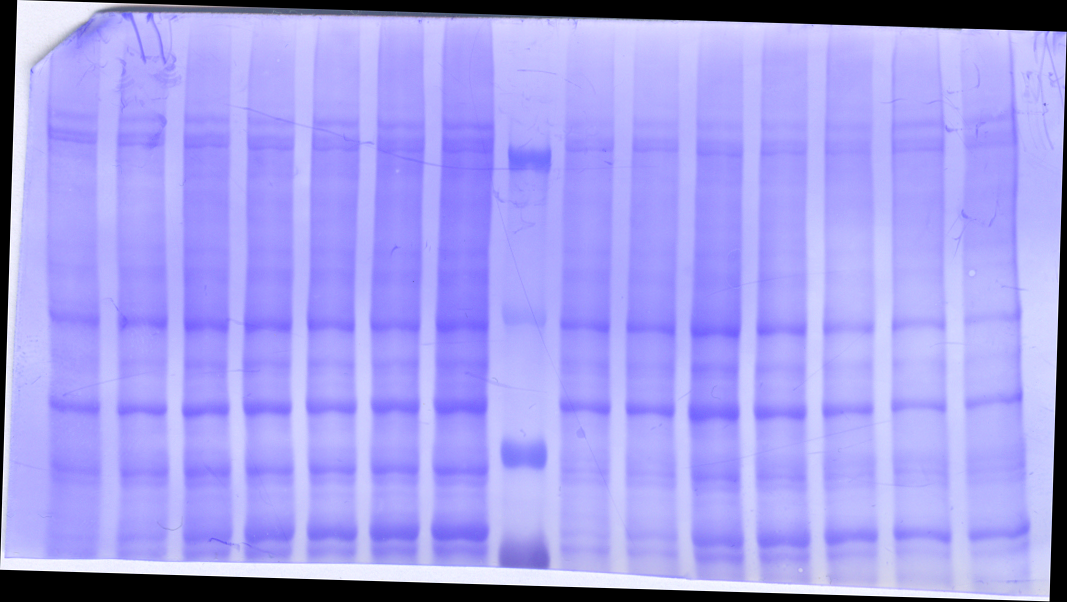

Supplement: Supplementary file 6 — Source Data [file 41467_2023_38417_MOESM6_ESM.zip › Source Data/Uncropped Blots/Supplemental Figure 1B/Coomassie Rad53.tiff]

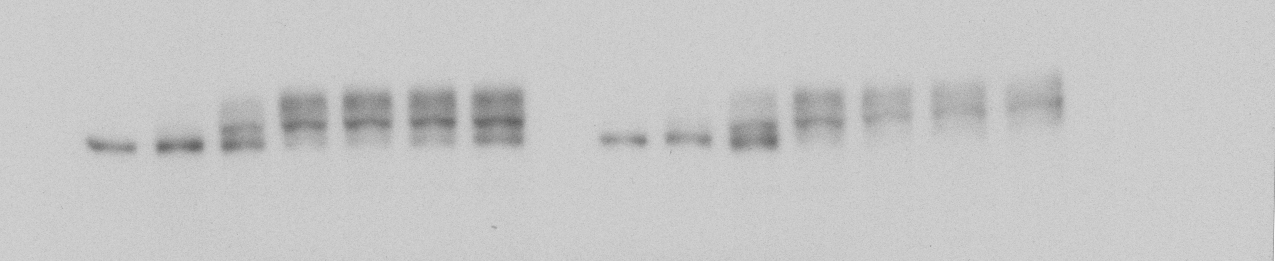

Supplement: Supplementary file 6 — Source Data [file 41467_2023_38417_MOESM6_ESM.zip › Source Data/Uncropped Blots/Supplemental Figure 1B/Rad53.tiff]

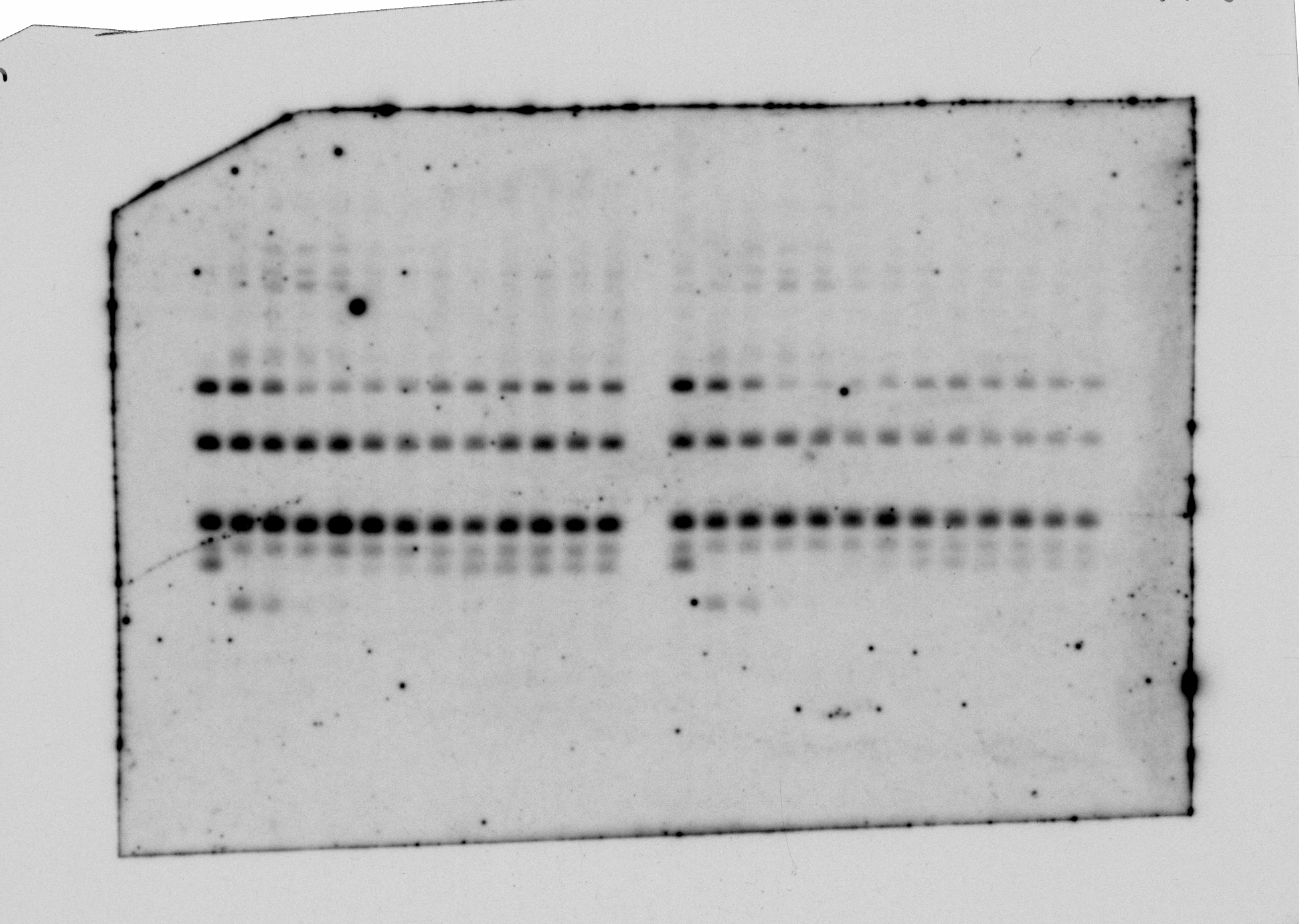

Supplement: Supplementary file 6 — Source Data [file 41467_2023_38417_MOESM6_ESM.zip › Source Data/Uncropped Blots/Supplemental Figure 5A/0.7 kb, 27 kb, Act1, Mat.tiff]

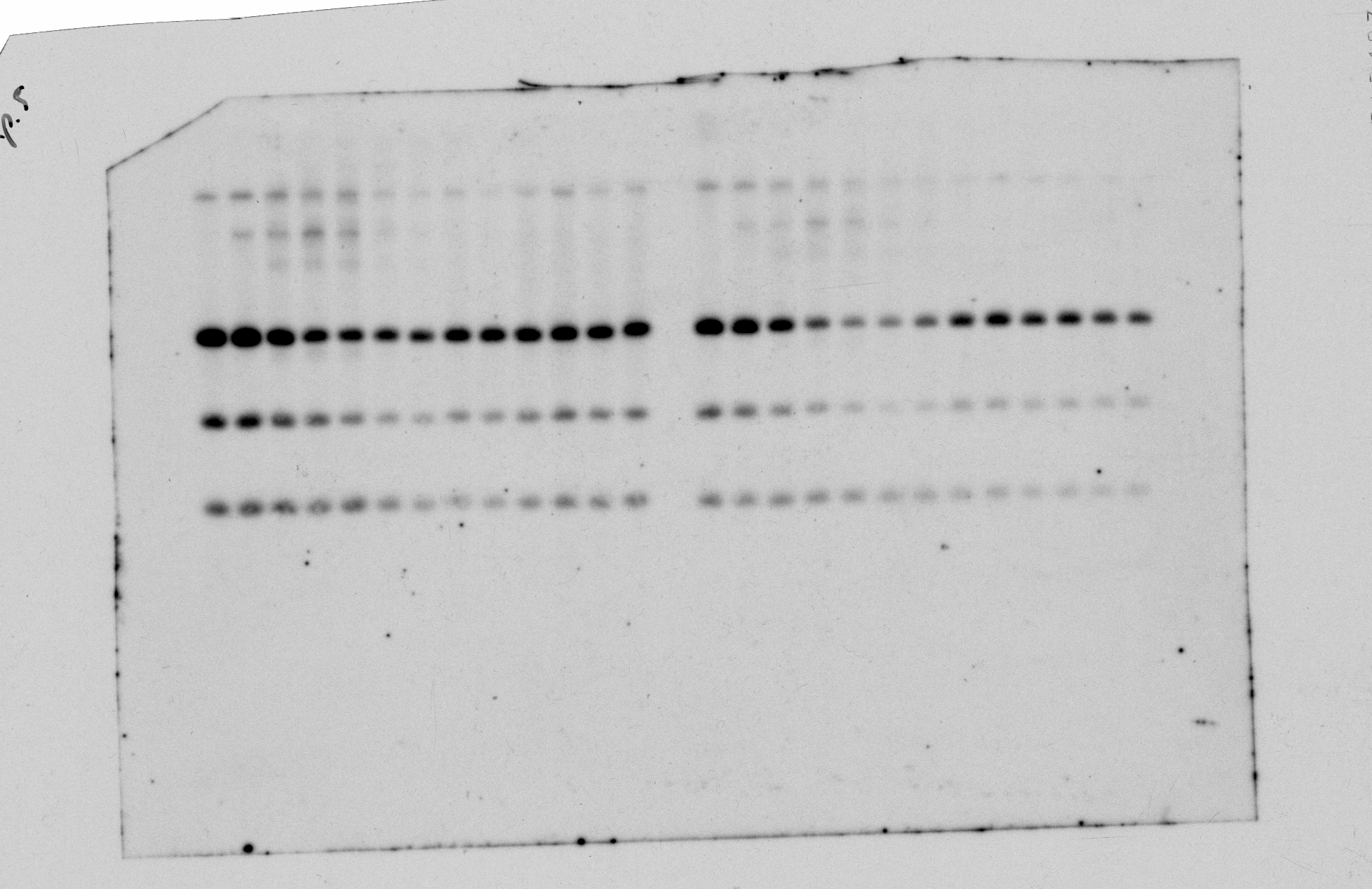

Supplement: Supplementary file 6 — Source Data [file 41467_2023_38417_MOESM6_ESM.zip › Source Data/Uncropped Blots/Supplemental Figure 5A/3 kb, 6 kb, 21 kb.tiff]

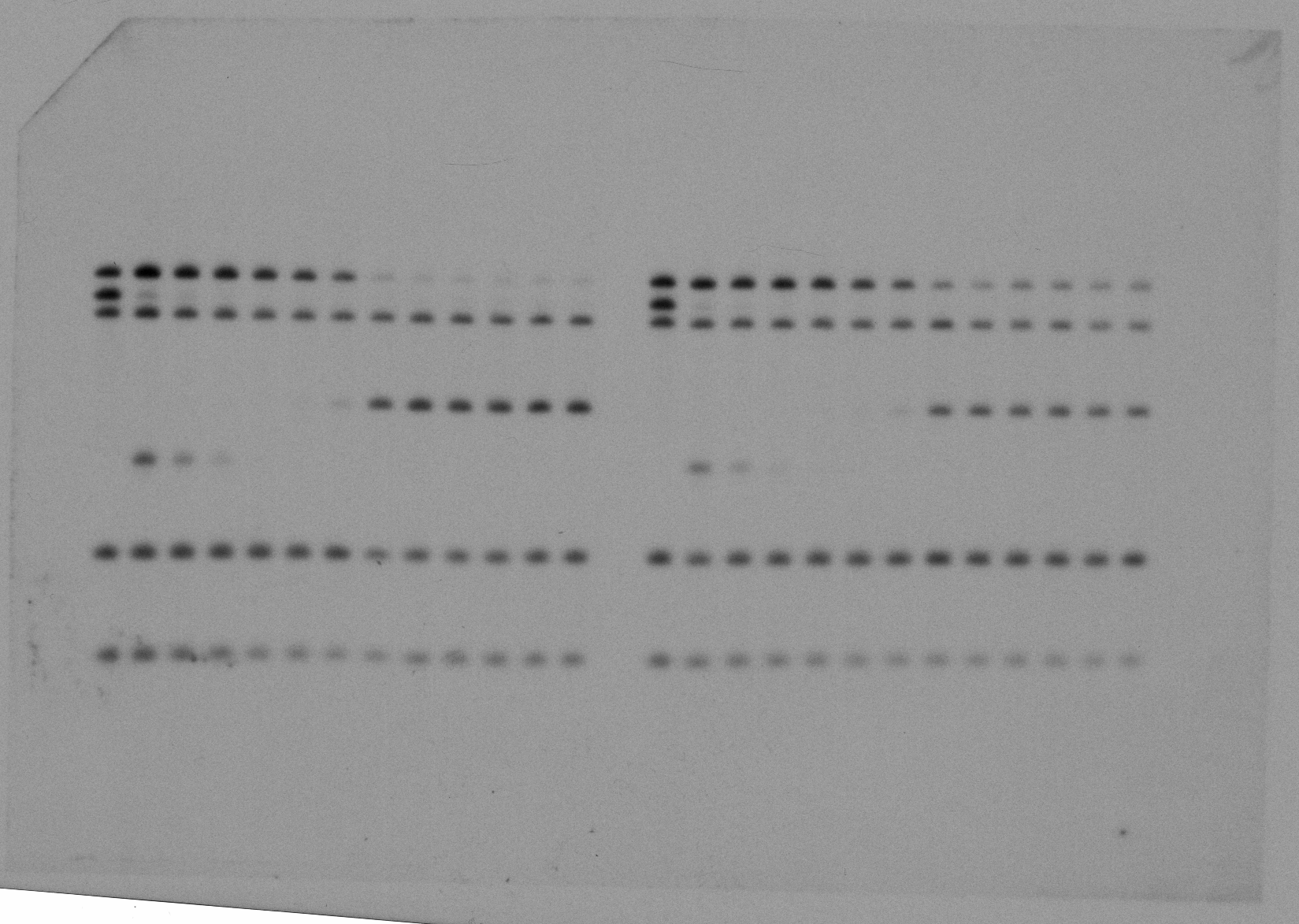

Supplement: Supplementary file 6 — Source Data [file 41467_2023_38417_MOESM6_ESM.zip › Source Data/Uncropped Blots/Figure 2D/Ymv80 wt vs cdc14-1.tif]

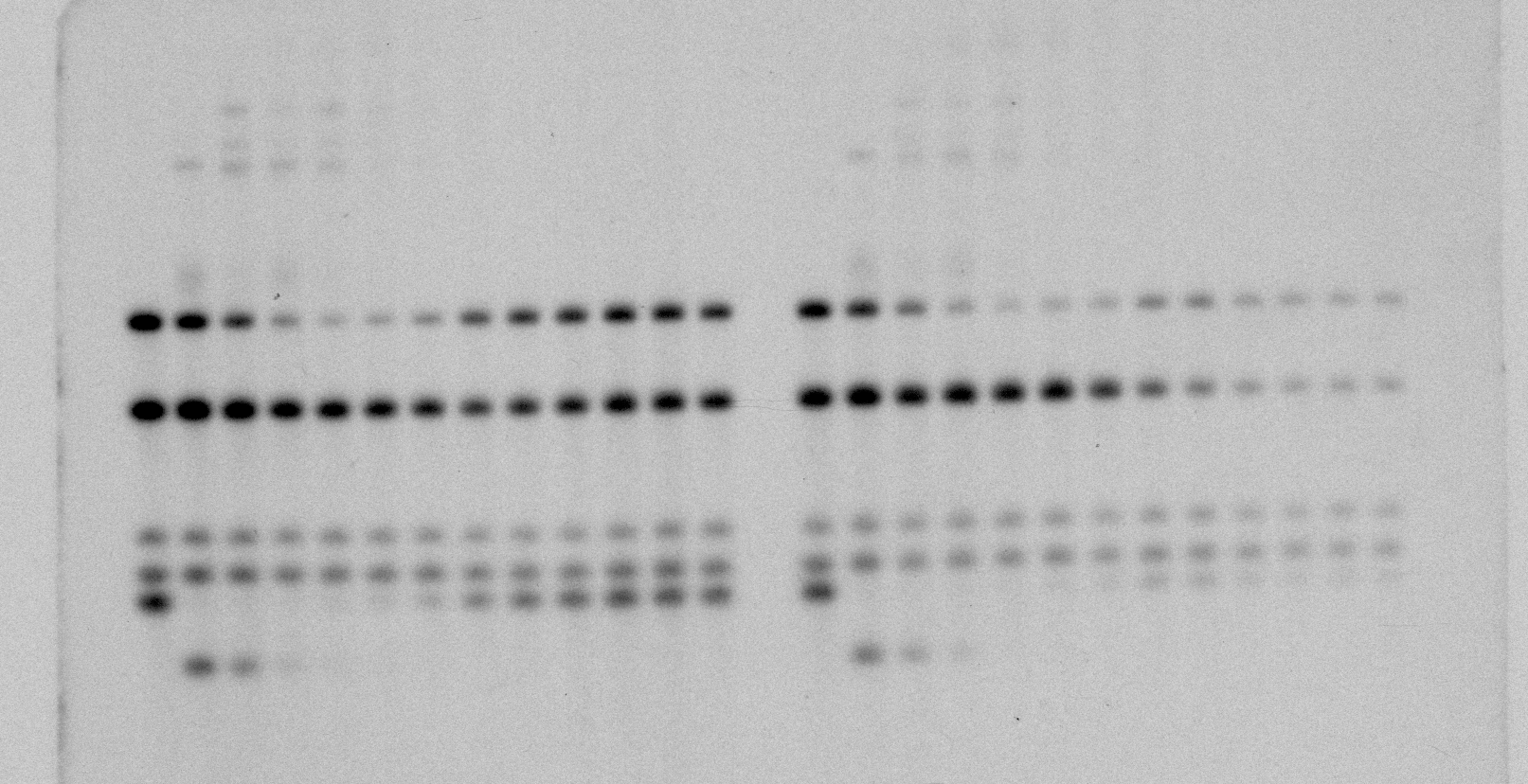

Supplement: Supplementary file 6 — Source Data [file 41467_2023_38417_MOESM6_ESM.zip › Source Data/Uncropped Blots/Figure 4A/0.7 kb, 27 kb, Act1, Mat.tiff]

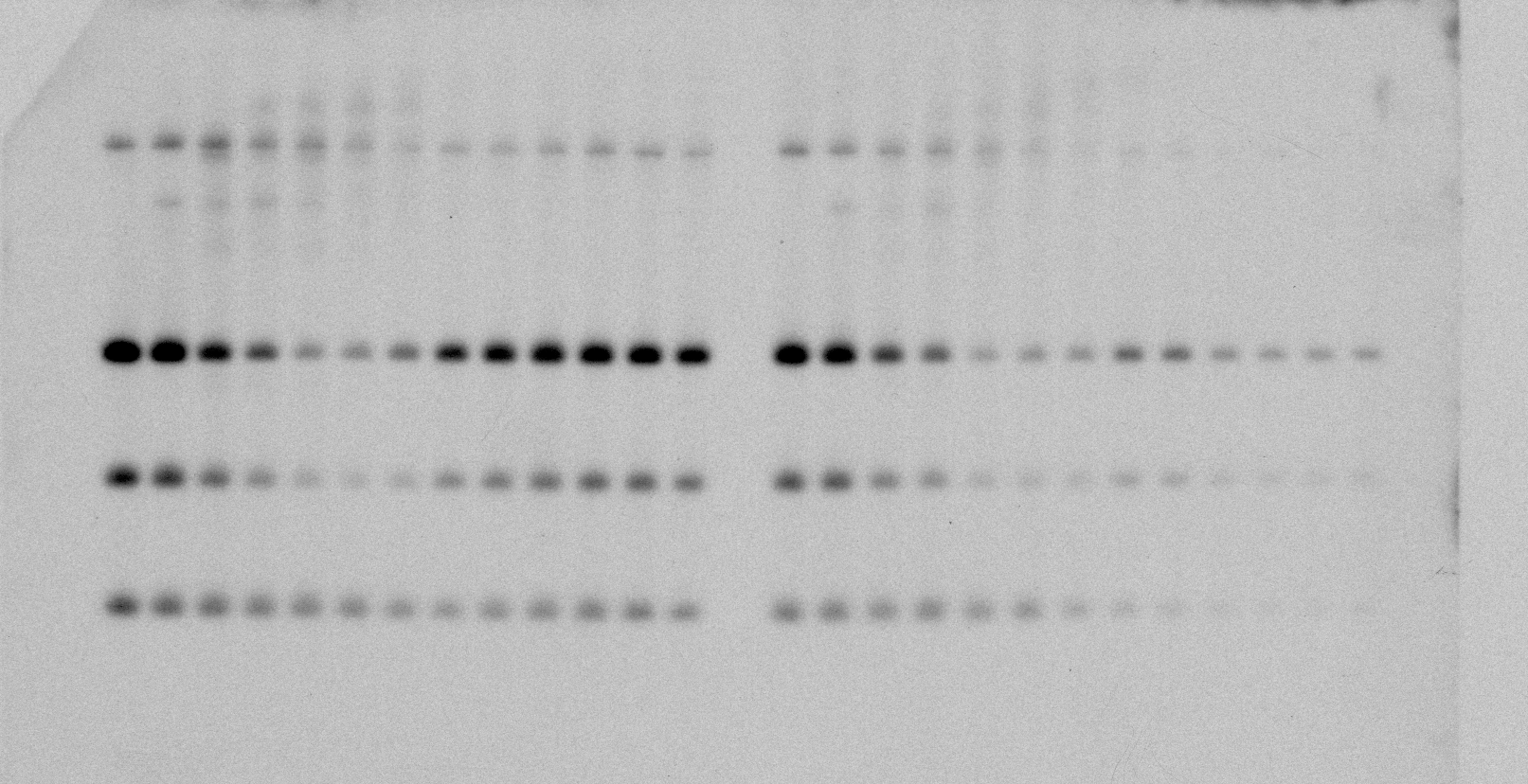

Supplement: Supplementary file 6 — Source Data [file 41467_2023_38417_MOESM6_ESM.zip › Source Data/Uncropped Blots/Figure 4A/3 kb, 6 kb, 21 kb.tiff]

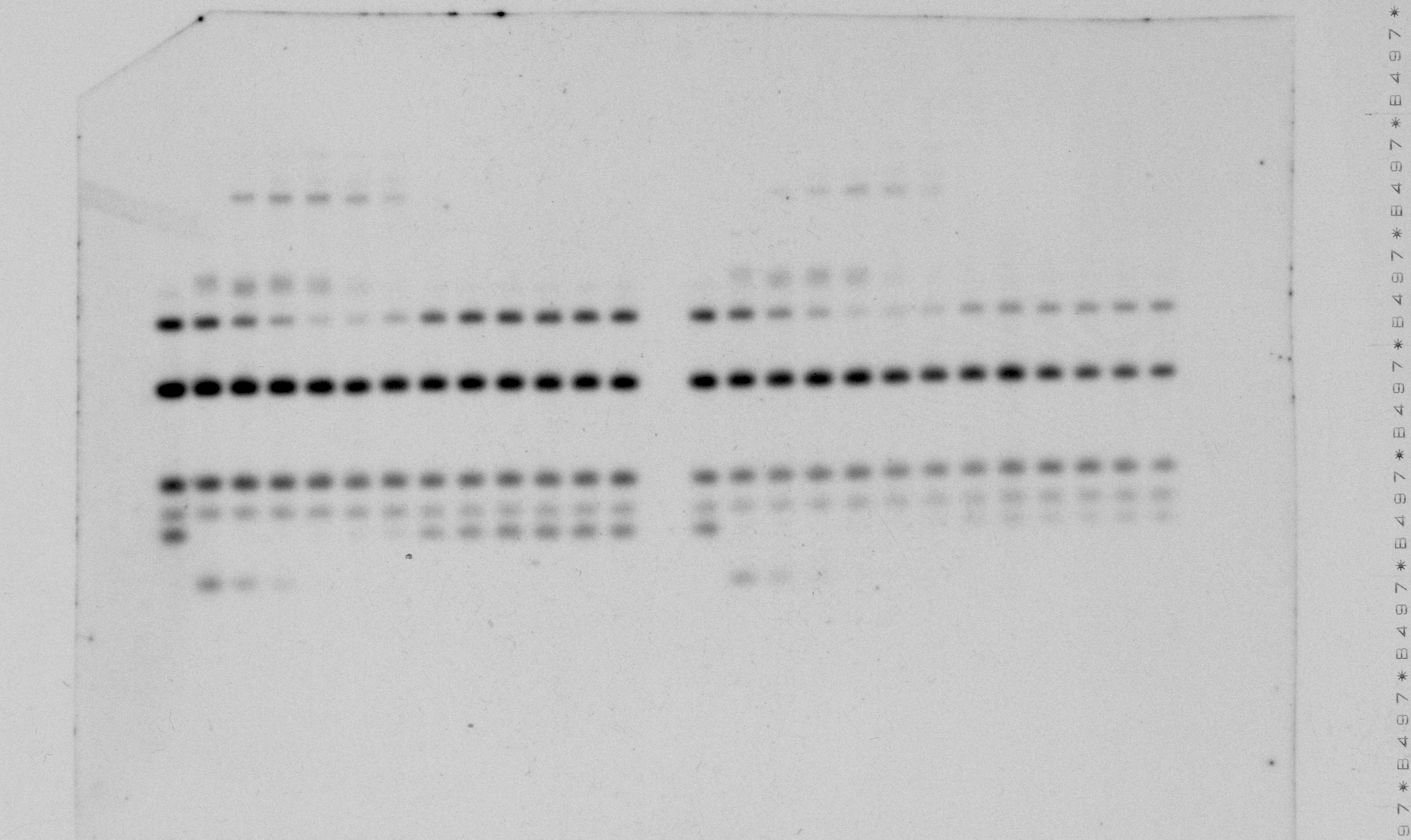

Supplement: Supplementary file 6 — Source Data [file 41467_2023_38417_MOESM6_ESM.zip › Source Data/Uncropped Blots/Supplemental Figure 3C/0.7 kb, 27 kb, Act1, Mat.tiff]

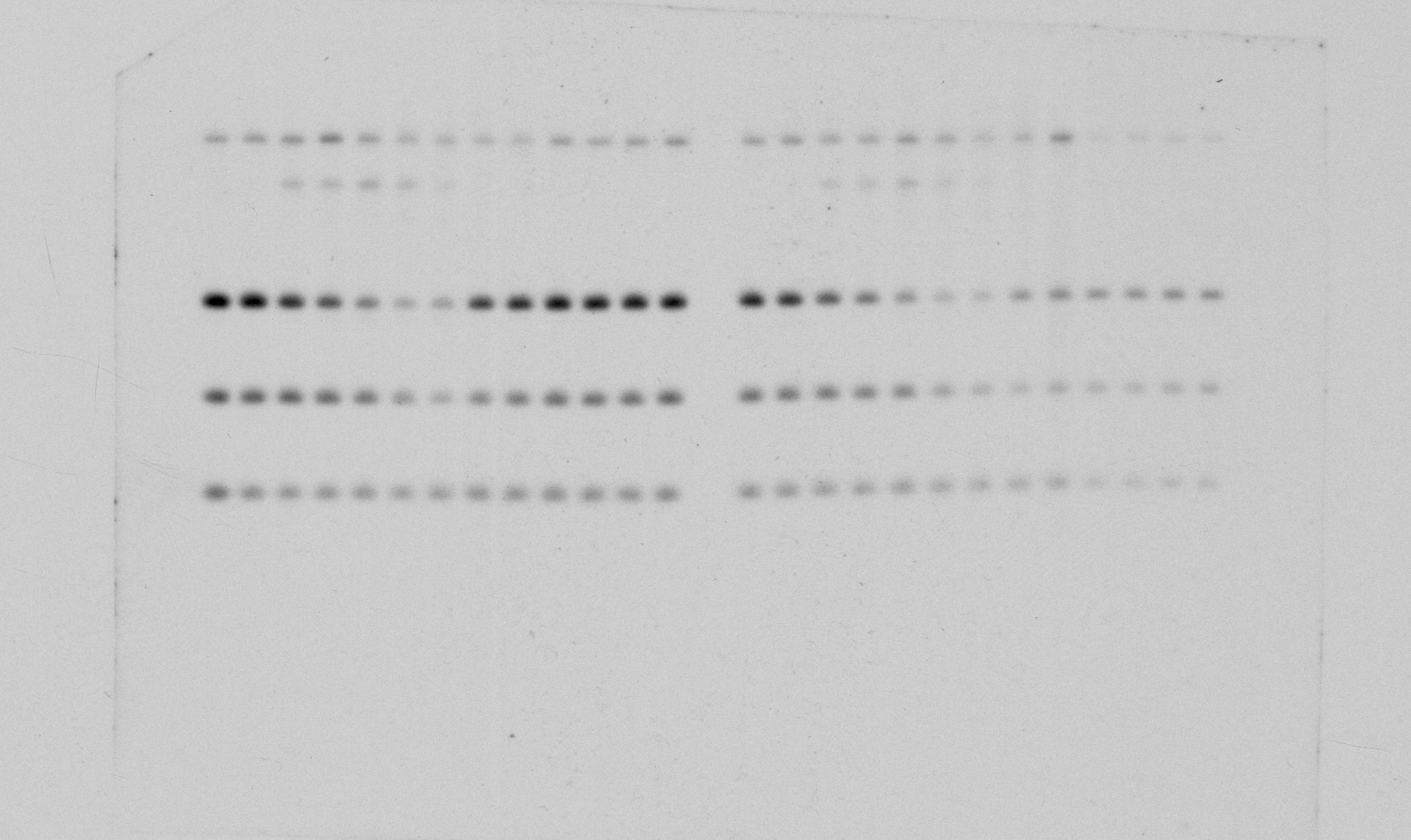

Supplement: Supplementary file 6 — Source Data [file 41467_2023_38417_MOESM6_ESM.zip › Source Data/Uncropped Blots/Supplemental Figure 3C/3 kb, 6 kb, 21 kb.tiff]

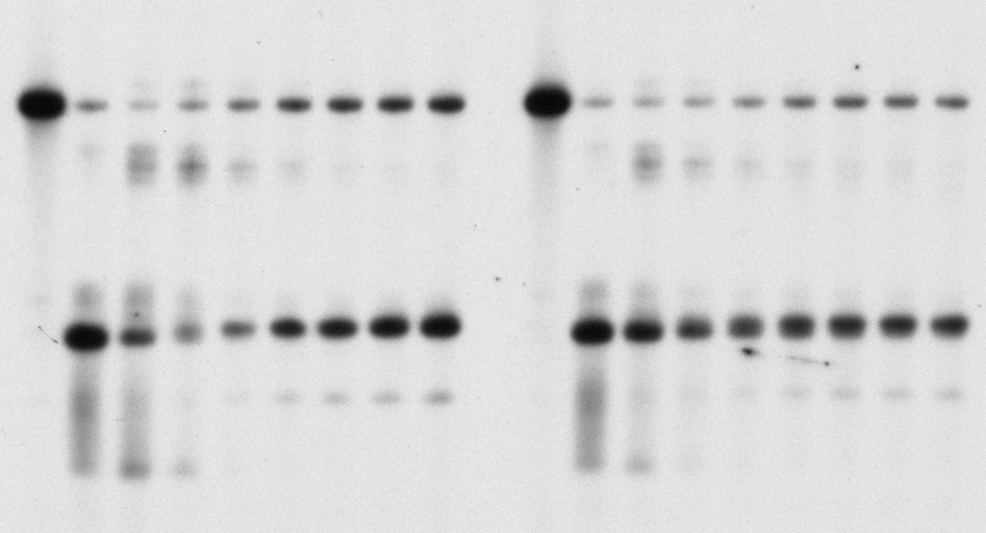

Supplement: Supplementary file 6 — Source Data [file 41467_2023_38417_MOESM6_ESM.zip › Source Data/Uncropped Blots/Figure 1C/Uncropped Film Right.tif]

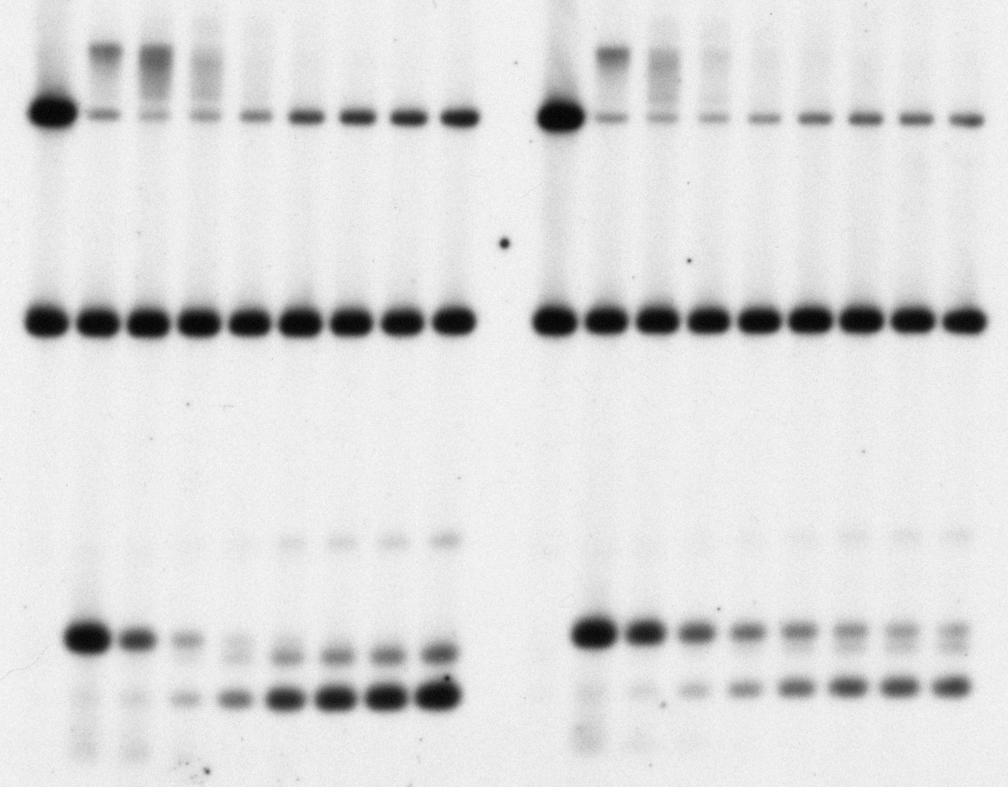

Supplement: Supplementary file 6 — Source Data [file 41467_2023_38417_MOESM6_ESM.zip › Source Data/Uncropped Blots/Figure 1C/Uncropped Film Left.tif]

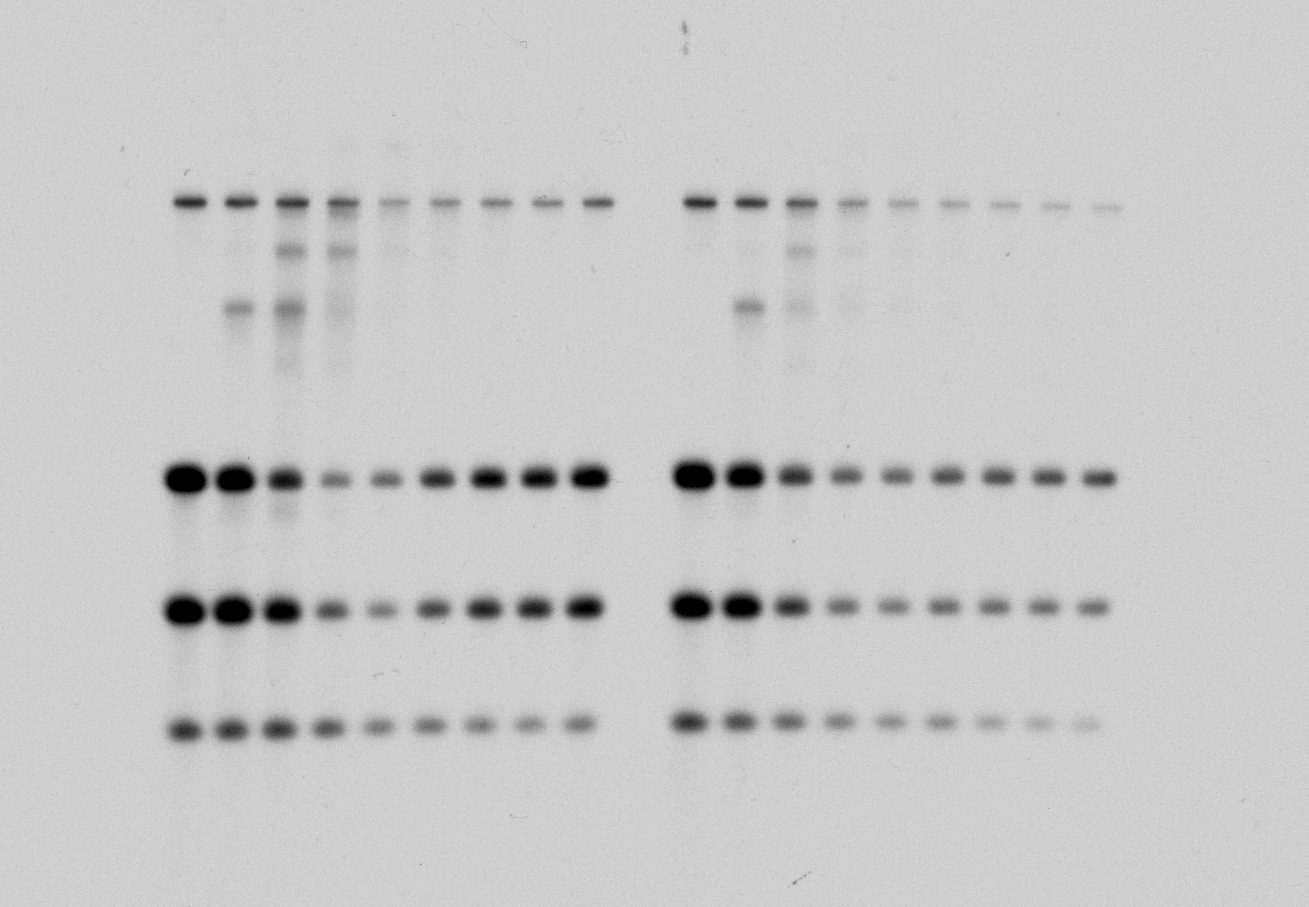

Supplement: Supplementary file 6 — Source Data [file 41467_2023_38417_MOESM6_ESM.zip › Source Data/Uncropped Blots/Figure 2B/3kb, 6kb, 21 kb.tiff]

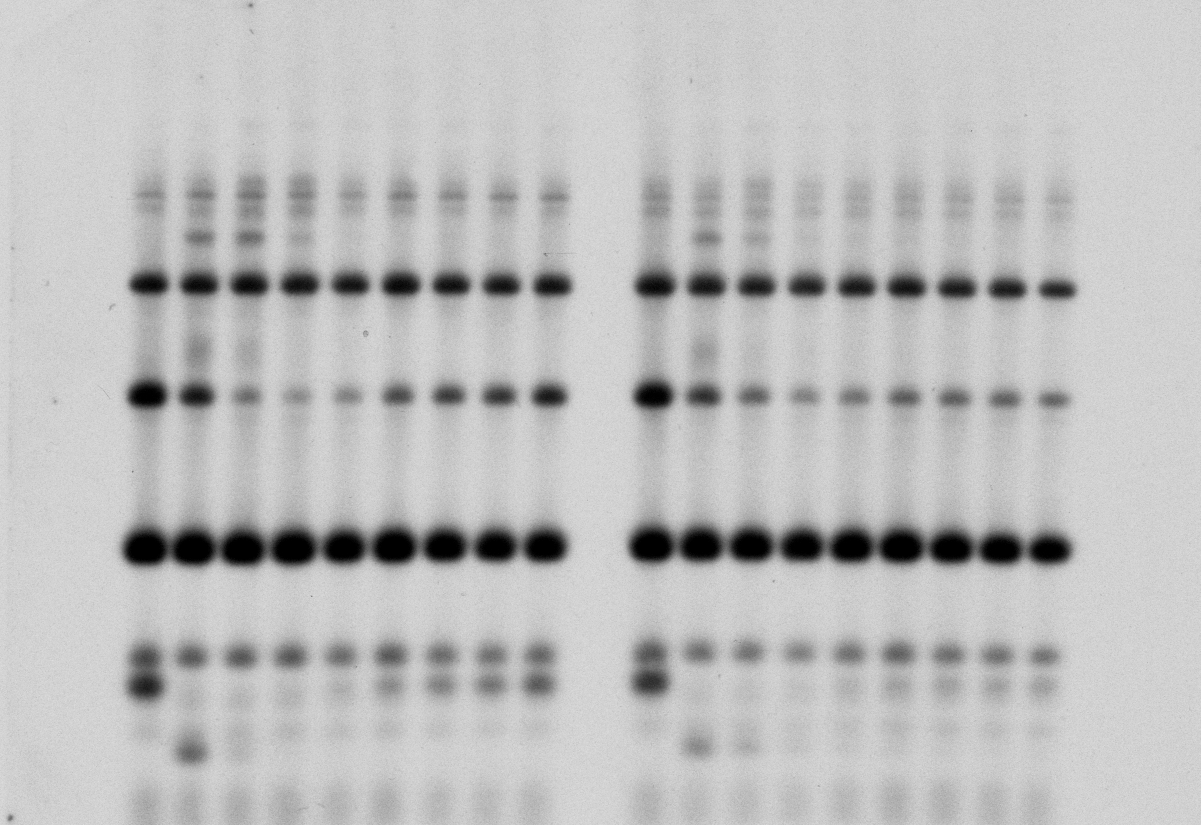

Supplement: Supplementary file 6 — Source Data [file 41467_2023_38417_MOESM6_ESM.zip › Source Data/Uncropped Blots/Figure 2B/0.7 kb, Met5, Mat.tiff]

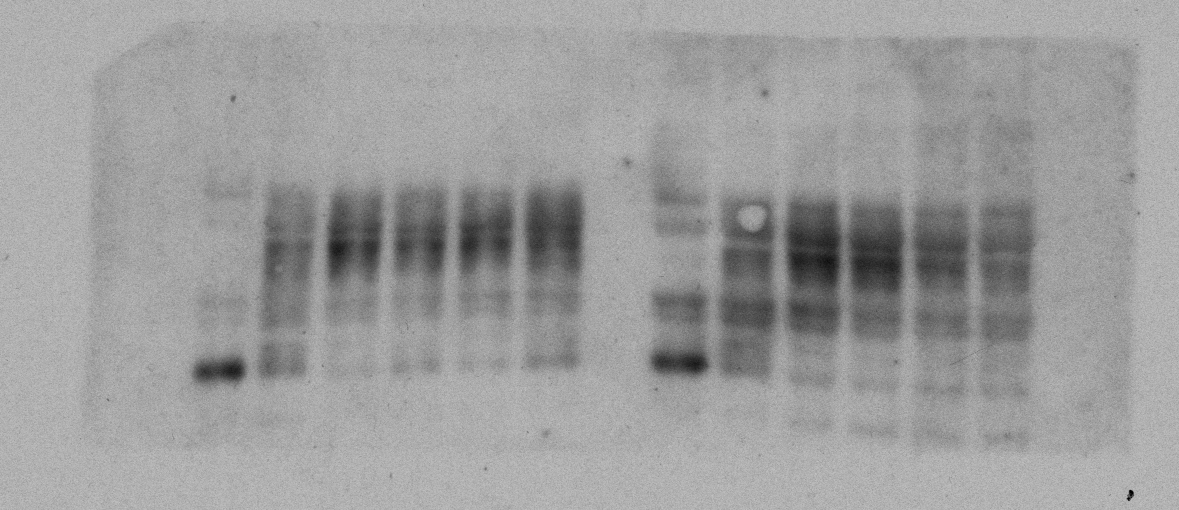

Supplement: Supplementary file 6 — Source Data [file 41467_2023_38417_MOESM6_ESM.zip › Source Data/Uncropped Blots/Figure 7F/aF to Nz Dna2.tif]

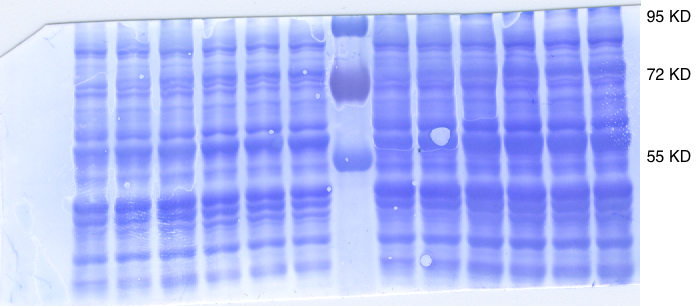

Supplement: Supplementary file 6 — Source Data [file 41467_2023_38417_MOESM6_ESM.zip › Source Data/Uncropped Blots/Figure 7F/commassie Dna2 aF-Nz 2.tiff]

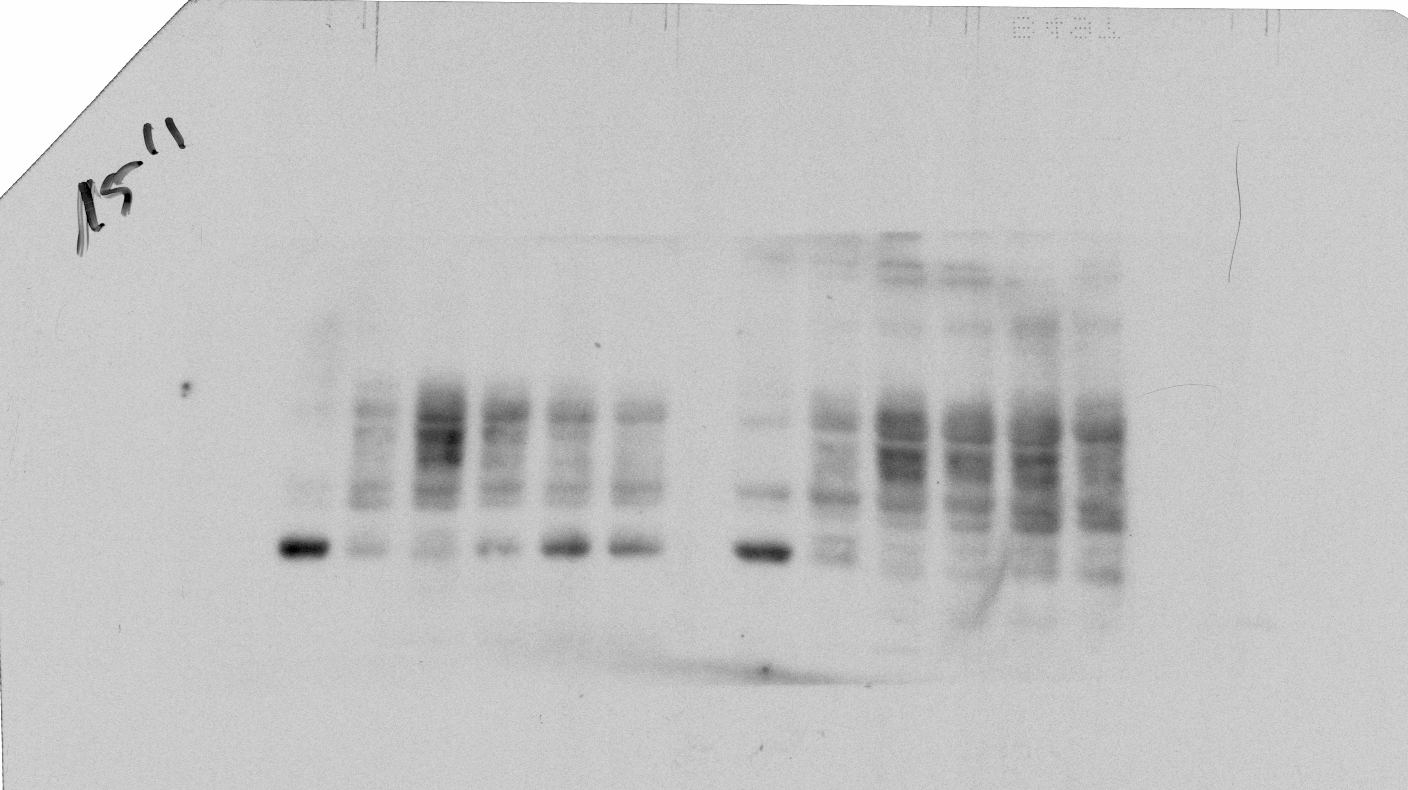

Supplement: Supplementary file 6 — Source Data [file 41467_2023_38417_MOESM6_ESM.zip › Source Data/Uncropped Blots/Figure 7F/aF to aF Dna2.tiff]

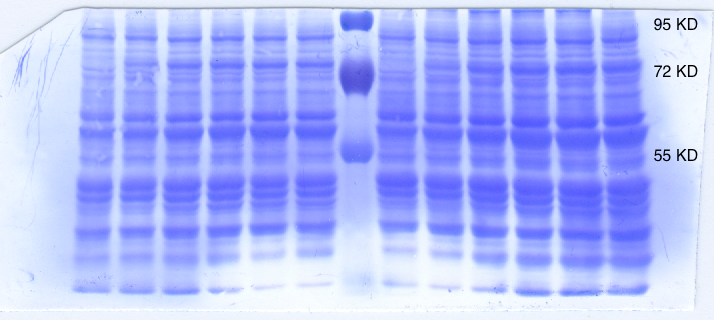

Supplement: Supplementary file 6 — Source Data [file 41467_2023_38417_MOESM6_ESM.zip › Source Data/Uncropped Blots/Figure 7F/commassie Dna2 aF-aF 2.tiff]

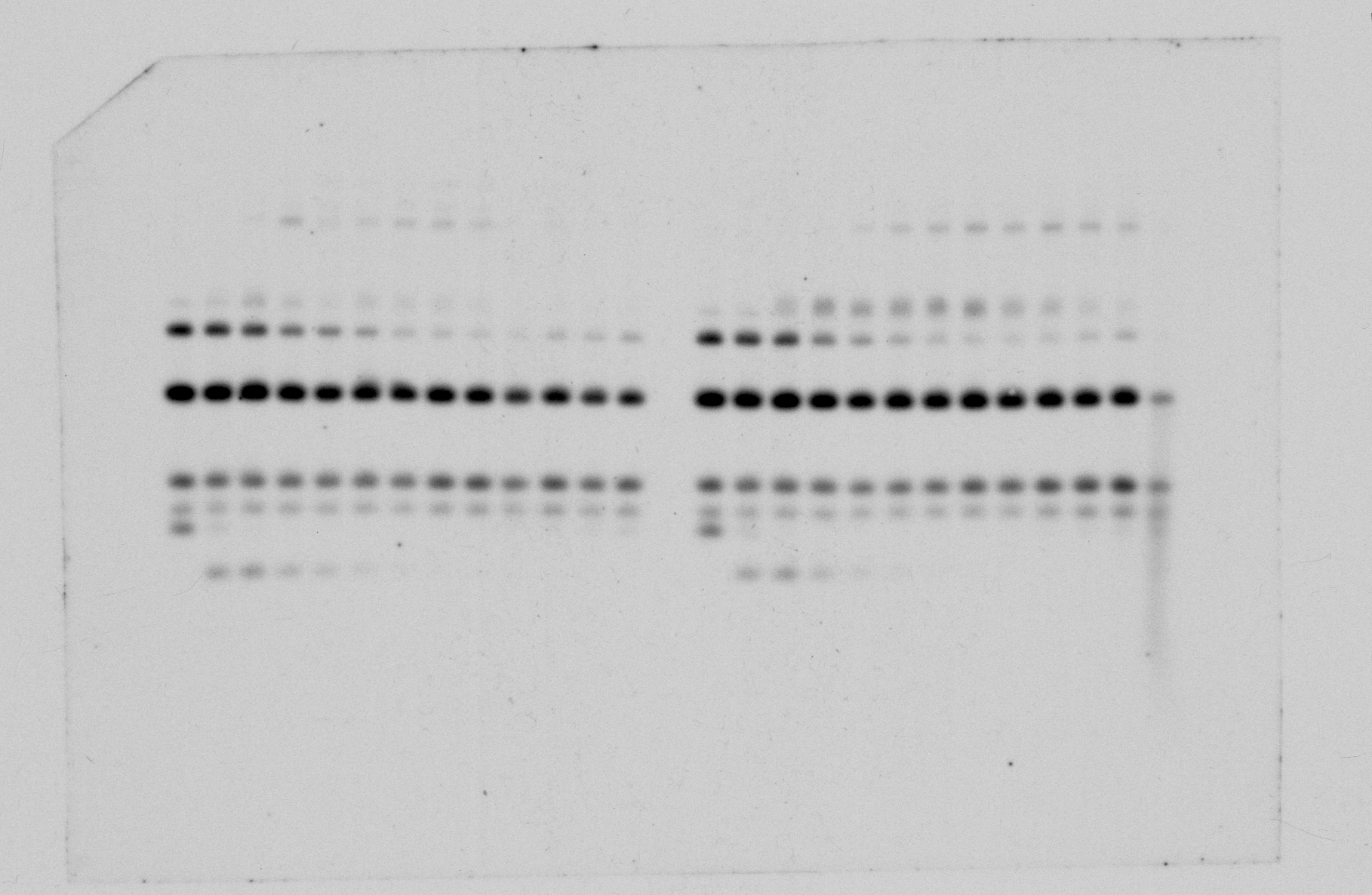

Supplement: Supplementary file 6 — Source Data [file 41467_2023_38417_MOESM6_ESM.zip › Source Data/Uncropped Blots/Supplemental Figure 3B/0.7 kb, 27 kb, Act1, Mat.tiff]

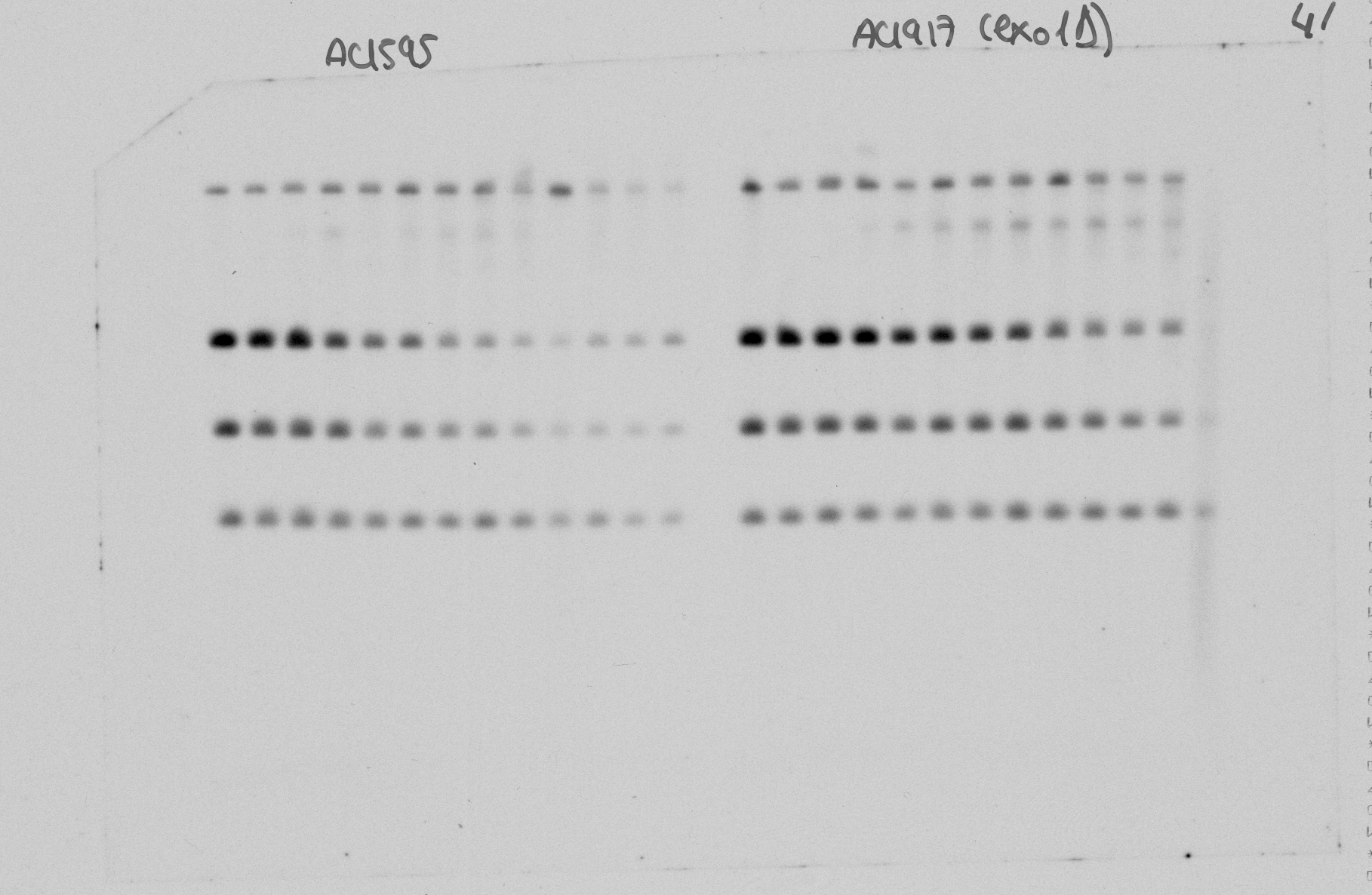

Supplement: Supplementary file 6 — Source Data [file 41467_2023_38417_MOESM6_ESM.zip › Source Data/Uncropped Blots/Supplemental Figure 3B/3 kb, 6 kb, 21 kb.tiff]

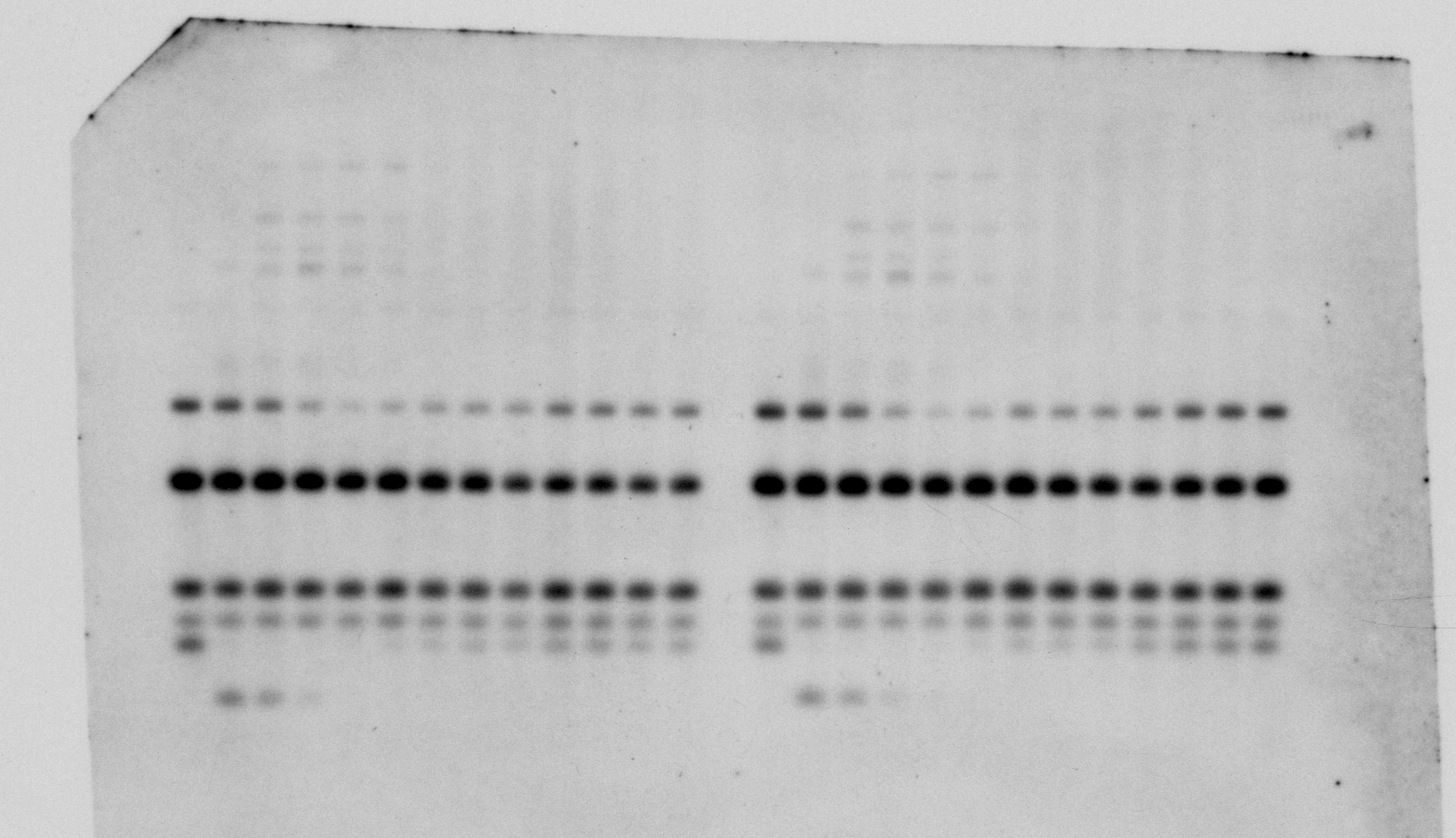

Supplement: Supplementary file 6 — Source Data [file 41467_2023_38417_MOESM6_ESM.zip › Source Data/Uncropped Blots/Figure 6B/Wt 0.7 kb, 27 kb, Act1, Mat.tiff]

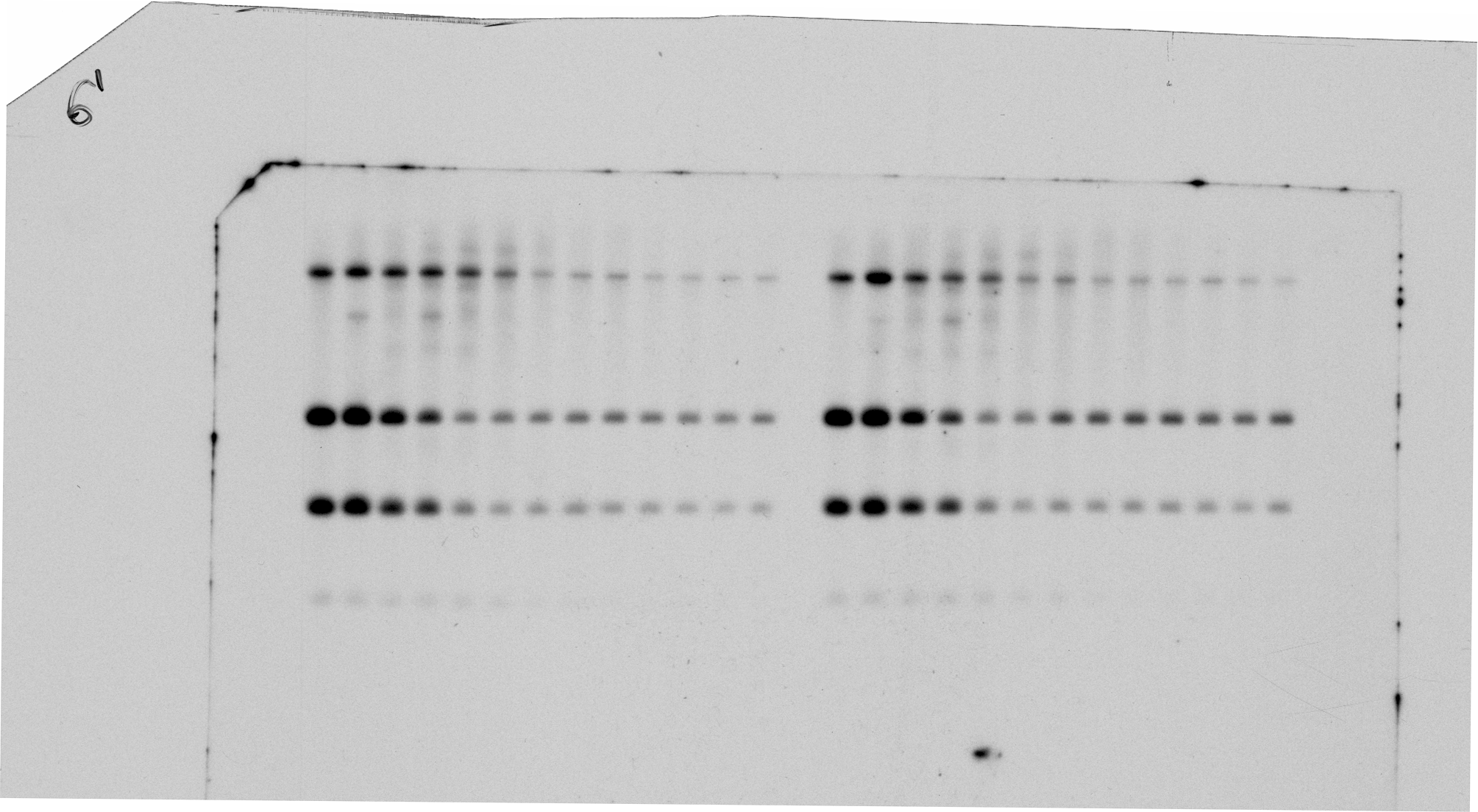

Supplement: Supplementary file 6 — Source Data [file 41467_2023_38417_MOESM6_ESM.zip › Source Data/Uncropped Blots/Figure 6B/cdc14-1 3 kb, 6 kb, 21 kb.tiff]

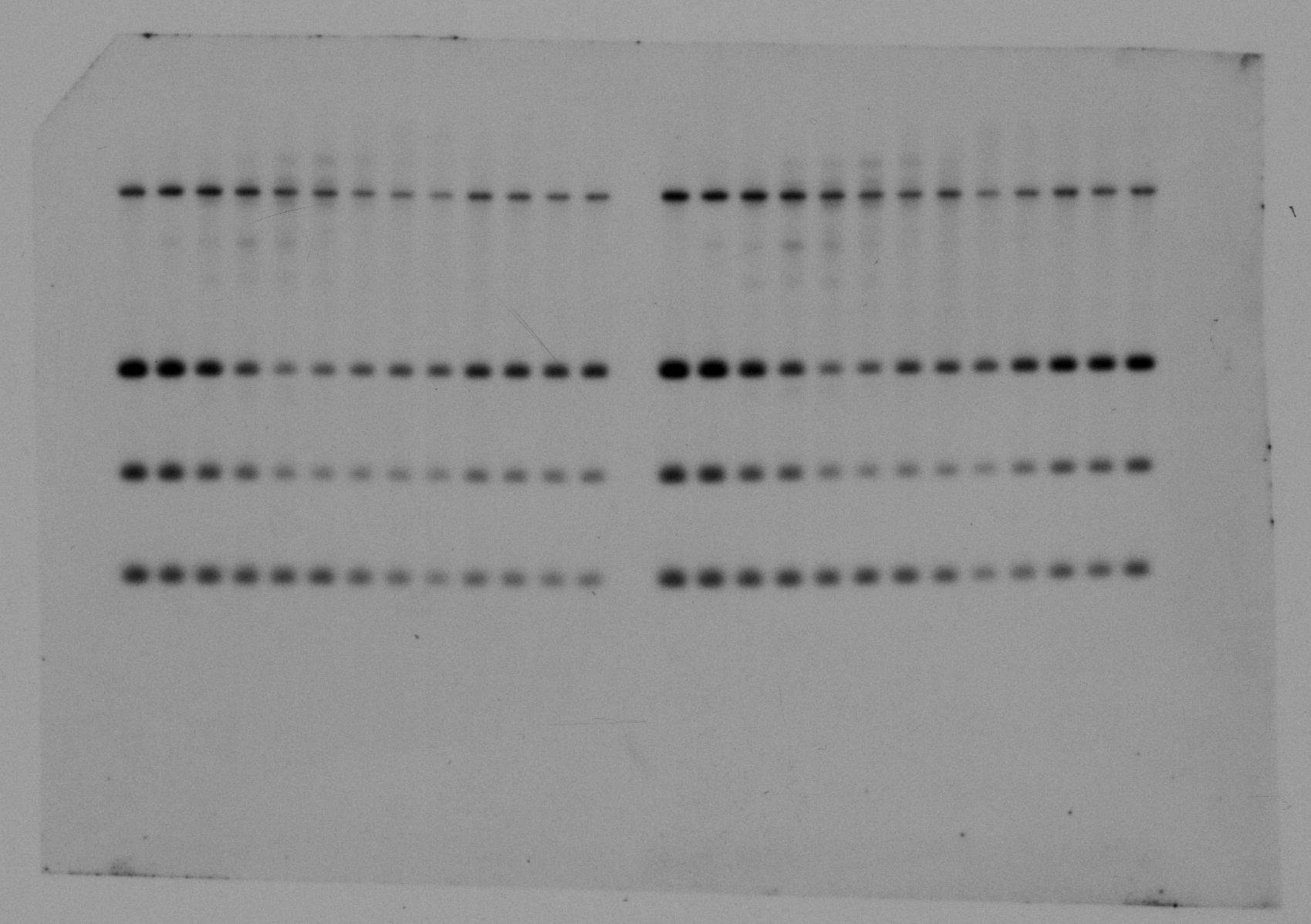

Supplement: Supplementary file 6 — Source Data [file 41467_2023_38417_MOESM6_ESM.zip › Source Data/Uncropped Blots/Figure 6B/Wt 3 kb, 6 kb, 21 kb.tif]

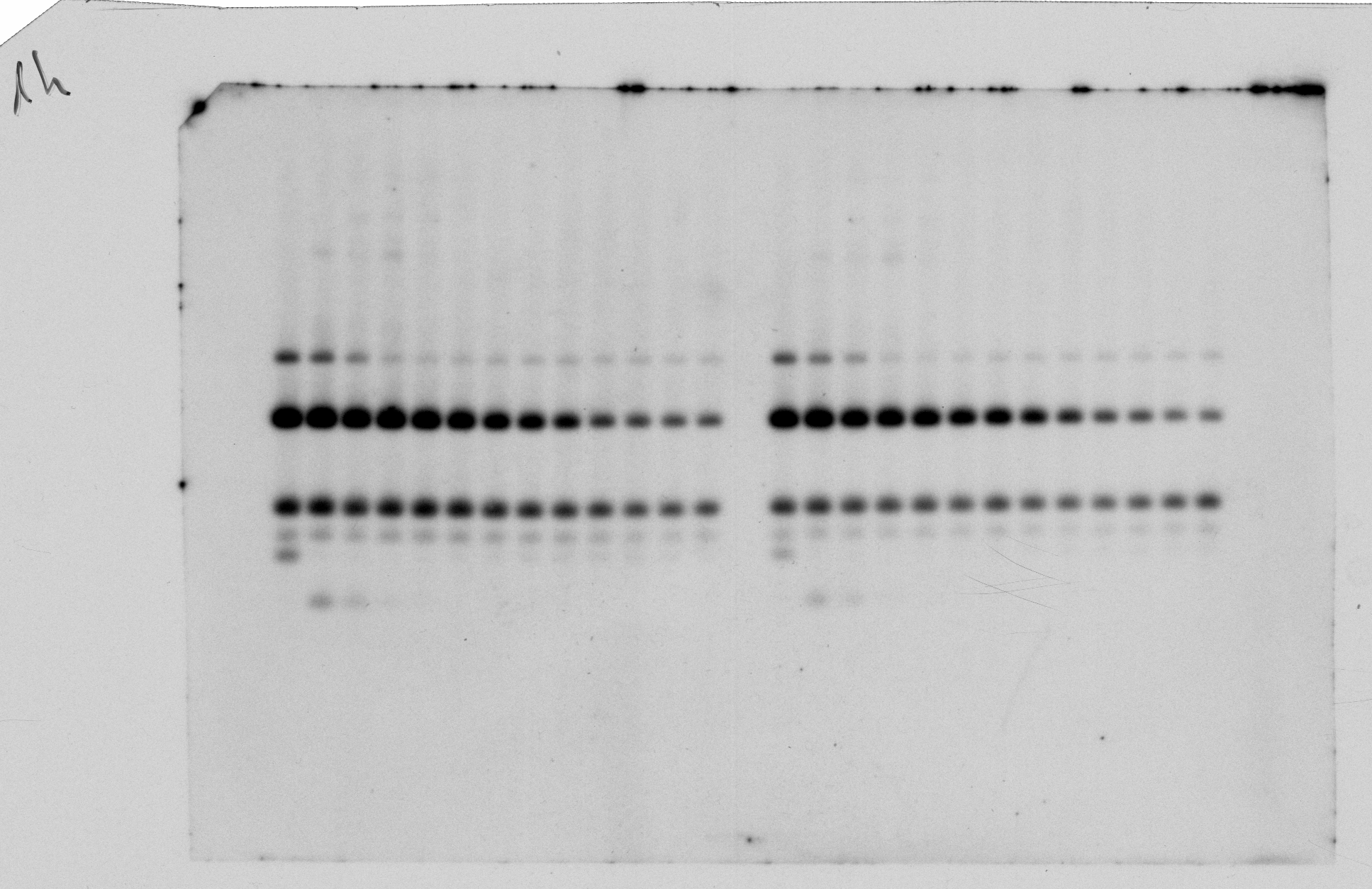

Supplement: Supplementary file 6 — Source Data [file 41467_2023_38417_MOESM6_ESM.zip › Source Data/Uncropped Blots/Figure 6B/cdc14-1 0.7 kb, 27 kb, Act1, Mat.tiff]

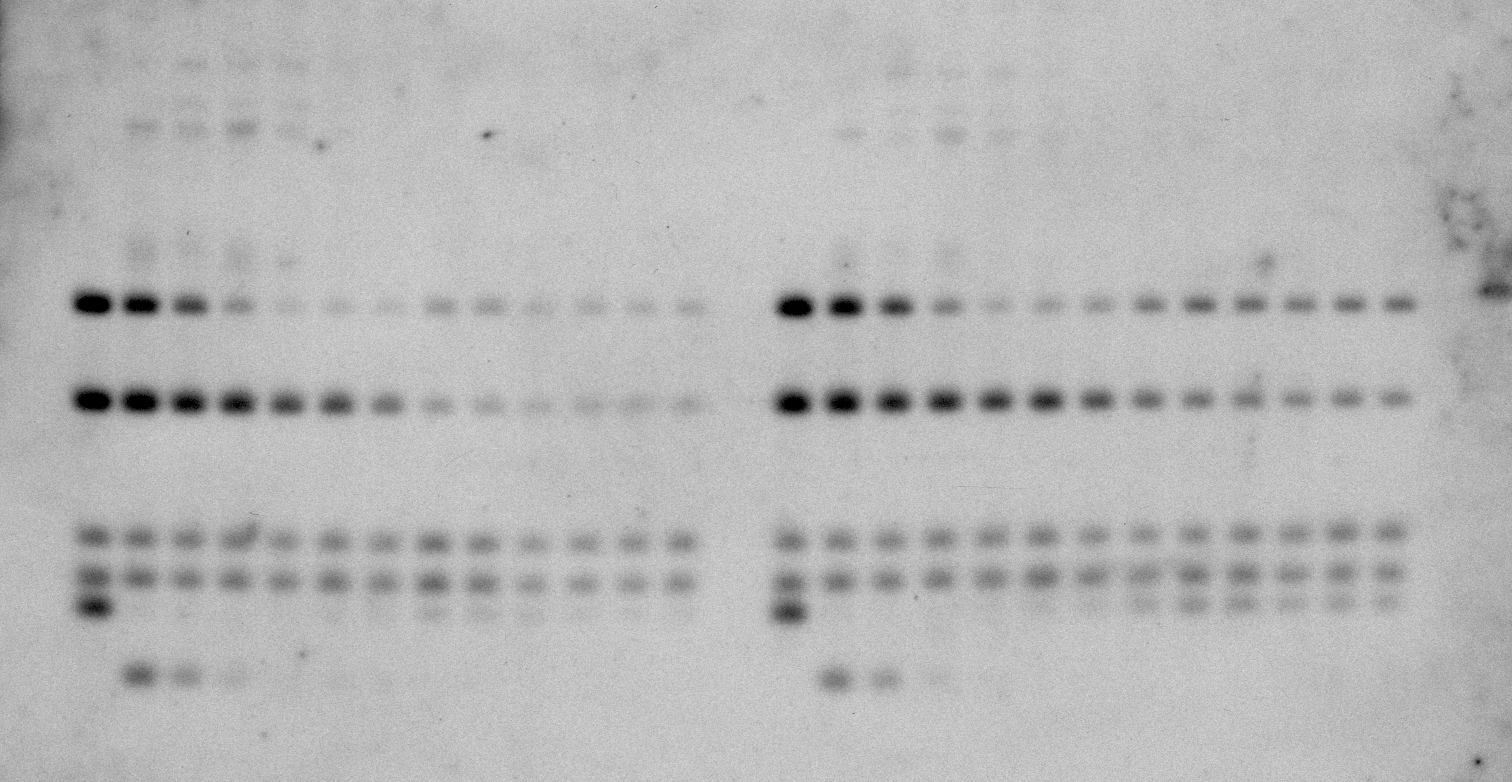

Supplement: Supplementary file 6 — Source Data [file 41467_2023_38417_MOESM6_ESM.zip › Source Data/Uncropped Blots/Figure 5C/0.7 kb, 27 kb, Act1, Mat.tiff]

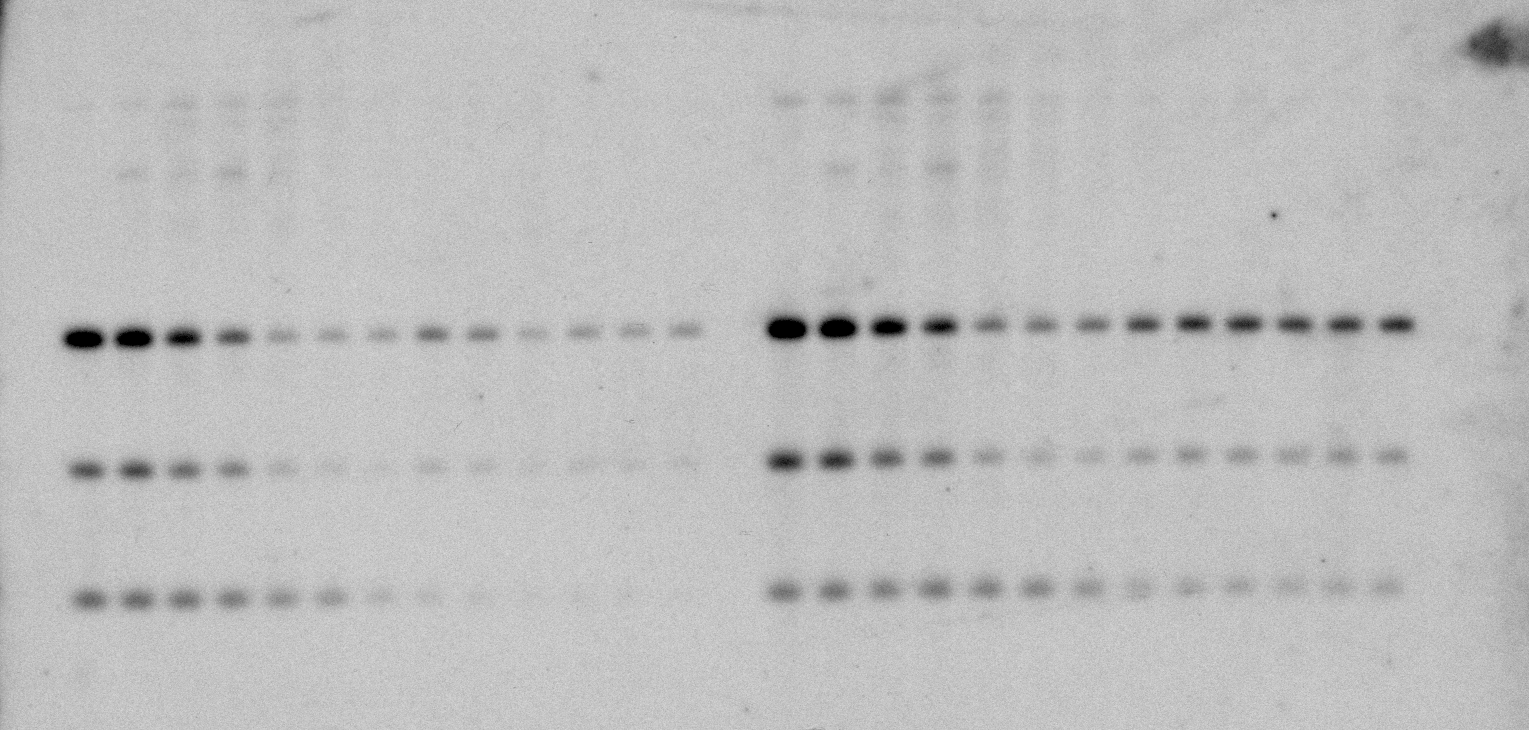

Supplement: Supplementary file 6 — Source Data [file 41467_2023_38417_MOESM6_ESM.zip › Source Data/Uncropped Blots/Figure 5C/3 kb, 6 kb, 21 kb.tiff]

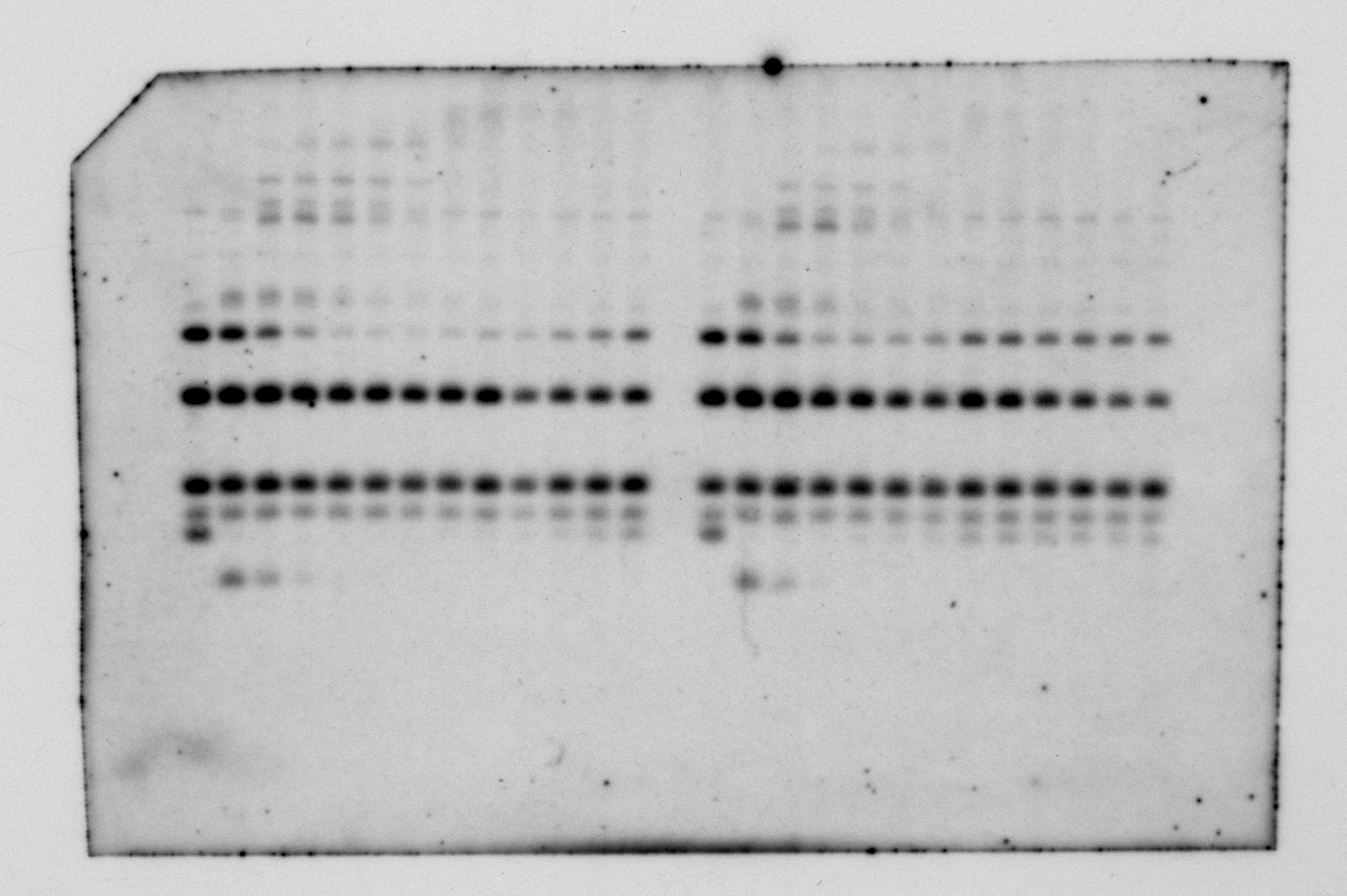

Supplement: Supplementary file 6 — Source Data [file 41467_2023_38417_MOESM6_ESM.zip › Source Data/Uncropped Blots/Supplemental Figure 4D/0.7 kb, 27 kb, Act1, Mat.tiff]

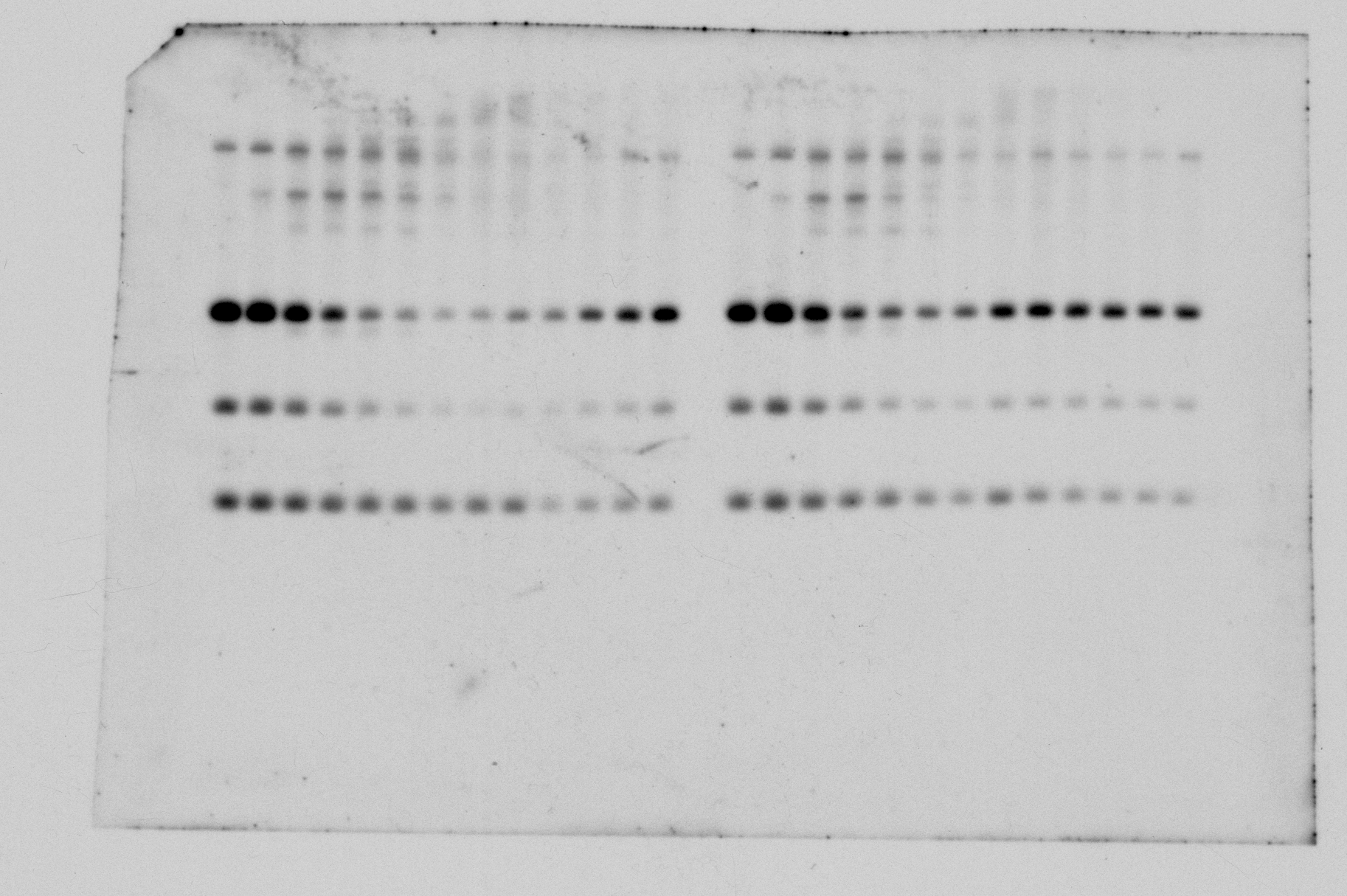

Supplement: Supplementary file 6 — Source Data [file 41467_2023_38417_MOESM6_ESM.zip › Source Data/Uncropped Blots/Supplemental Figure 4D/3 kb, 6 kb, 21 kb.tiff]

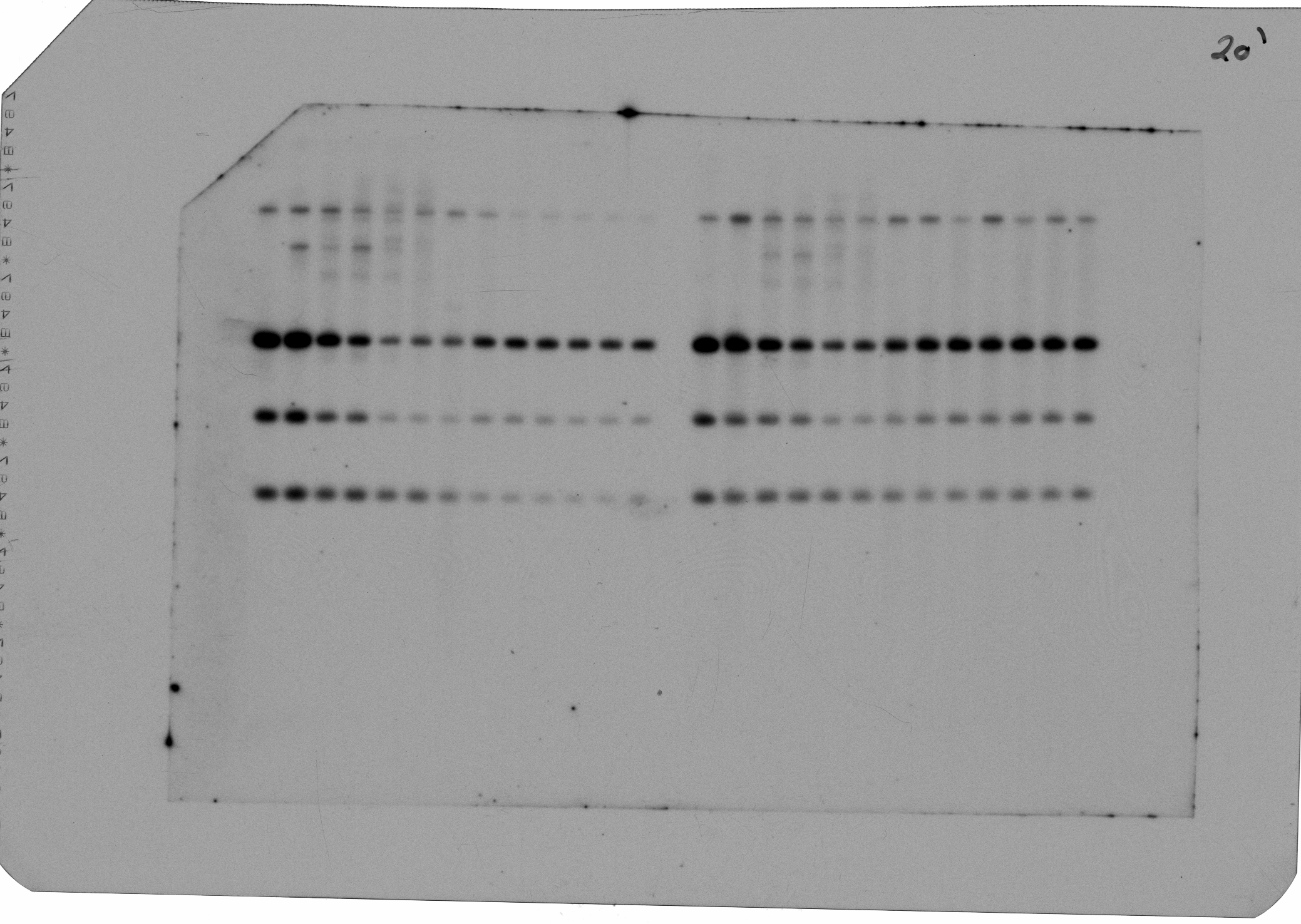

Supplement: Supplementary file 6 — Source Data [file 41467_2023_38417_MOESM6_ESM.zip › Source Data/Uncropped Blots/Supplemental Figure 4C/3 kb, 6 kb, 21 kb.tif]

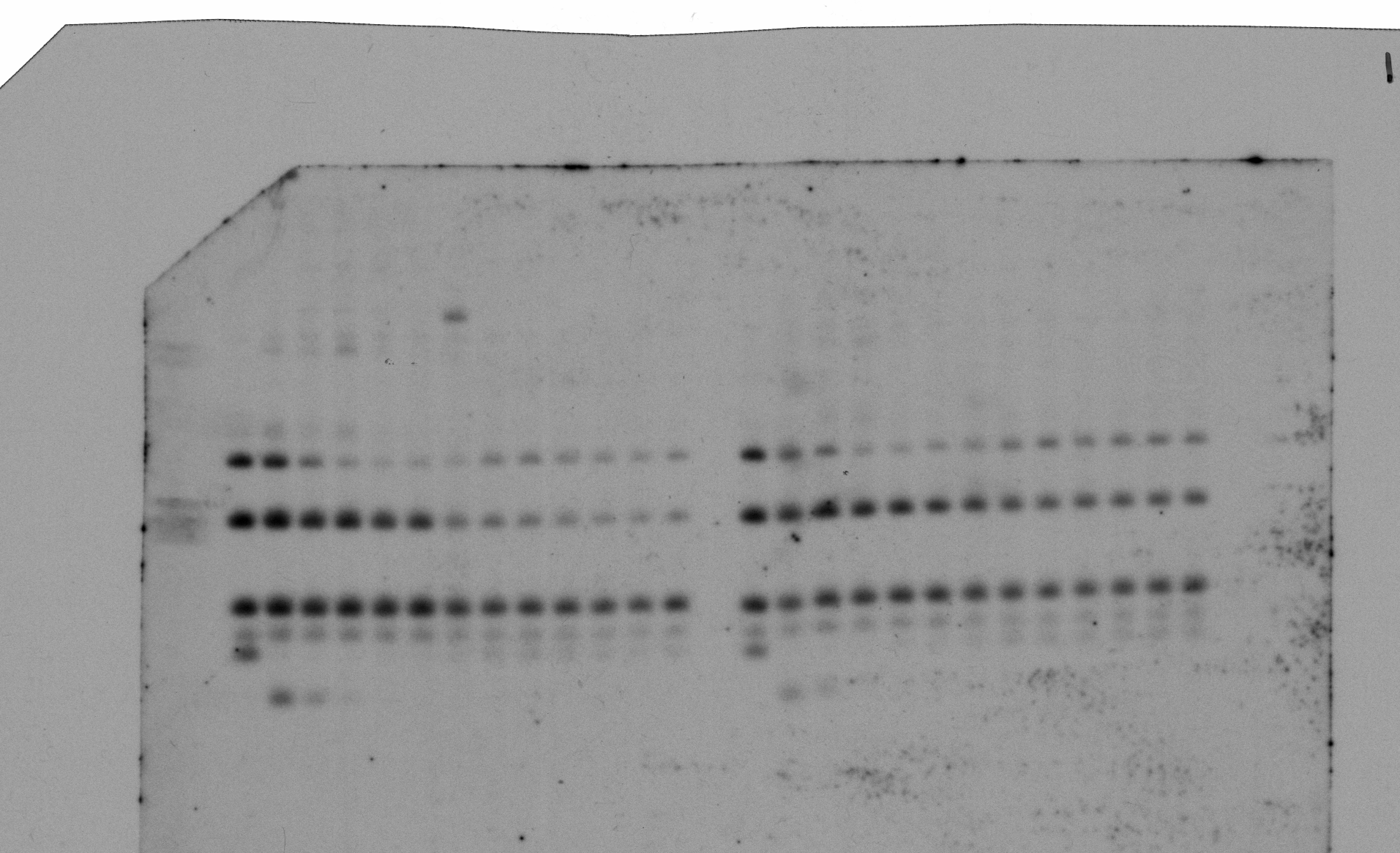

Supplement: Supplementary file 6 — Source Data [file 41467_2023_38417_MOESM6_ESM.zip › Source Data/Uncropped Blots/Supplemental Figure 4C/0.7 kb, 27 kb, Act1, Mat.tif]

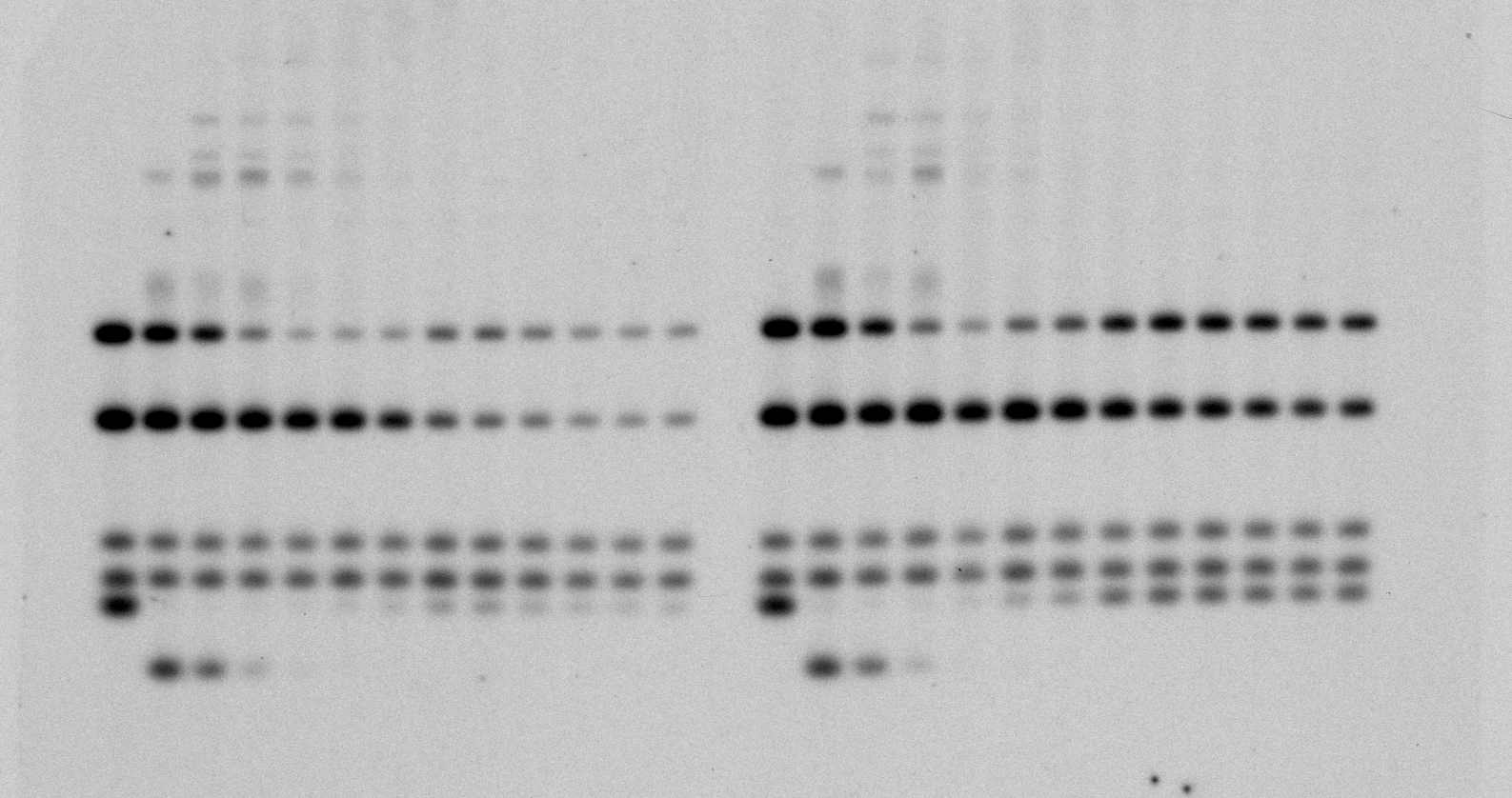

Supplement: Supplementary file 6 — Source Data [file 41467_2023_38417_MOESM6_ESM.zip › Source Data/Uncropped Blots/Figure 5B/0.7 kb, 27 kb, Act1, Mat.tiff]

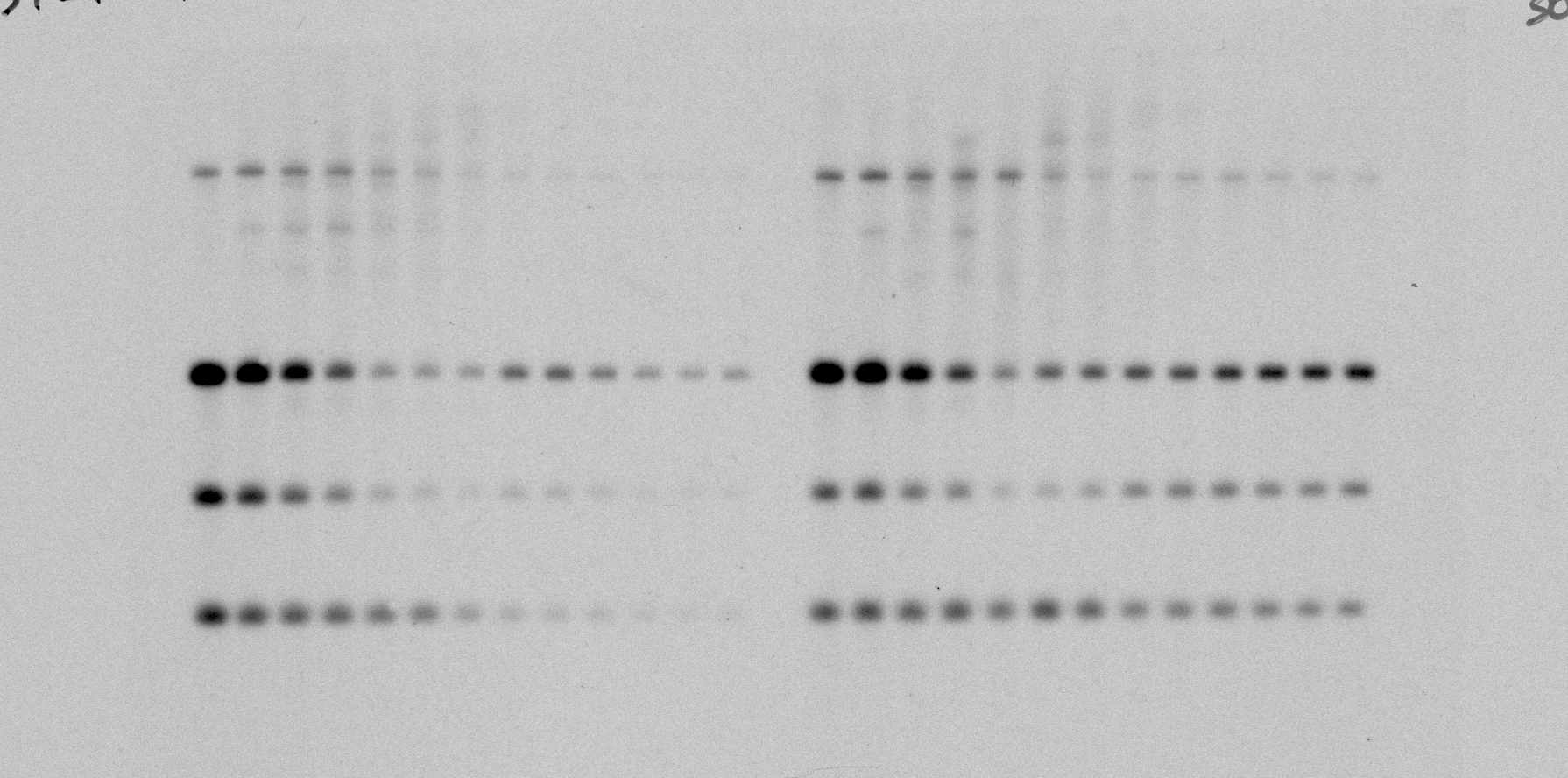

Supplement: Supplementary file 6 — Source Data [file 41467_2023_38417_MOESM6_ESM.zip › Source Data/Uncropped Blots/Figure 5B/3 kb, 6 kb, 21 kb.tiff]

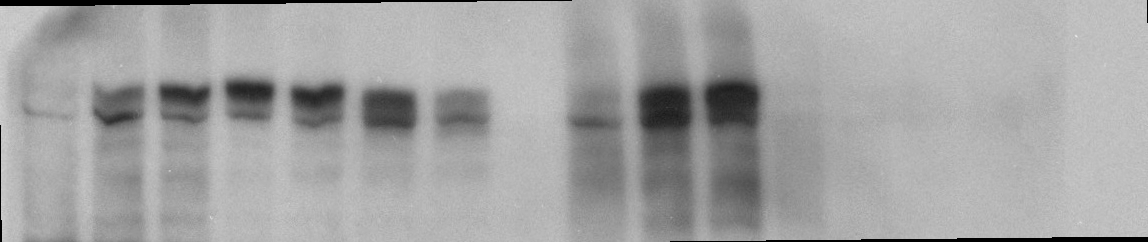

Supplement: Supplementary file 6 — Source Data [file 41467_2023_38417_MOESM6_ESM.zip › Source Data/Uncropped Blots/Supplemental Figure 4B/Dna2.tif]

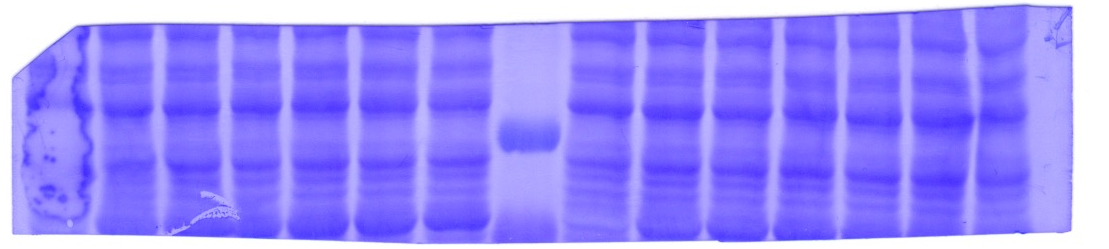

Supplement: Supplementary file 6 — Source Data [file 41467_2023_38417_MOESM6_ESM.zip › Source Data/Uncropped Blots/Supplemental Figure 4B/Rad53_coomassie membrane.tif]

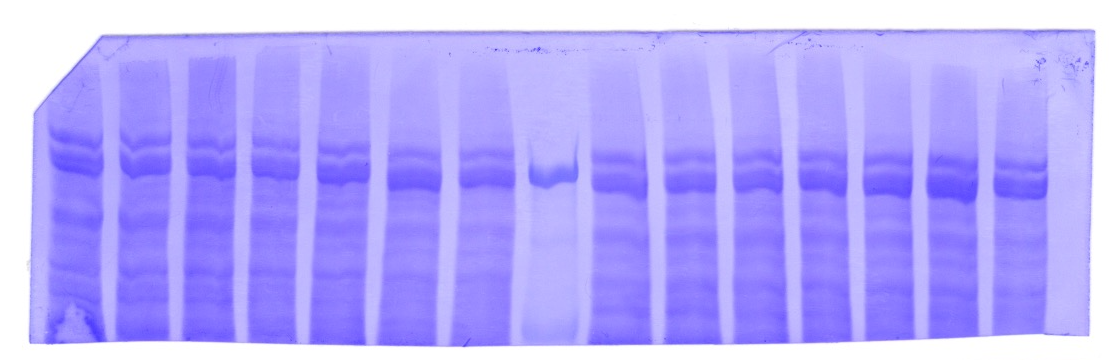

Supplement: Supplementary file 6 — Source Data [file 41467_2023_38417_MOESM6_ESM.zip › Source Data/Uncropped Blots/Supplemental Figure 4B/Dna2_coomassie membrane_1.tif]

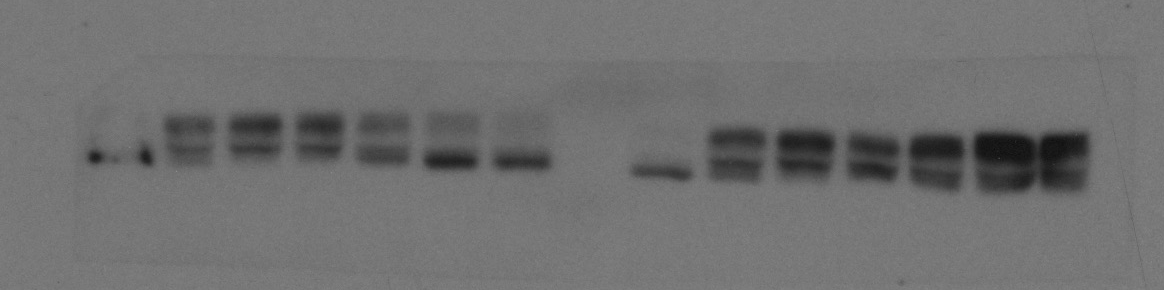

Supplement: Supplementary file 6 — Source Data [file 41467_2023_38417_MOESM6_ESM.zip › Source Data/Uncropped Blots/Supplemental Figure 4B/Rad53.tif]
